# Supplementary material for: Global connections between El Nino and landslide impacts
Source: Nat Commun. 2021 Apr 15;12:2262. doi: 10.1038/s41467-021-22398-4 (PMC8050240; doi:10.1038/s41467-021-22398-4)
Supplement: Supplementary file 1 — Supplementary Information [file 41467_2021_22398_MOESM1_ESM.pdf]

Supplementary Information, Emberson et al. 2021: Global connections between El Nino and landslide impacts

Contents

1. Supplementary Figures 1-10

2. Supplementary Tables 1 and 2

3. Supplementary Notes:

Supplementary note 1: Significance of relationship between MVEI and landslide exposure model components. This provides context for supplementary figures 1-10 and supplementary table 1.

Supplementary Note 2: Validation of model outputs with landslide fatality data explanation

1. Supplementary Figures

In the figures below, we map the p-values for each of the parameters listed in supplementary table 1. All calculated p-value data are also available in supplementary data tables. See supplementary note 1 for context (below)

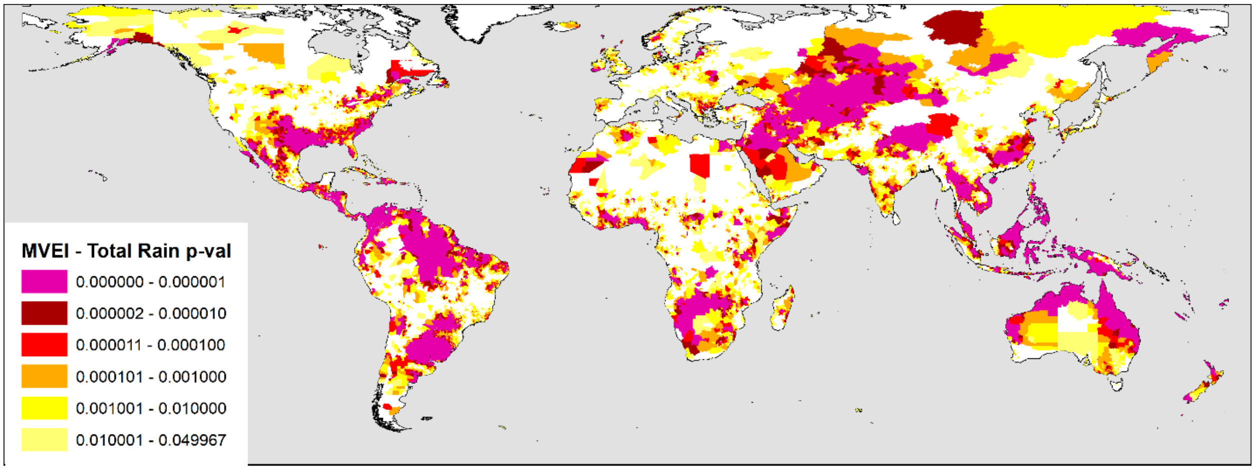

Supplementary Figure 1: A – P-values for relationship between smoothed MVEI and total monthly rainfall

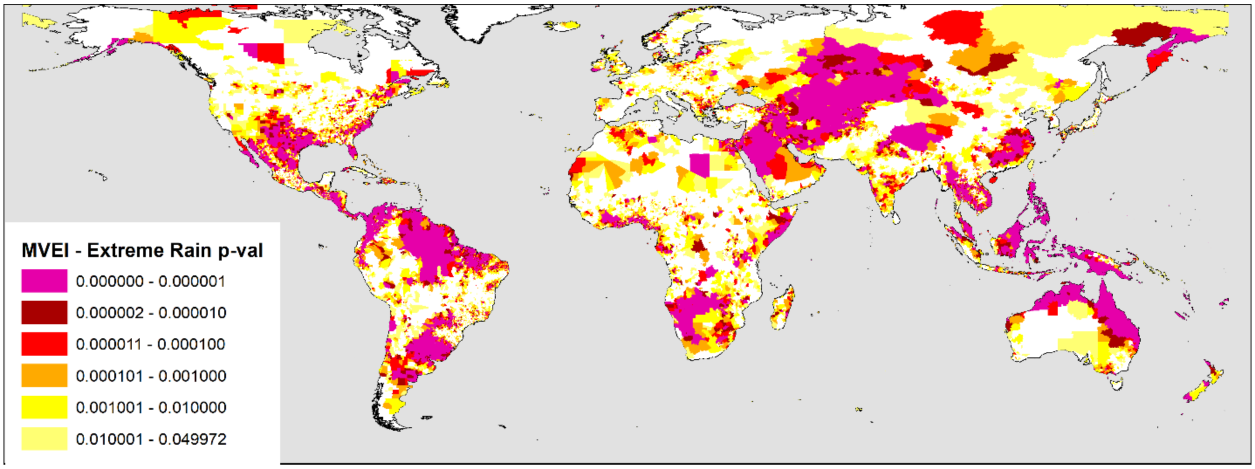

Supplementary Figure 2: B – P-values for relationship between smoothed MVEI and number of extreme rainfall days per month

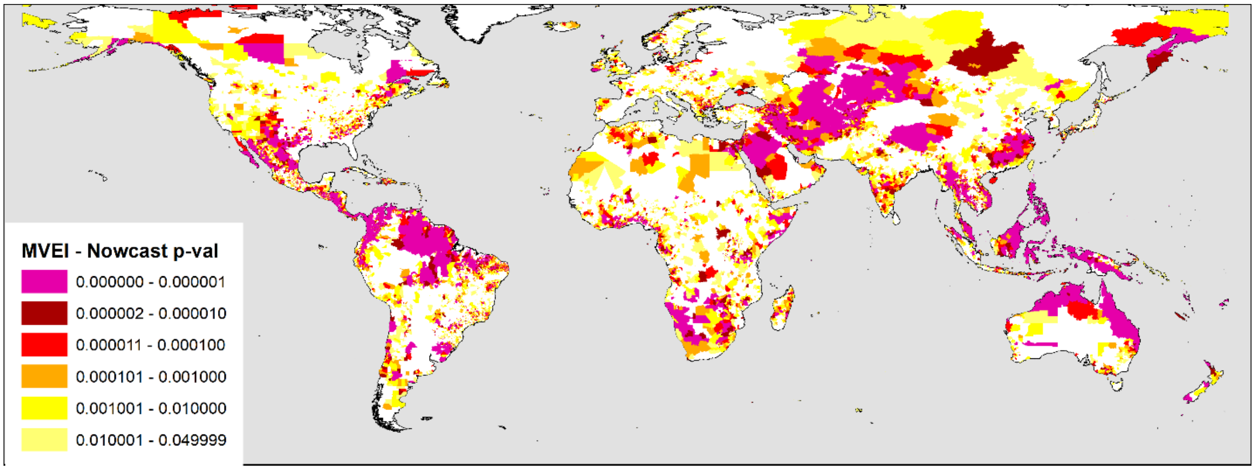

Supplementary Figure 3: C – P-values for relationship between smoothed MVEI and number of nowcasts per month.

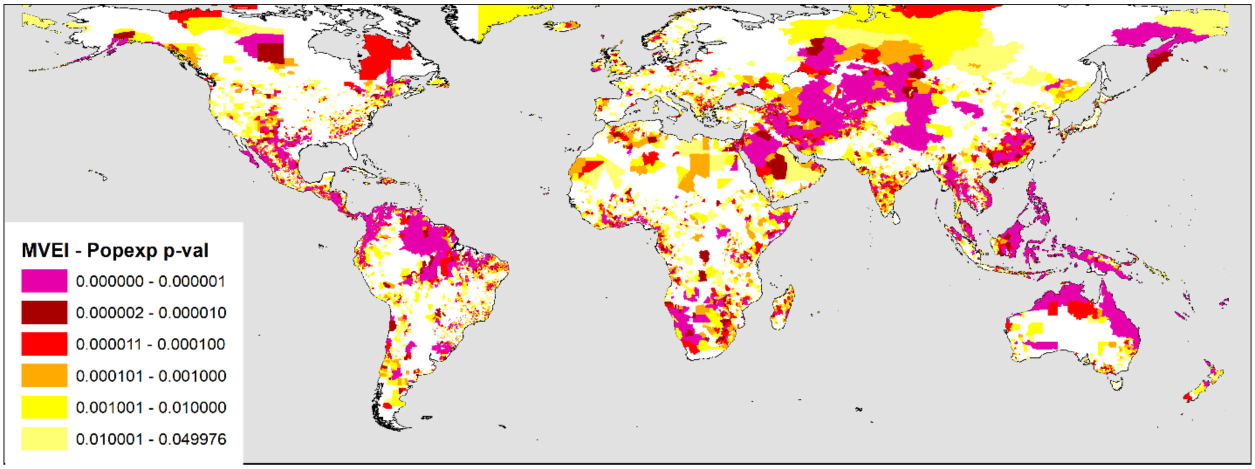

Supplementary Figure 4: D – P-values for relationship between smoothed MVEI and model estimates of population exposure.

The areas where significant relationships are observed (i.e., very low p-values) are broadly consistent for all of the parameters. This demonstrates that in general there are not major changes in the level of significance of the relationship that depend upon each added component in the model.

However, it is important to note that the degree by which ENSO affects each of the parameters can change even while the relationship remains highly significant, as determined by the low p-value. In the next set of figures, we show the slope of the relationship between MVEI and the individual parameters, to demonstrate where the biggest impacts of ENSO are felt for each parameter. For each of these figures, we only show areas where the calculated p-value for the relationship is lower than 0.05.

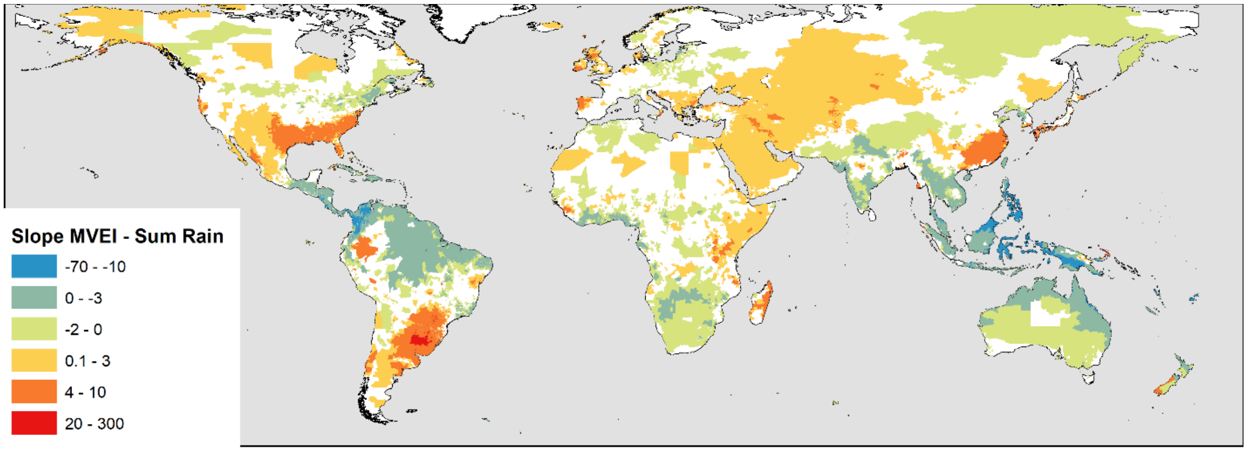

Supplementary Figure 5: Slope of relationship between smoothed MVEI and total rainfall. The values show the change in average monthly rainfall (mm/month) per unit shift in MVEI.

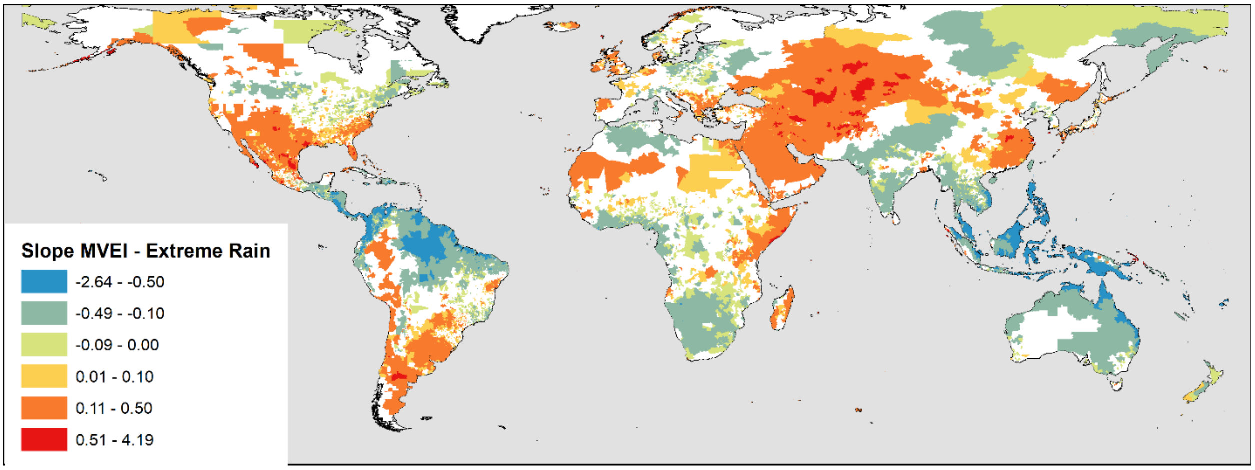

Supplementary Figure 6: Change in extreme rainfall with respect to ENSO. Values show the change in number of days per month where rainfall exceeds historic 95th percentile for a unit change in MVEI. (Red colours indicate areas where El Nino, rather than La Nina, increases number of extreme rainfall days).

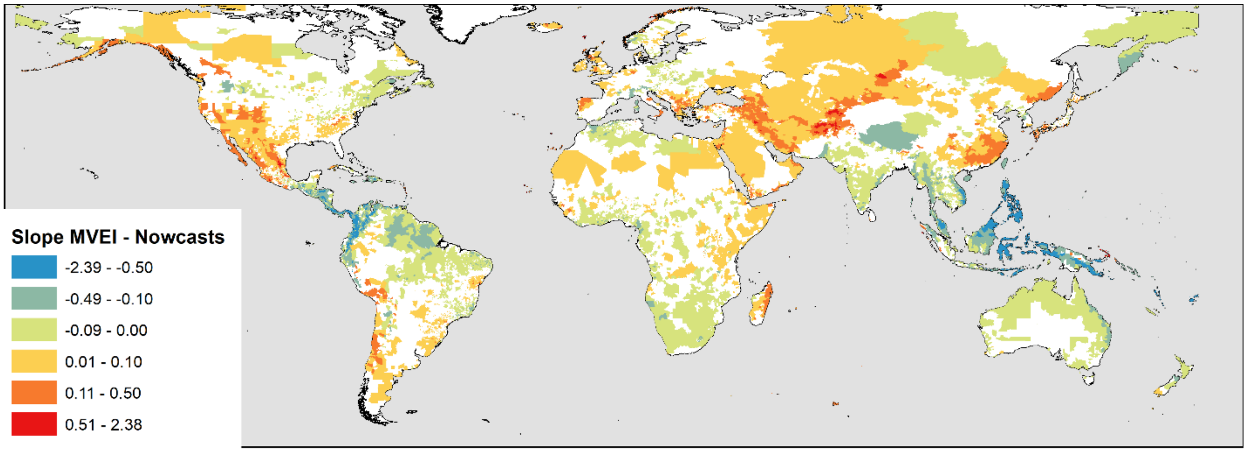

Supplementary Figure 7: Change in hazard nowcasts with respect to ENSO. Values show the change in number of nowcasts per month for a unit change in MVEI. Red colours indicate areas where hazard increases during El Nino conditions.

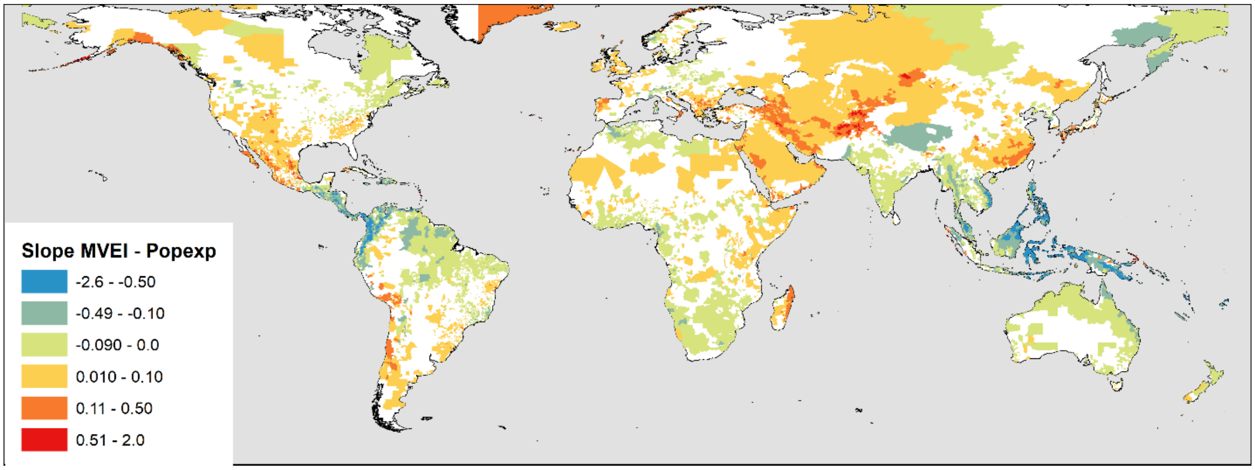

Supplementary Figure 8: Change in population exposure with respect to ENSO. Values show the change in average number of days per month each person in the administrative district in question is exposed to landslide hazard for a unit change in MVEI. Red colours indicate places where El Nino, rather than La Nina, increases exposure.

As can be observed from comparing supplementary figures 6 and 7, while many areas experience large changes in number of days of extreme rainfall with respect to ENSO changes, these changes are much less widespread when considering landslide hazard. However, broadly similar patterns are observed for nowcast changes as for exposure changes, indicating that exposure to hazard is relatively consistent across the world.

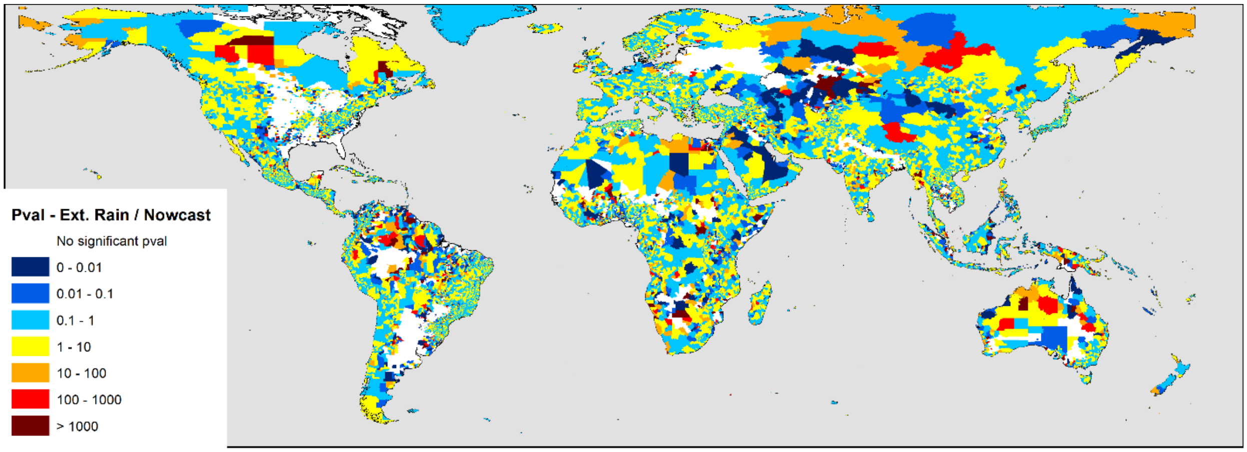

Supplementary Figure 9: Ratio of p-values for Extreme rainfall (Figure S2) and Hazard nowcasts (Figure S3). Red areas indicate locations where the p-values are lower (more significant) for the hazard nowcasts. We observe limited spatial consistency in the ratios.

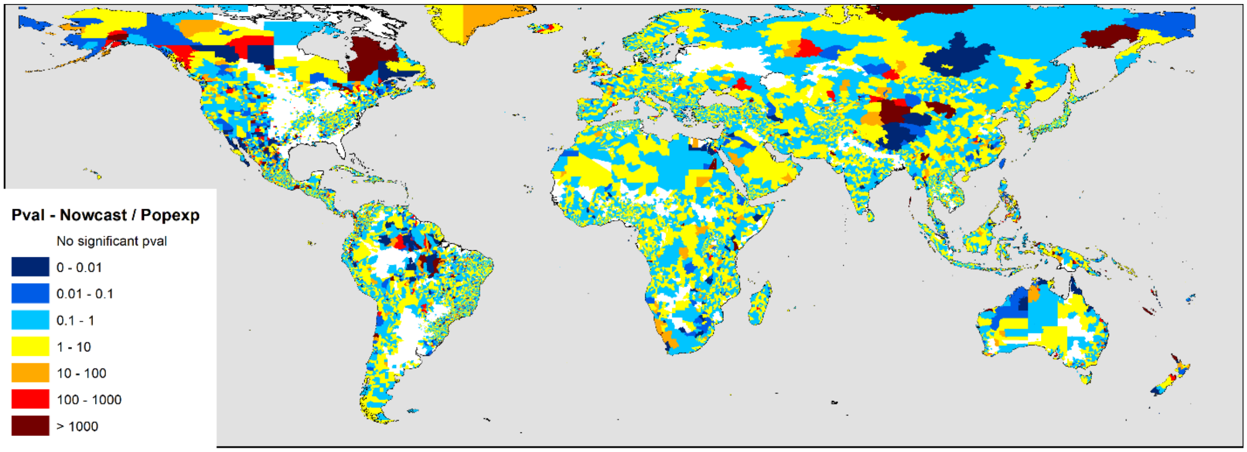

Supplementary Figure 10: Ratio of p-values for Hazard nowcasts (Supplementary figure 3) and Population Exposure (Supplementary figure 4). Red areas indicate regions with a more significant relationship between exposure and MVEI than hazard and MVEI. As in Supplementary figure 9, changes are relatively small and spatially inconsistent, indicating that the major contributor to the significant relationships between exposure and MVEI (Supplementary figures 4 and 8) are driven by the already significant relationships between total rainfall (Supplementary figures 1 and 5) and extreme rainfall (Supplementary figures 2 and 6).

2. Supplementary Tables

Supplemental Table 1: explanation for meaning of p-values in significance calculations.

Parameter	Comment
A) Monthly rainfall	P-value between MVEI and monthly rainfall will show where most significant relationships are

	observed between IMERG rainfall totals and ENSO.
B) Extreme rainfall (number of times per month rainfall exceeds historic 95th percentile)	Our modelling considers rainfall exceeding the historic 95th percentile as the minimum needed to trigger a landslide. P-values will show where ENSO increases / decreases number of days with extreme rainfall.
C) Nowcast (extreme rainfall occurs in an area with moderate or high landslide susceptibility)	By comparing the differences between where ENSO influences extreme rainfall and where ENSO influences Nowcasts, we can see where the effect of ENSO is most pronounced on landslide hazard.
D) Population Exposure	The final model output – population exposure – depends on each of the earlier parameters (extreme rainfall and landslide susceptibility) but also on population. If there are differences between where ENSO influences nowcasts (i.e. landslide hazard) and where it influences population exposure, this is determined by the distribution of population with respect to El Nino changes.

Supplementary Table 2: Monthly exposure values for each admin-2 district around the world.

This table provides the explanation for the fields in the large dataset (Supplementary dataset 2) which contains all of the exposure data used in this study.

Name	Field Definition
Country	Name of country
ISOalpha3	Admin-2 level district name
FID_orig	ID of region
PopulationYYYYMM	Exposure of population for the year (YYYY) and month (MM) listed
RoadsYYYYMM	Exposure of roads for the year (YYYY) and month (MM) listed
Crit_infrYYYMM	Exposure of critical infrastructure (schools, hospitals, fuel stations) for the year (YYYY) and month (MM) listed
Crit_infr_plus_powerYYYYMM	Exposure of critical infrastructure (schools, hospitals, fuel stations) and power distribution and production nodes for the year (YYYY) and month (MM) listed
Nowcast_densityYYYYMM	Number of nowcasts in the district for the year and month listed
Rainfall_exceedenceYYYYMM	Sum of number of days in which Antecedent Rainfall Index exceeds historic 95th percentile for daily rainfall – this is the sum total for all pixels within the admin-2 level district
Raw_rainYYYYMM	Sum of rainfall for month in question. This is the total for all IMERG pixels within the admin-2 level district (0.25 degree resolution)

3. Supplementary Notes

Supplementary Note 1. Significance of relationship between Multi-variate ENSO Index and different aspects of landslide model

The model used to estimate exposure due to landslides depends on rainfall input parameters, as well as a landslide susceptibility model and the GPWv4 global population dataset. While we observe significant relationships between the calculated exposure and El Nino index, as observed by the Multi-variate ENSO index, it is important to determine whether the significance of these relationships is determined primarily as a result of significant relationships between rainfall and ENSO, or whether the significance of the relationship emerges as a result of ENSO changes in areas where there is elevated landslide susceptibility or high population density.

For each of the administrative districts considered, we have calculated the p-value of the relationship between smoothed monthly ENSO index (as estimated by MVEI v2) and monthly values for the following variables which form intermediate steps in our modelling process. These are summarized in supplementary table 1 (above).

Supplementary Note 2: Validation Data – Fatalities in each country split by MVEI value intervals

As described in the main text, we have used the Global Fatal Landslide Database to assess the number of fatalities in each country for different MVEI value intervals, to compare with the distribution of months in each MVEI value interval. This allows us to assess countries where proportionally more landslides occur for a given MVEI interval, to compare with our model outputs. Since we provide figures for each country in which there is a fatality recorded in the database, we do not reproduce the figures below but instead provide them as a single zip file. The generic caption for these figures is shown here:

Top: Histogram of landslide by MVEI value (blue) and MVEI values for the months in the GFLD dataset (2004-2016). Bottom: Ratio of landslides to months (divide blue histogram value by red one) is shown with black points; in red, the number of fatalities occurring at each MVEI interval are divided by the number of months in that interval.
