# Supplementary material for: Global connections between El Nino and landslide impacts
Source: Nat Commun. 2021 Apr 15;12:2262. doi: 10.1038/s41467-021-22398-4 (PMC8050240; doi:10.1038/s41467-021-22398-4)
Supplement: Supplementary file 3 — Description of Additional Supplementary Files [file 41467_2021_22398_MOESM3_ESM.pdf]

Description of Additional Supplementary Files

Supplementary Data 1: Climate Data

This is a zip file of the climate datasets used in this study. This file contains 3 excel tables and a readme text file. The tables contain the MVEI, NAO, and PDO values used to compare with landslide model results.
Each of the three excel spreadsheets has the relevant index for MVEI, PDO, and NAO, based on NOAA climate data. Sources:
MVEI: https://psl.noaa.gov/enso/mei/
PDO: https://www.ncdc.noaa.gov/teleconnections/pdo/
NAO: https://www.ncdc.noaa.gov/teleconnections/nao/
Data format: CSV
Columns: YYYYMM - Year and month
Index value - value of index

Supplementary Data 2: Monthly exposure dataset for global adm-2 districts
This dataset contains the exposure data for population, roads, and critical infrastructure for each month in the analysed period for each admin-2 level district globally. This is a zip file containing a large csv file. There are a large number of fields in the table. Supplementary table 2 explains the fields in this larger dataset.

Supplementary Data 3: Static exposure data for all adm-2 level districts:
This zip file contains a csv file contains the number of elements counted in each adm-2 level district for each exposed element considered: population, km of roads, critical infrastructural elements.

Supplementary Data 4: Standard deviations for ratio between fatality data and model outputs for analysed countries
This zip file containing a csv file that contains the standard deviation for the ratio of recorded fatalities and the population exposure calculated by our model. This value is calculated over all months that the Global Fatal Landslide Dataset (GFLD, Froude and Petley 2018) covers. Lower values indicate a better correspondence between model and observations.

Supplementary Data 5: Combined zip file of figures showing comparison between GFLD and model outputs
This zip file contains the figures for all analysed countries showing the comparison between the GFLD data and modeled population exposure estimates. These correspond to Figures 7 and 8 in the main text.
The generic caption for these figures is as follows: for country X

Figure 8. Top (A): Histogram of average fatal landslide events in X split by MVEI index are shown in red; in blue, a histogram of the average modelled landslide exposure split by MVEI index. In the lower figure (B), the relative ratio of these two values is shown. A value of 1 indicates that the model perfectly matches the fatality data relative to the maxima of each; lower values indicate that the model over-predicts fatalities.
