# Supplementary figures and images for: Global connections between El Nino and landslide impacts
Source: Nat Commun. 2021 Apr 15;12:2262. doi: 10.1038/s41467-021-22398-4 (PMC8050240; doi:10.1038/s41467-021-22398-4)

Fatal landslides (GFLD) vs modeled exposure for Afghanistan, n= 22

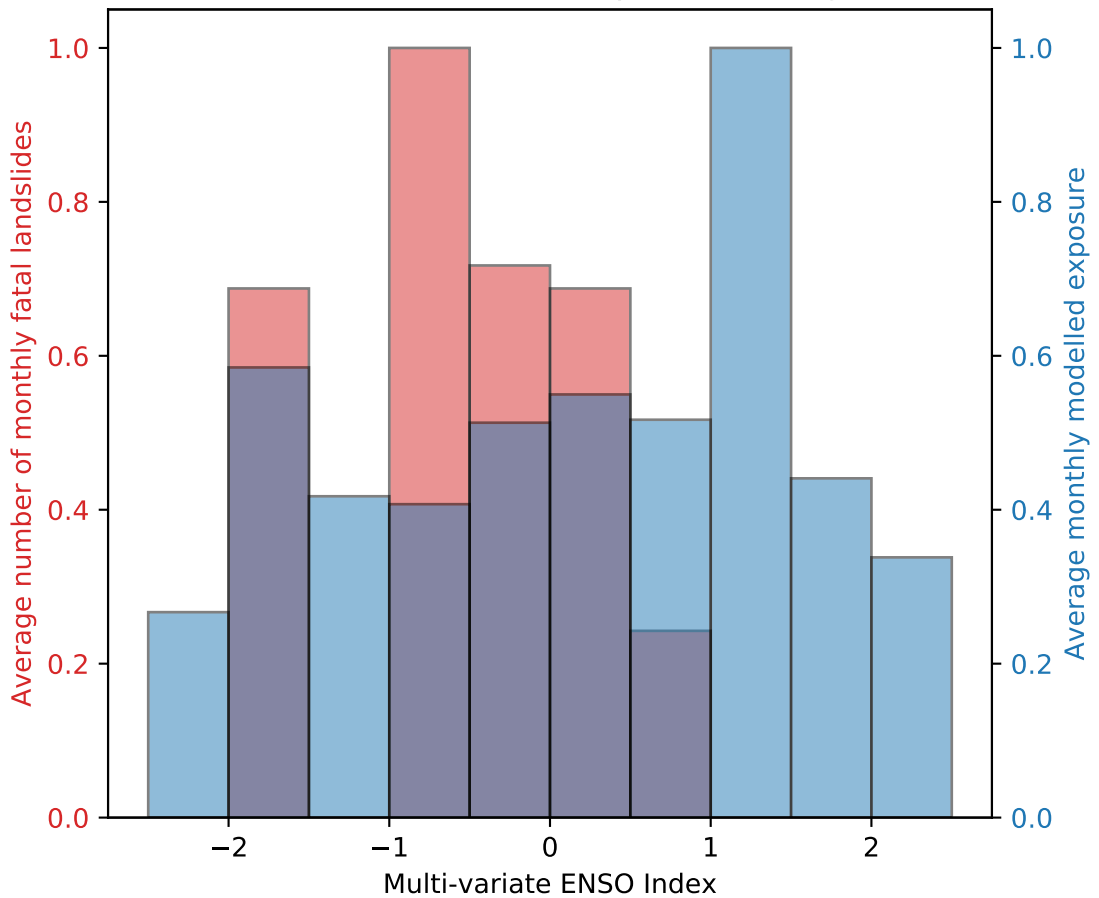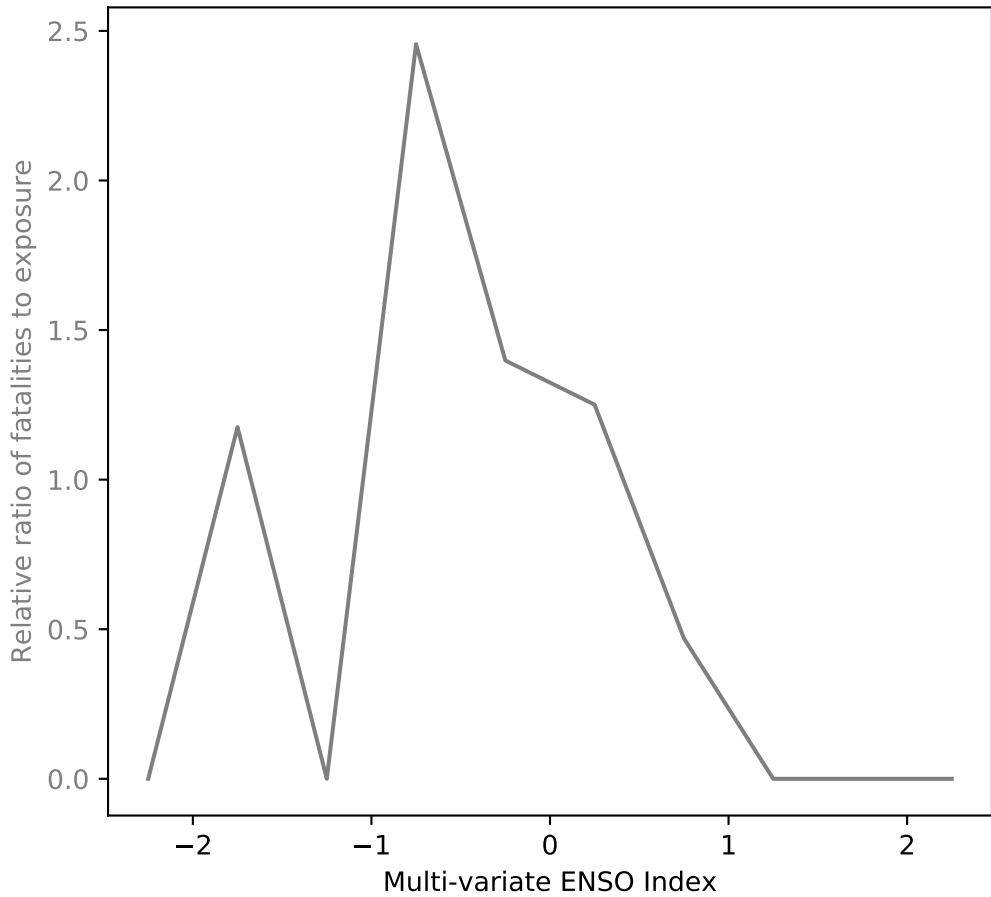

Supplement: Supplementary file 8 — Supplementary Data 5 [file 41467_2021_22398_MOESM8_ESM.zip › Afghanistan_landslide_incidence_compare.pdf]

Fatal landslides (GFLD) vs modeled exposure for Bangladesh, n= 153

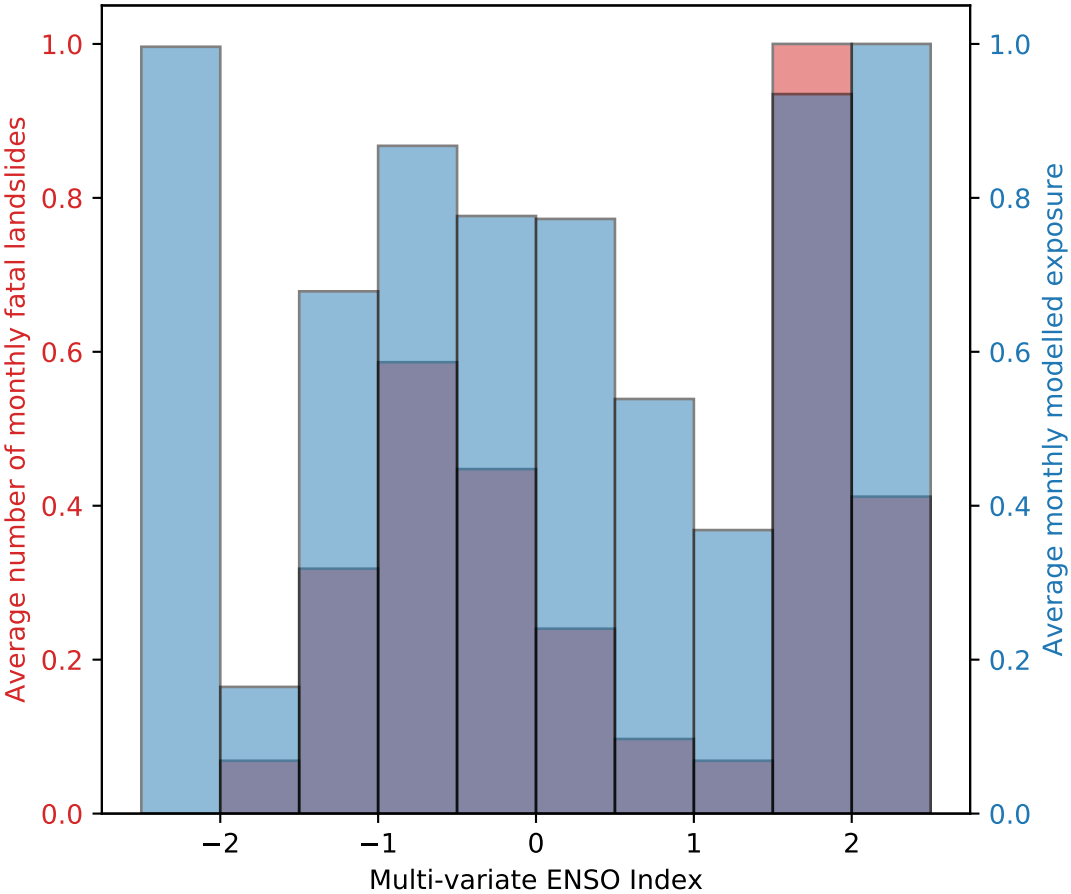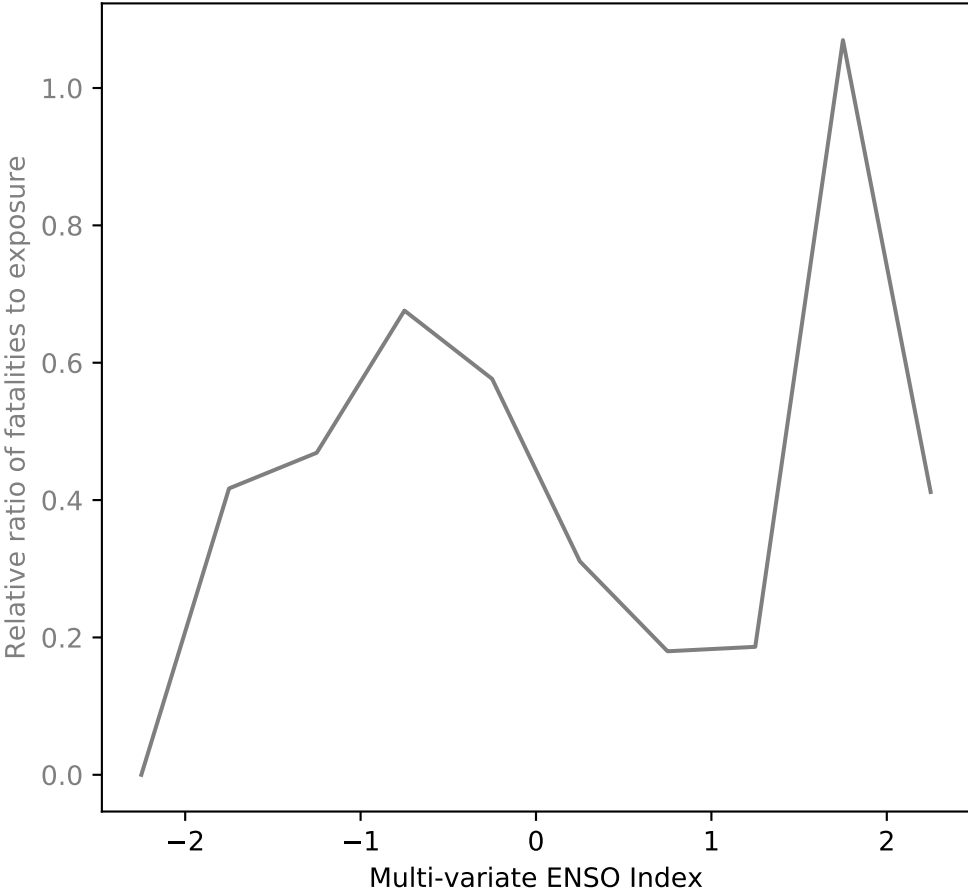

Supplement: Supplementary file 8 — Supplementary Data 5 [file 41467_2021_22398_MOESM8_ESM.zip › Bangladesh_landslide_incidence_compare.pdf]

Fatal landslides (GFLD) vs modeled exposure for Bhutan, n= 30

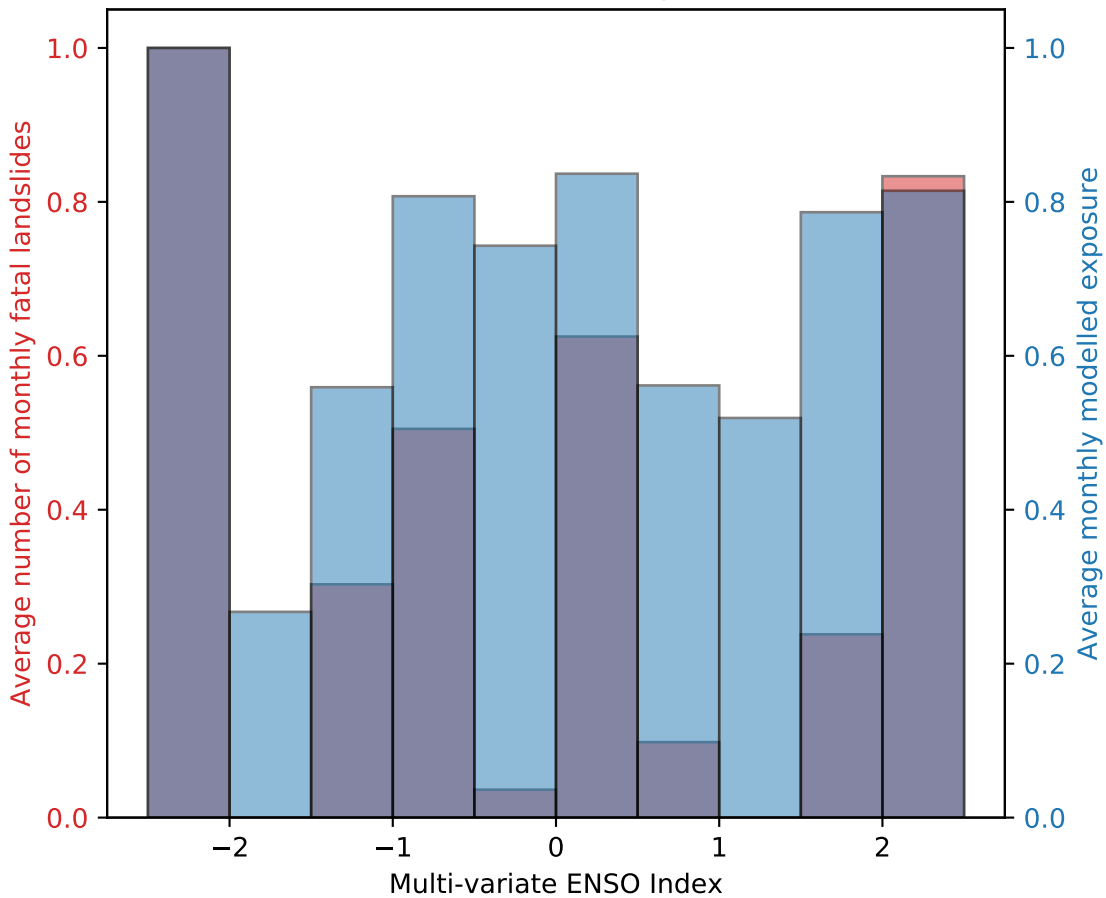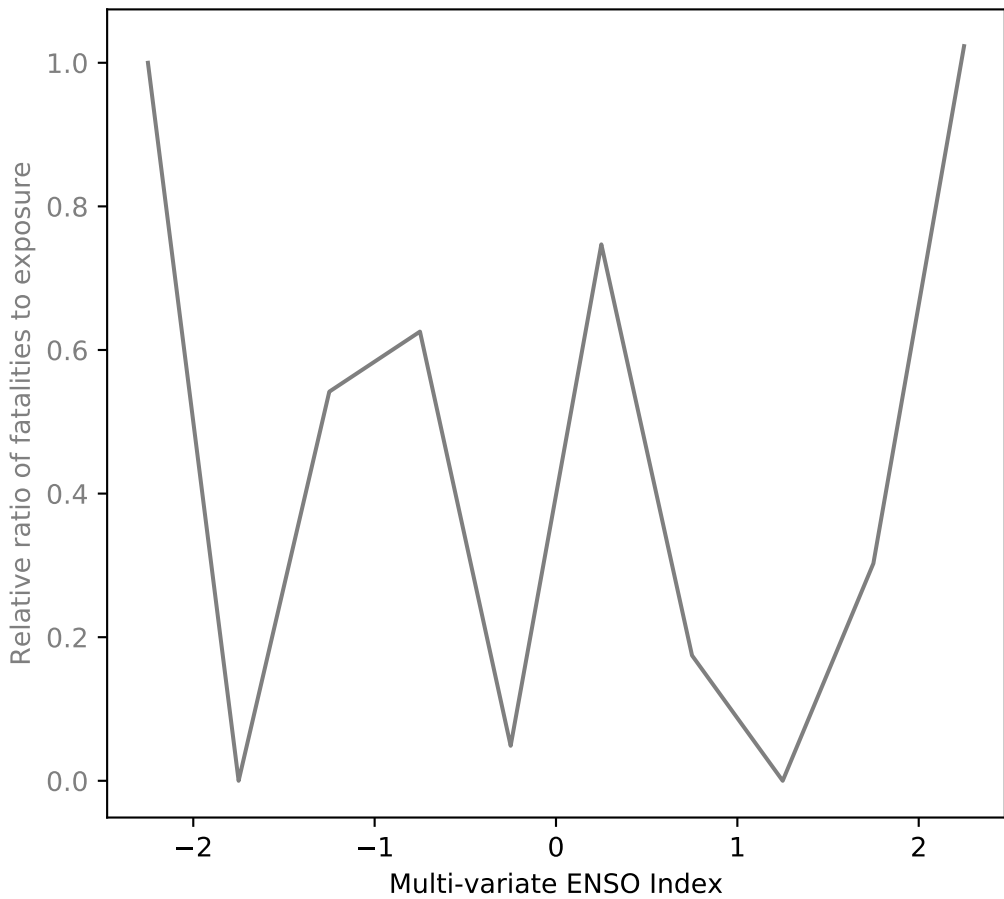

Supplement: Supplementary file 8 — Supplementary Data 5 [file 41467_2021_22398_MOESM8_ESM.zip › Bhutan_landslide_incidence_compare.pdf]

Fatal landslides (GFLD) vs modeled exposure for Brazil, n= 136

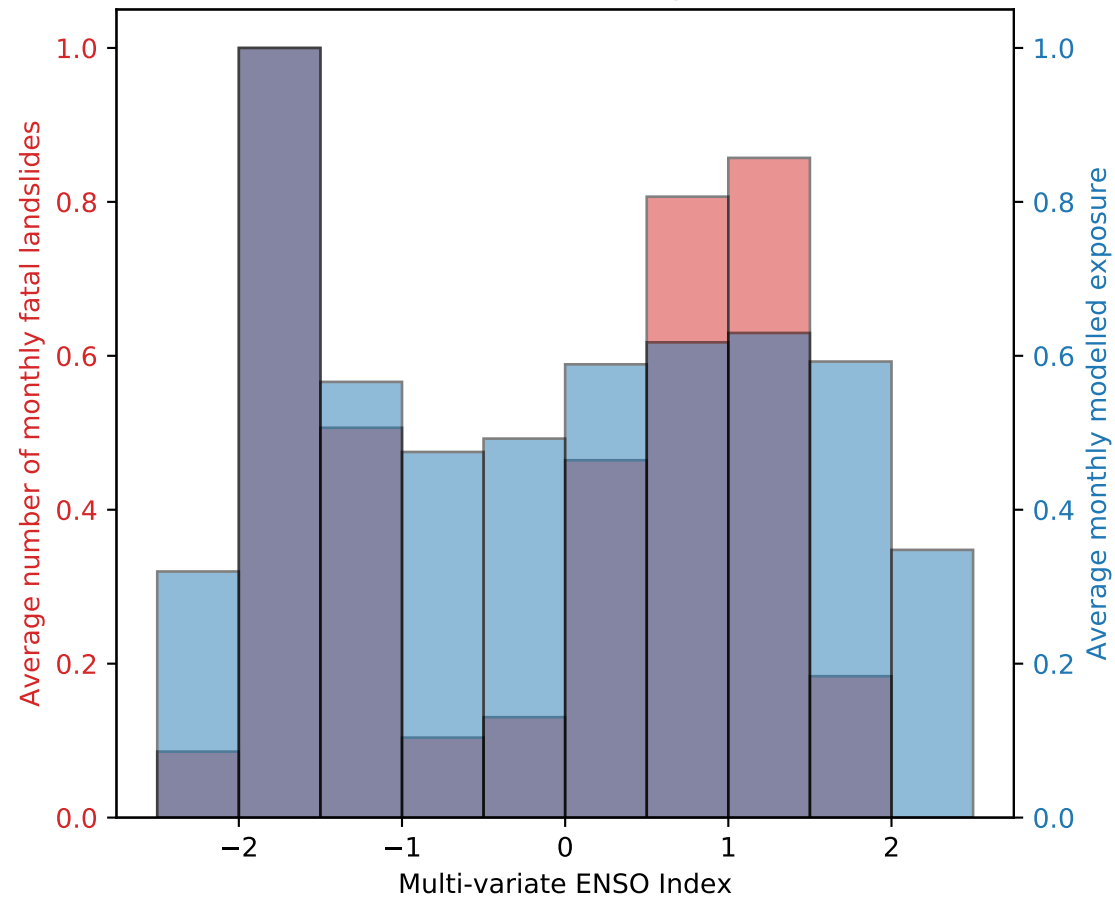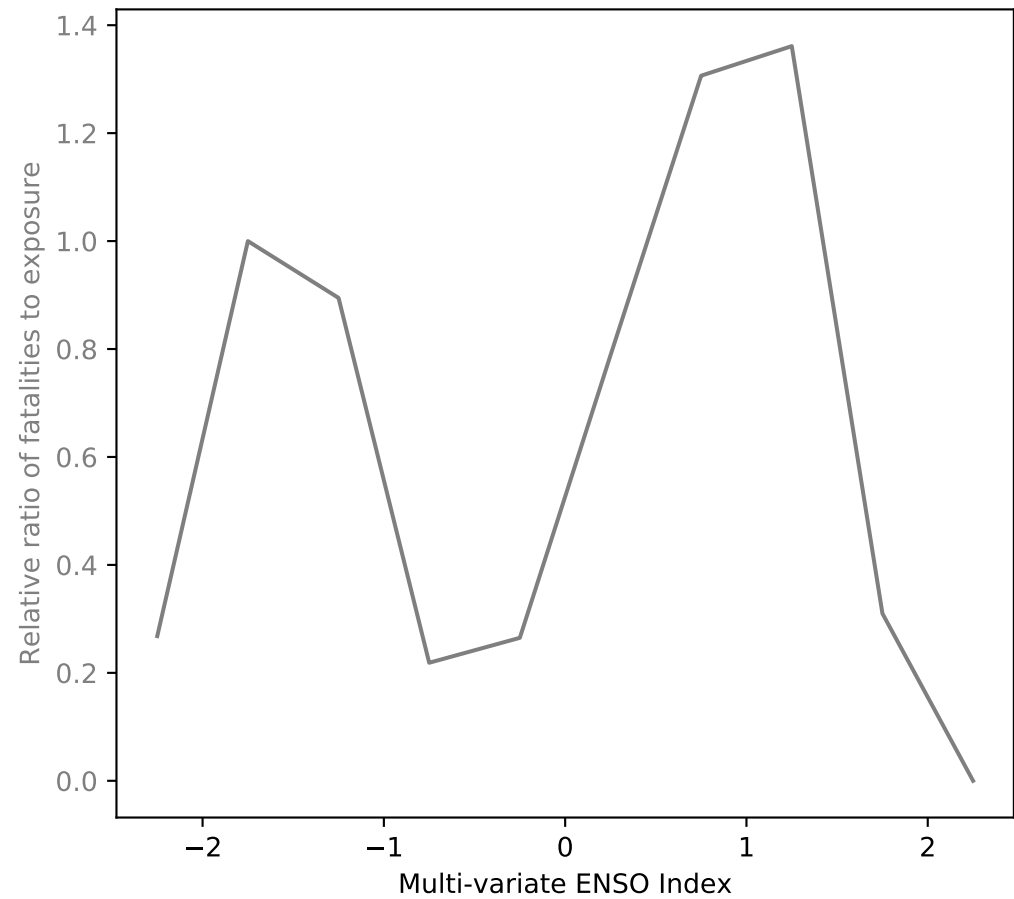

Supplement: Supplementary file 8 — Supplementary Data 5 [file 41467_2021_22398_MOESM8_ESM.zip › Brazil_landslide_incidence_compare.pdf]

Fatal landslides (GFLD) vs modeled exposure for Chile, n= 22

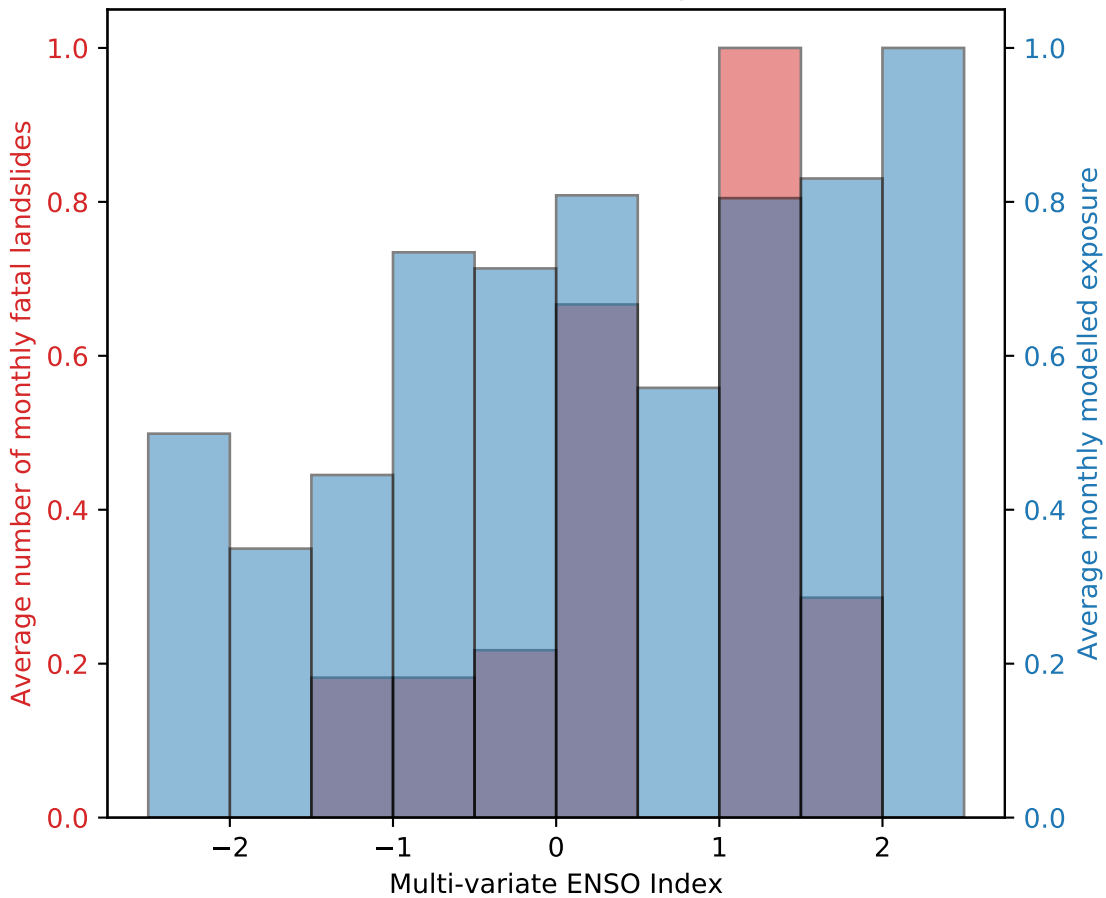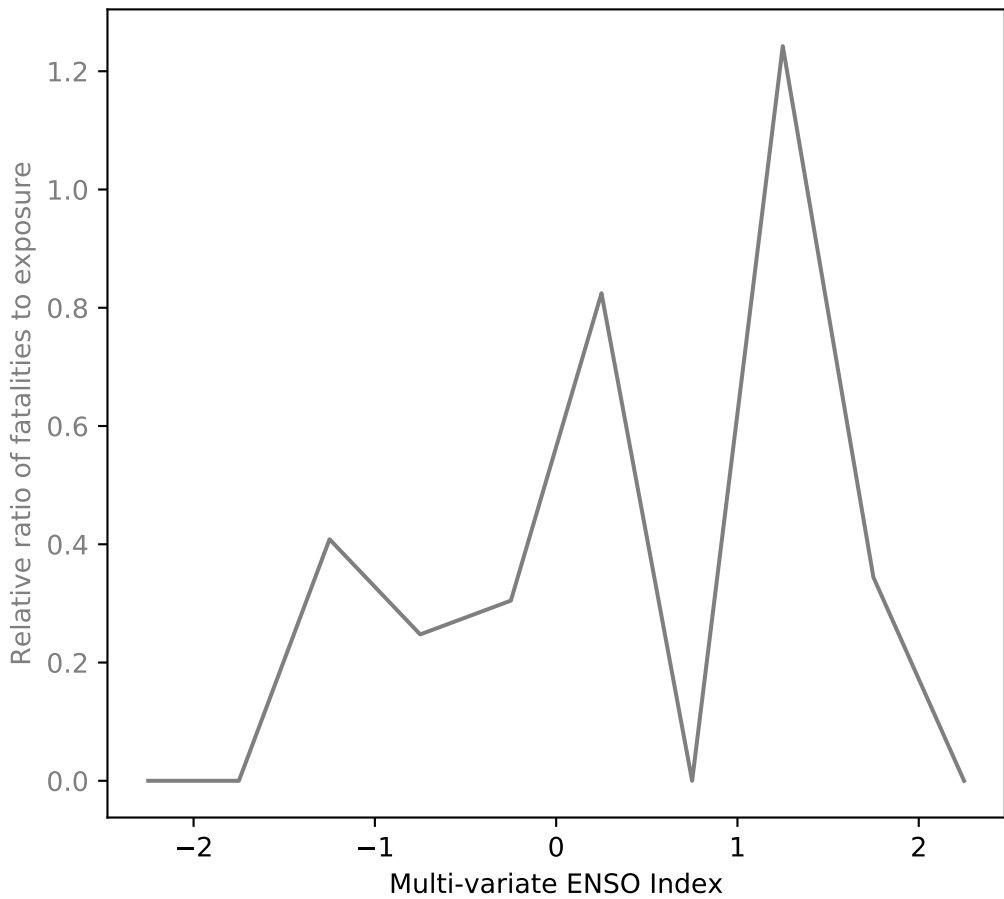

Supplement: Supplementary file 8 — Supplementary Data 5 [file 41467_2021_22398_MOESM8_ESM.zip › Chile_landslide_incidence_compare.pdf]

Fatal landslides (GFLD) vs modeled exposure for China, n= 647

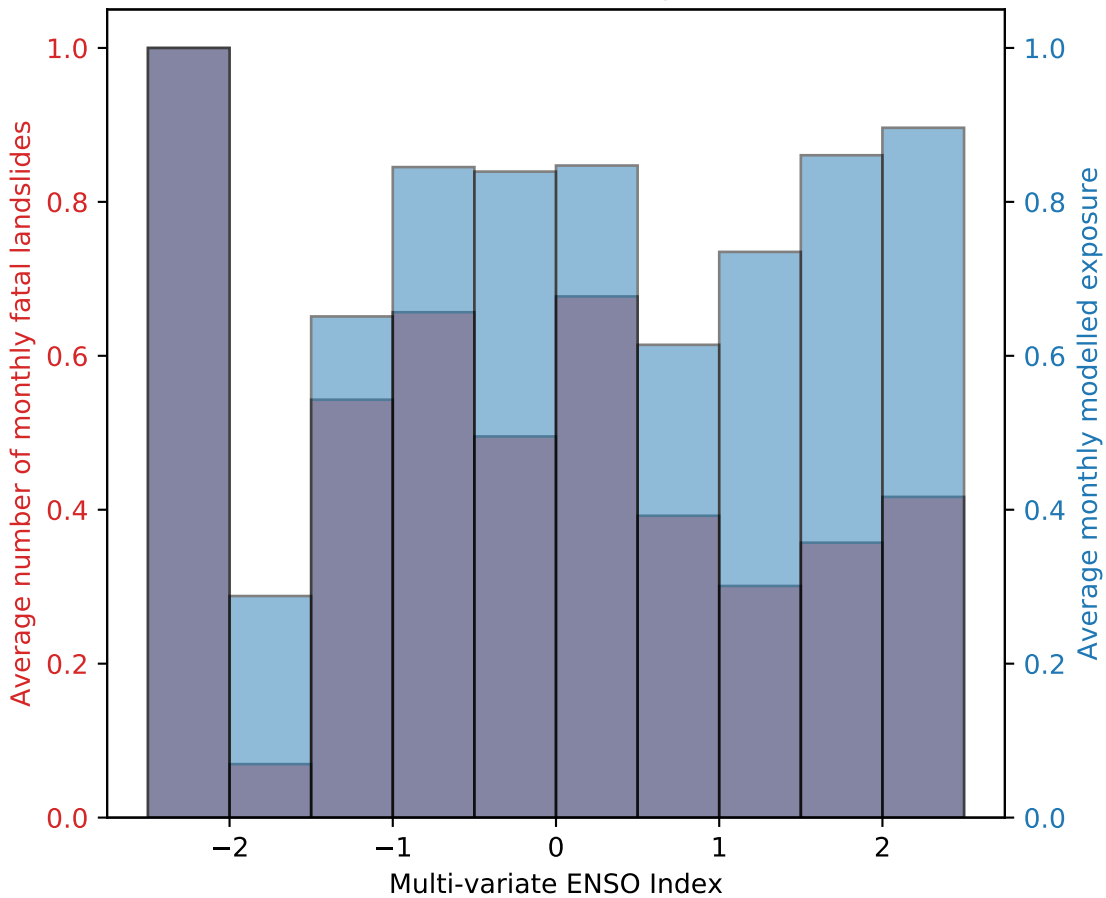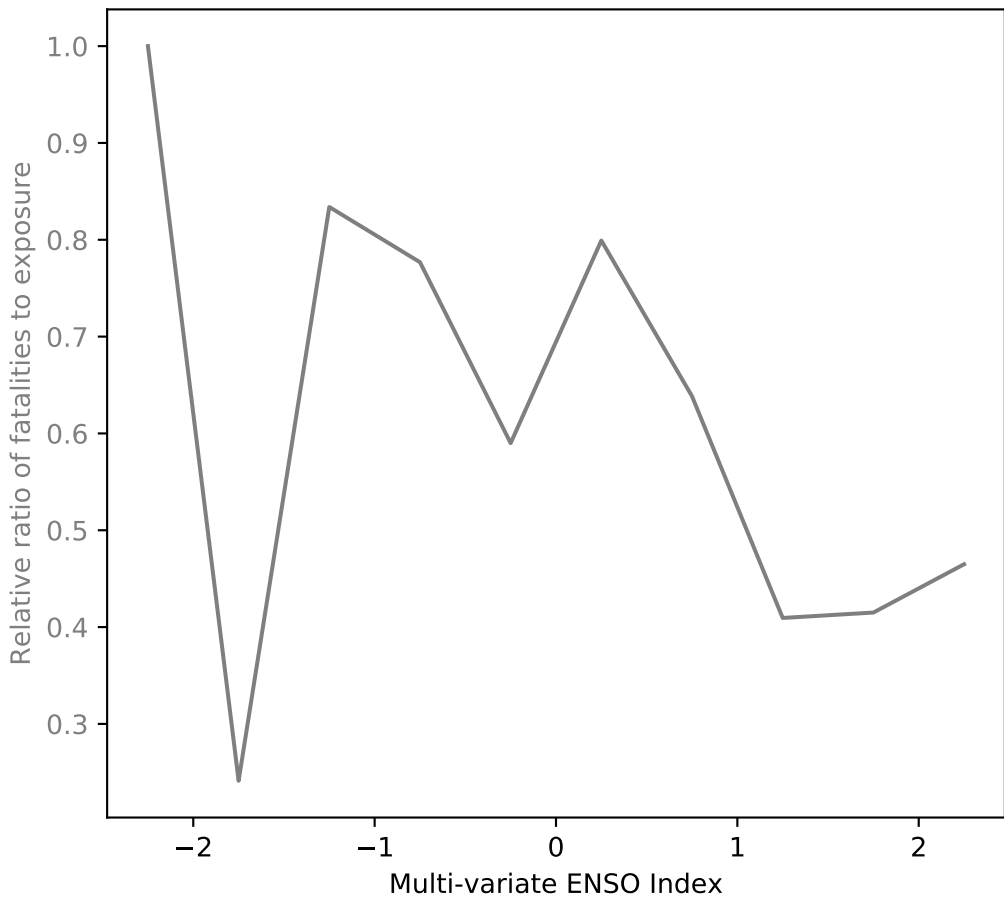

Supplement: Supplementary file 8 — Supplementary Data 5 [file 41467_2021_22398_MOESM8_ESM.zip › China_landslide_incidence_compare.pdf]

Fatal landslides (GFLD) vs modeled exposure for Colombia, n= 129

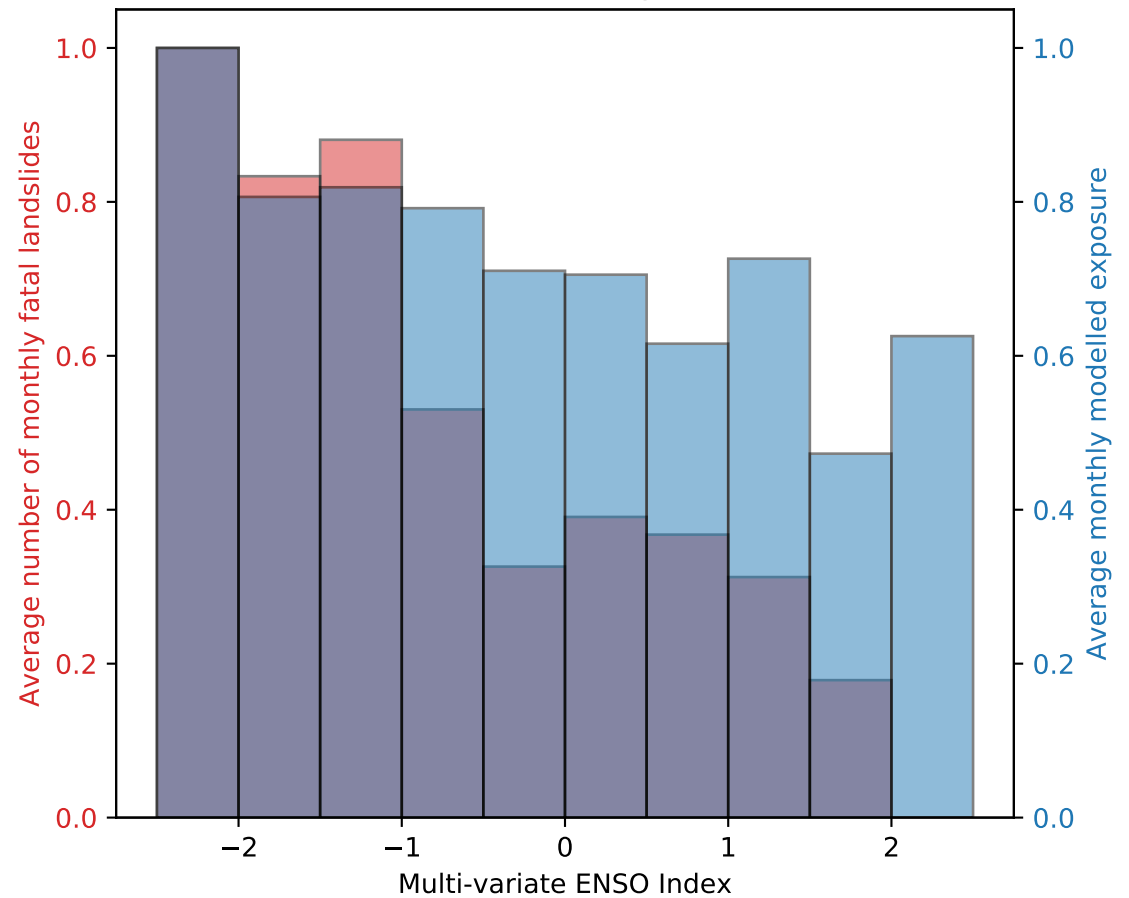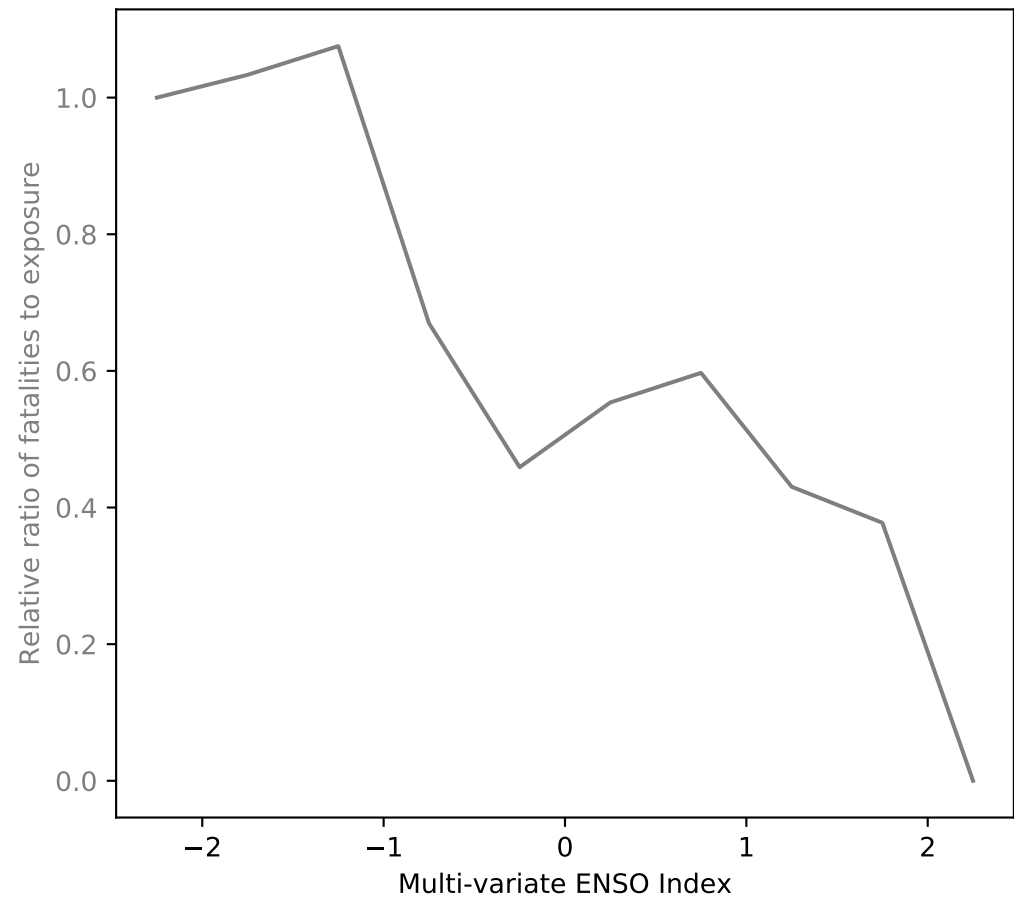

Supplement: Supplementary file 8 — Supplementary Data 5 [file 41467_2021_22398_MOESM8_ESM.zip › Colombia_landslide_incidence_compare.pdf]

Fatal landslides (GFLD) vs modeled exposure for Costa Rica, n= 21

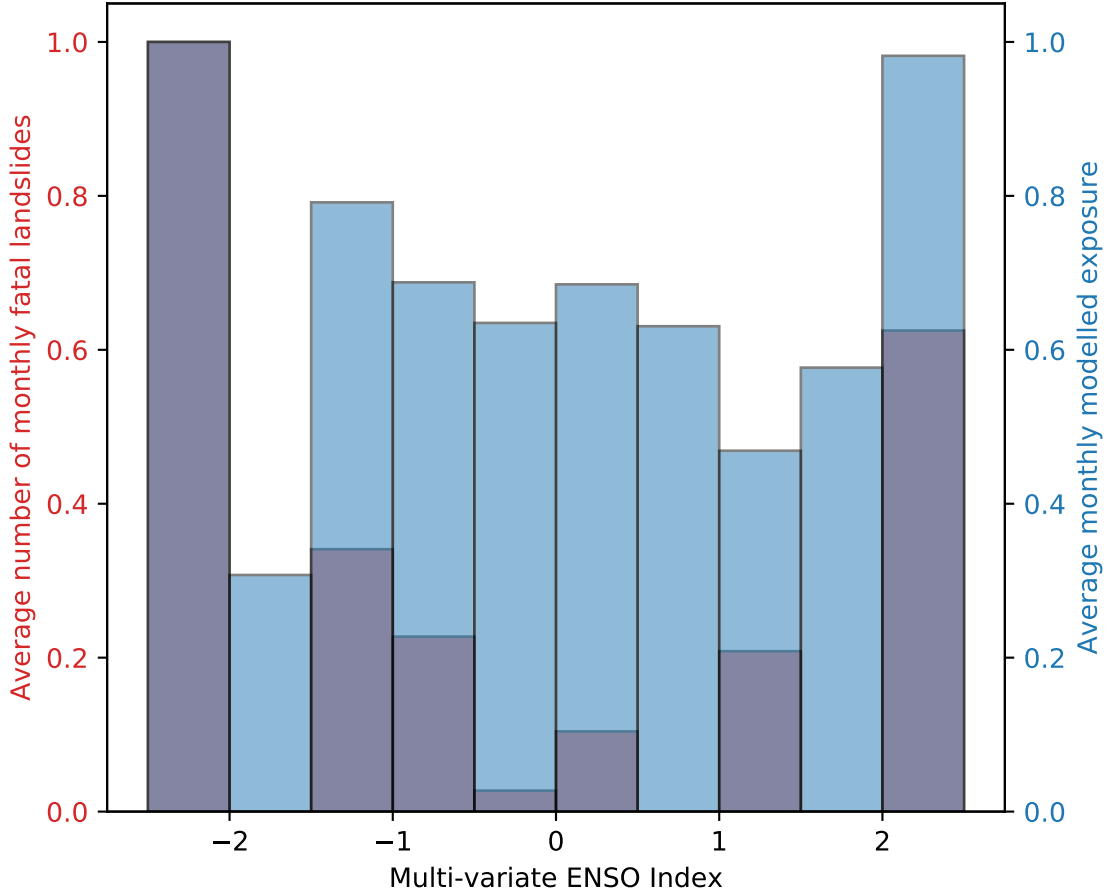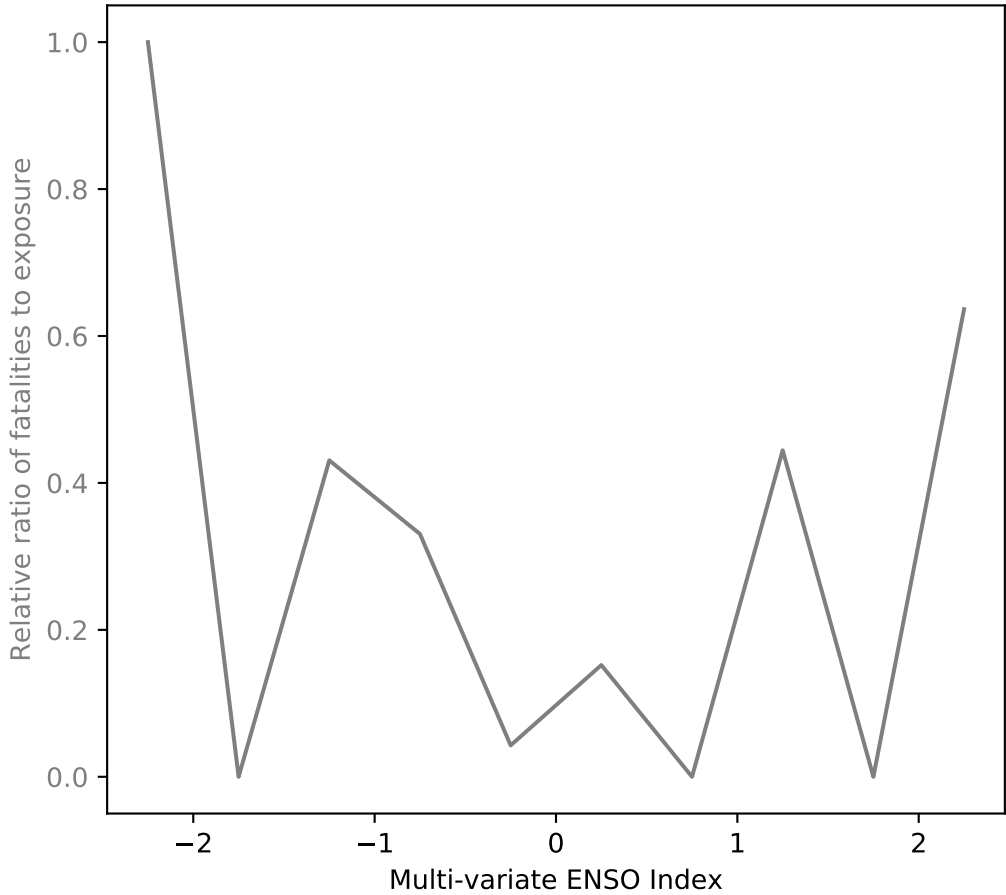

Supplement: Supplementary file 8 — Supplementary Data 5 [file 41467_2021_22398_MOESM8_ESM.zip › Costa Rica_landslide_incidence_compare.pdf]

Fatal landslides (GFLD) vs modeled exposure for Ecuador, n= 21

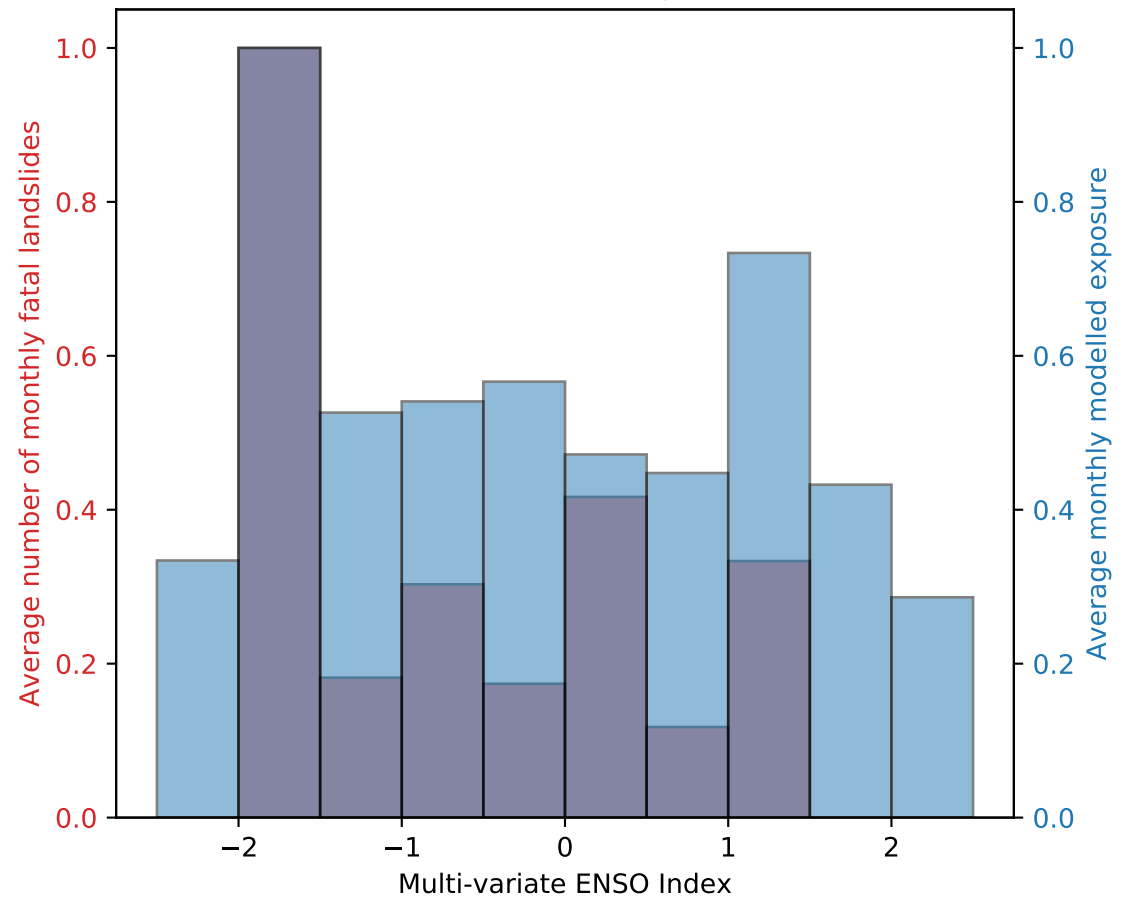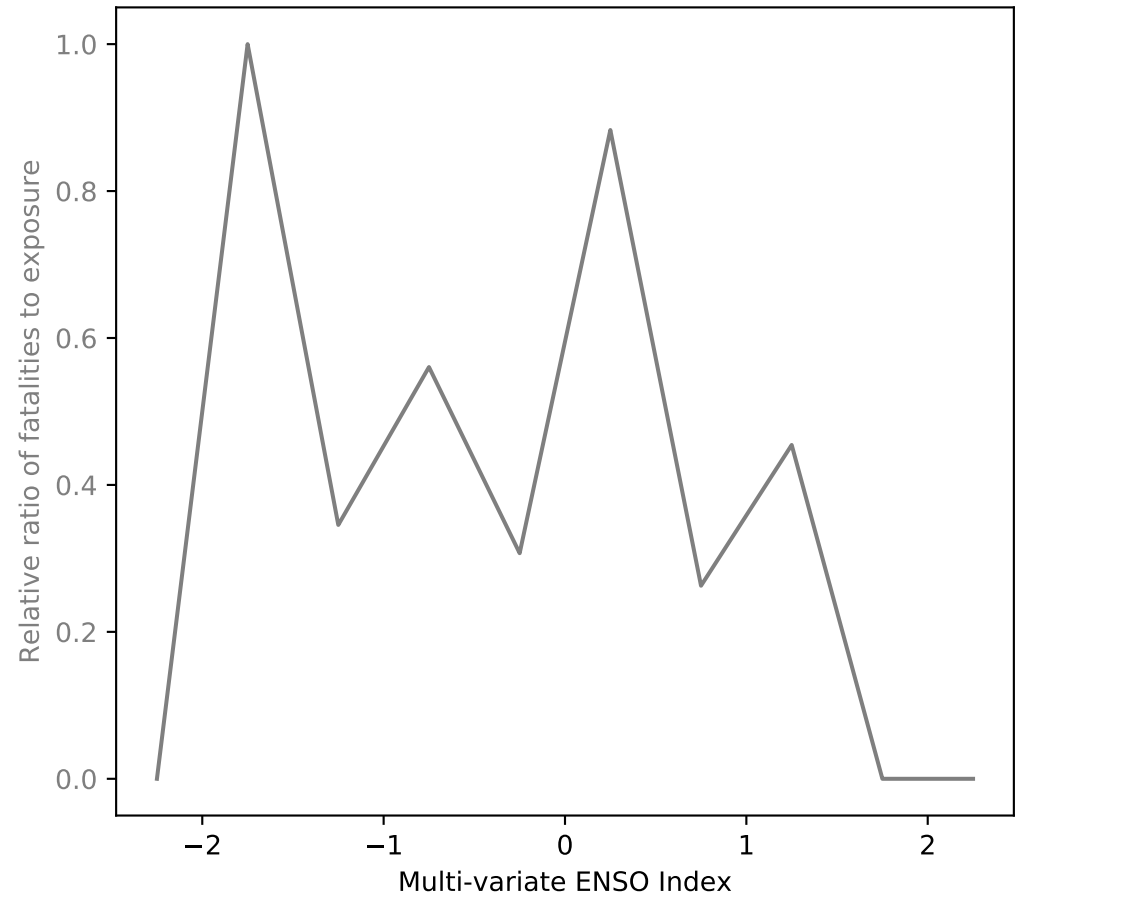

Supplement: Supplementary file 8 — Supplementary Data 5 [file 41467_2021_22398_MOESM8_ESM.zip › Ecuador_landslide_incidence_compare.pdf]

Fatal landslides (GFLD) vs modeled exposure for El Salvador, n= 22

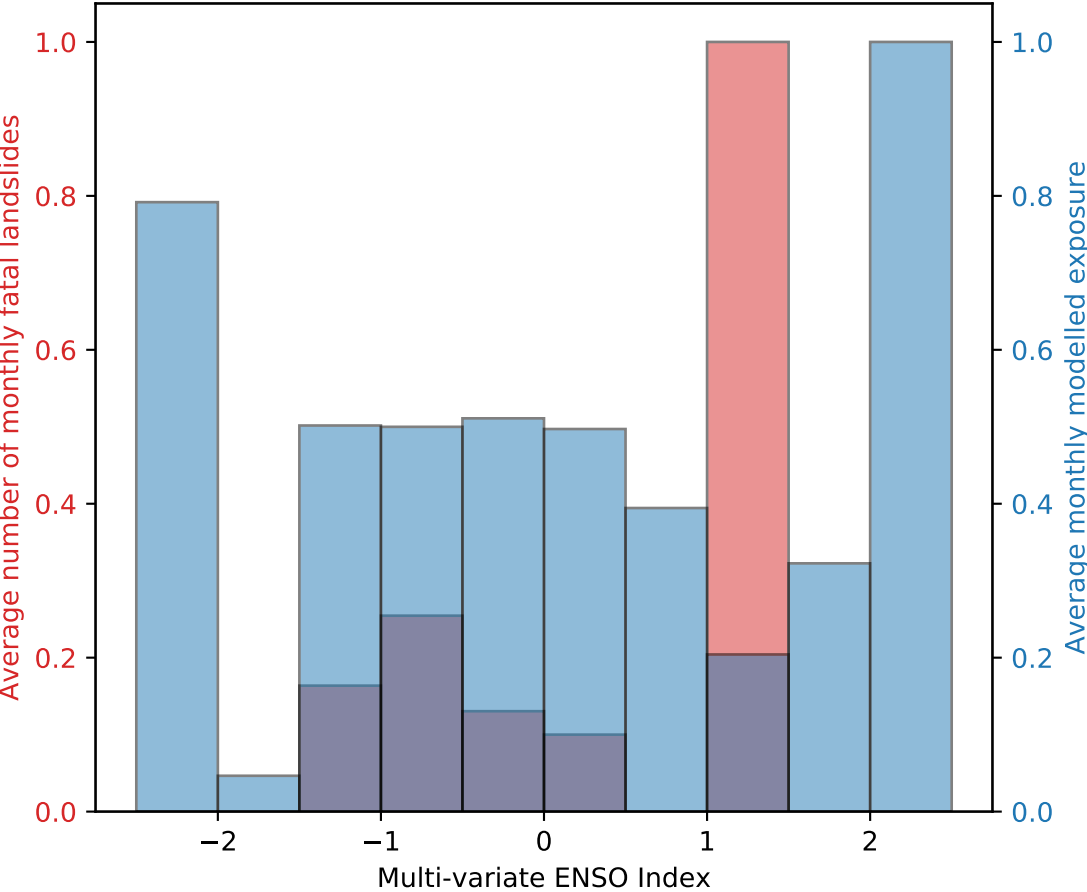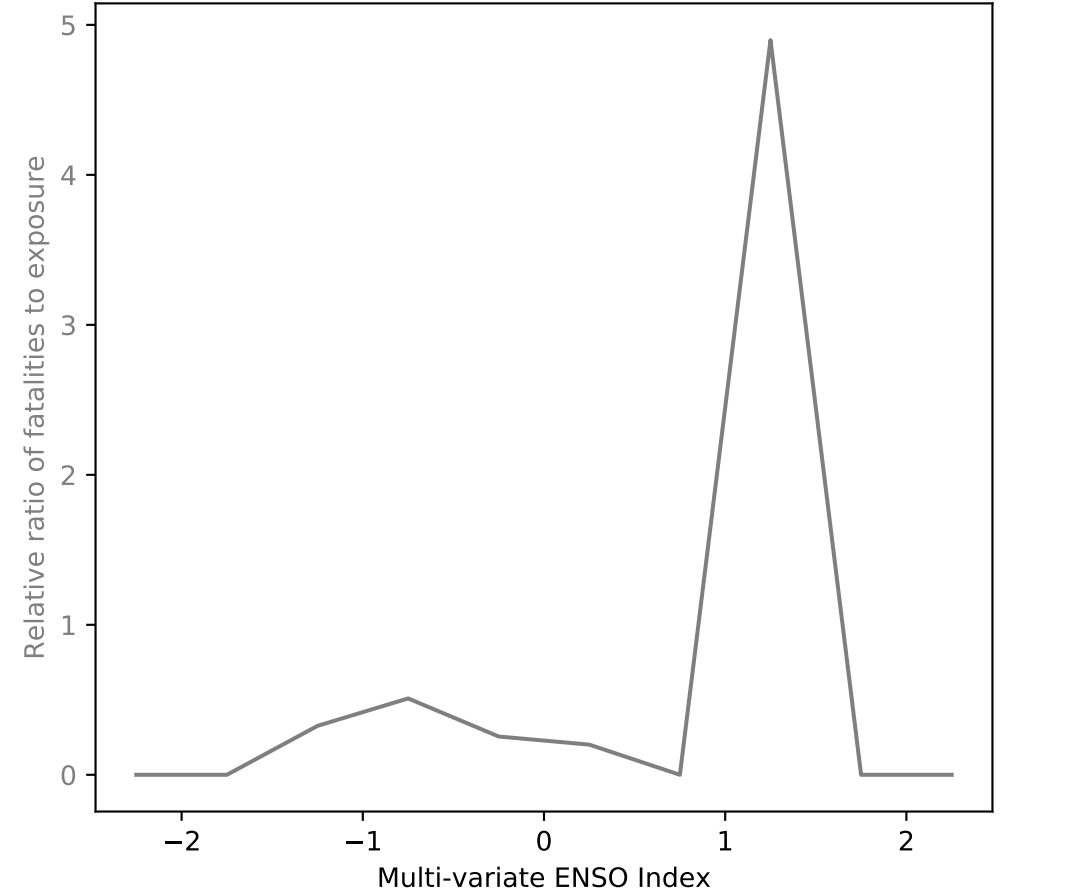

Supplement: Supplementary file 8 — Supplementary Data 5 [file 41467_2021_22398_MOESM8_ESM.zip › El Salvador_landslide_incidence_compare.pdf]

Fatal landslides (GFLD) vs modeled exposure for Guatemala, n= 70

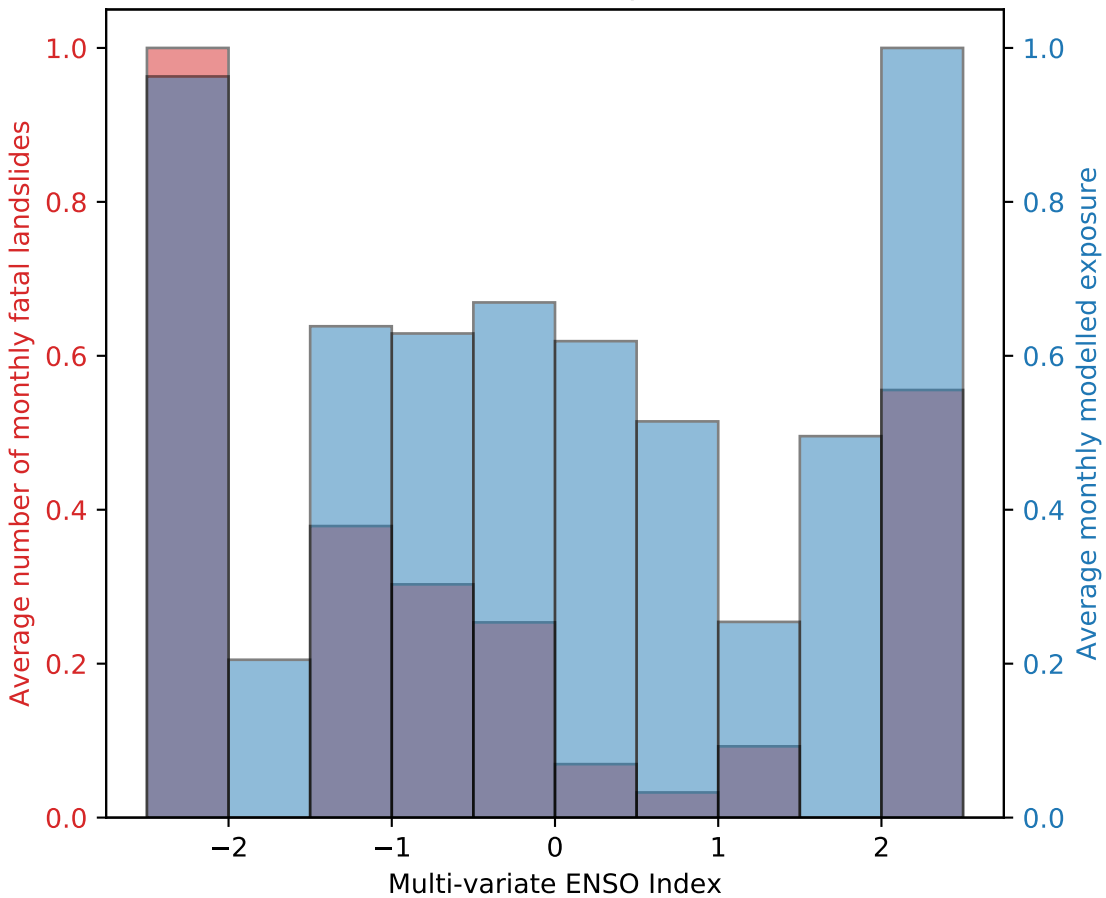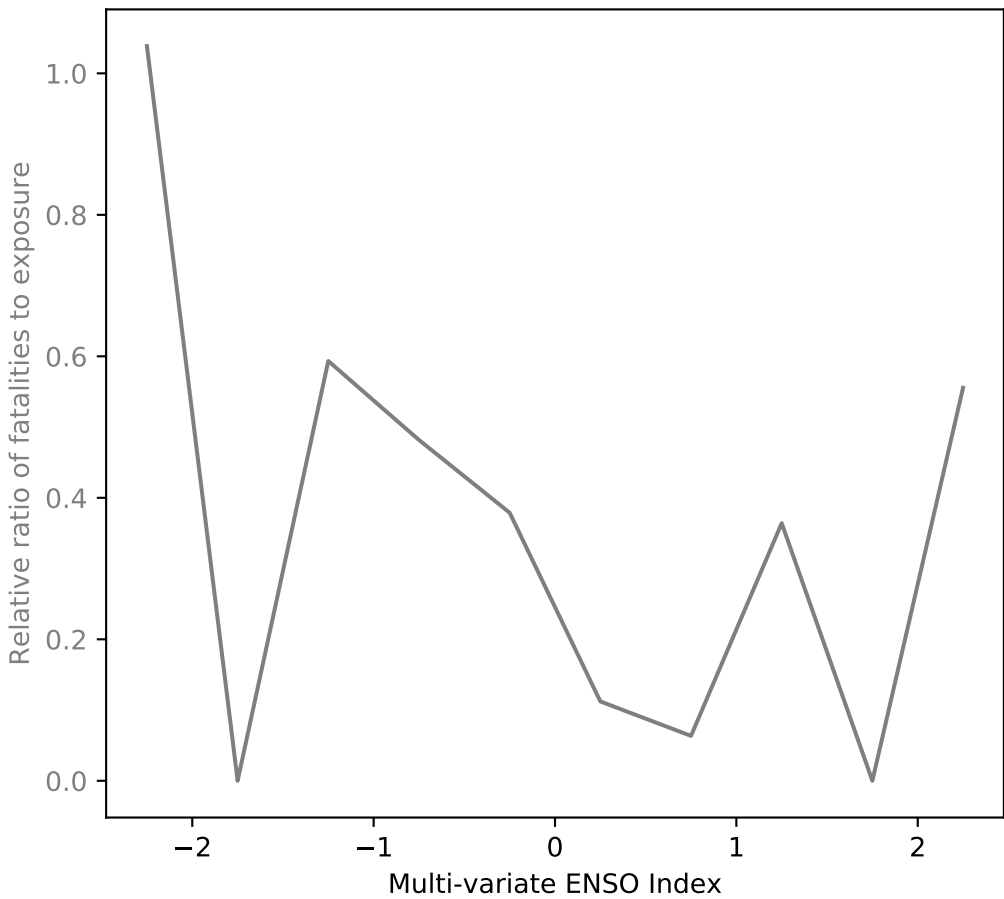

Supplement: Supplementary file 8 — Supplementary Data 5 [file 41467_2021_22398_MOESM8_ESM.zip › Guatemala_landslide_incidence_compare.pdf]

Fatal landslides (GFLD) vs modeled exposure for Haiti, n= 36

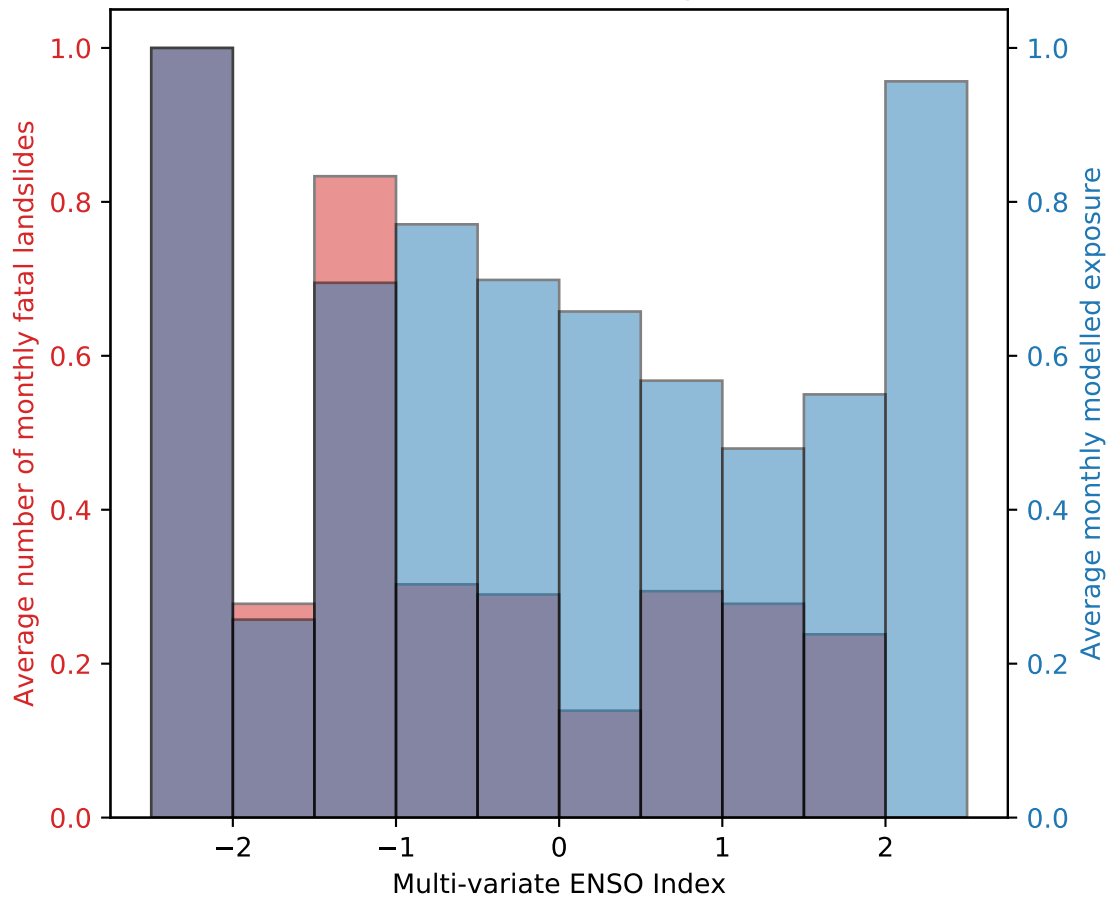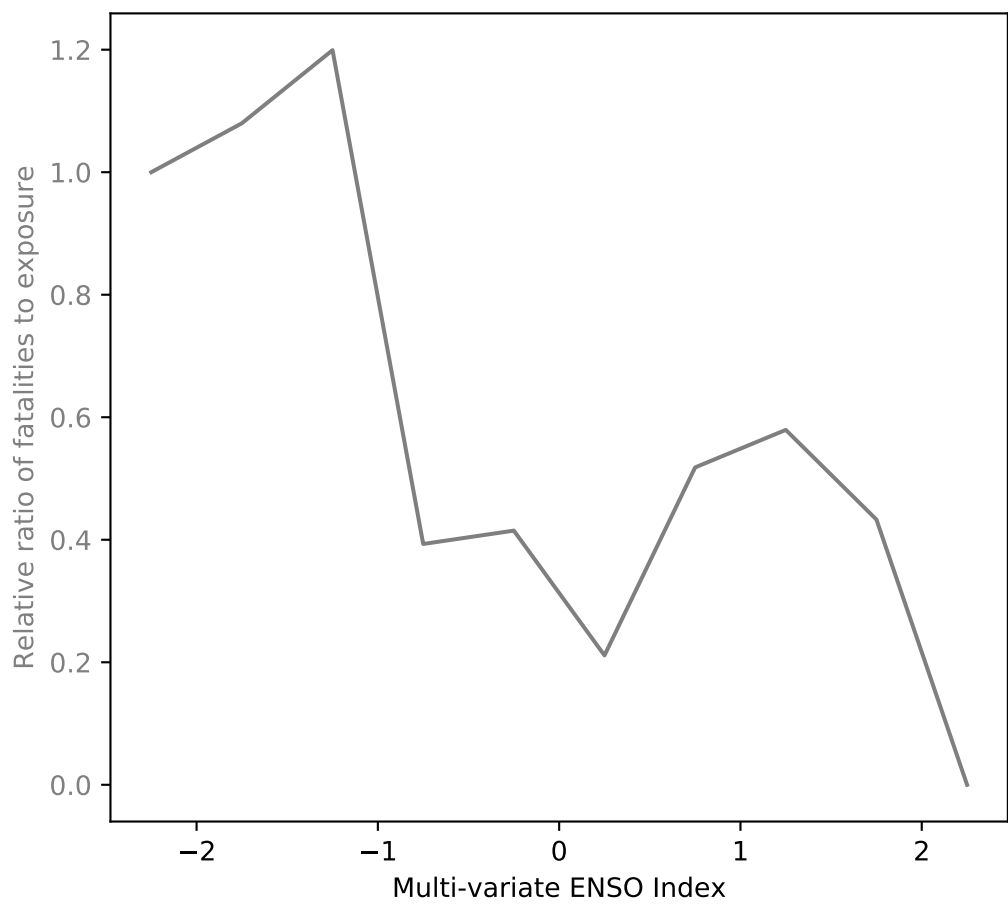

Supplement: Supplementary file 8 — Supplementary Data 5 [file 41467_2021_22398_MOESM8_ESM.zip › Haiti_landslide_incidence_compare.pdf]

Fatal landslides (GFLD) vs modeled exposure for India, n= 923

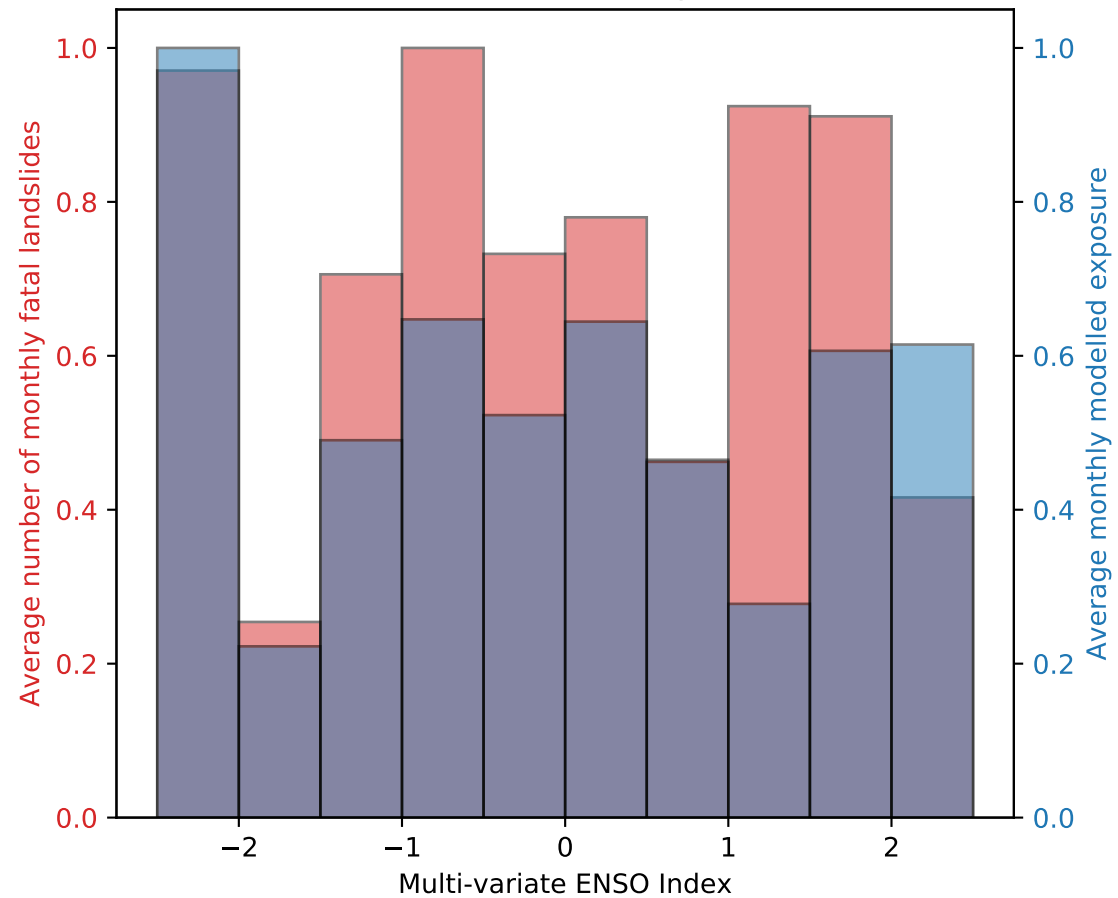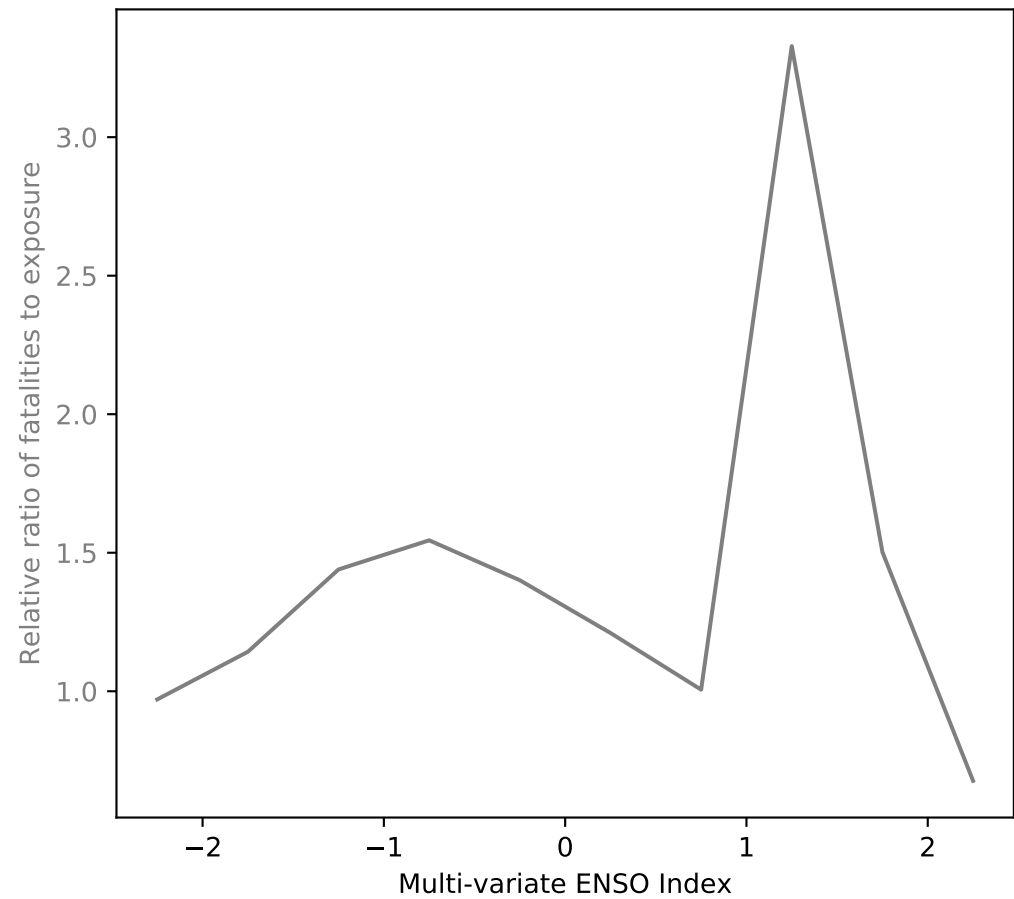

Supplement: Supplementary file 8 — Supplementary Data 5 [file 41467_2021_22398_MOESM8_ESM.zip › India_landslide_incidence_compare.pdf]

Fatal landslides (GFLD) vs modeled exposure for Indonesia, n= 352

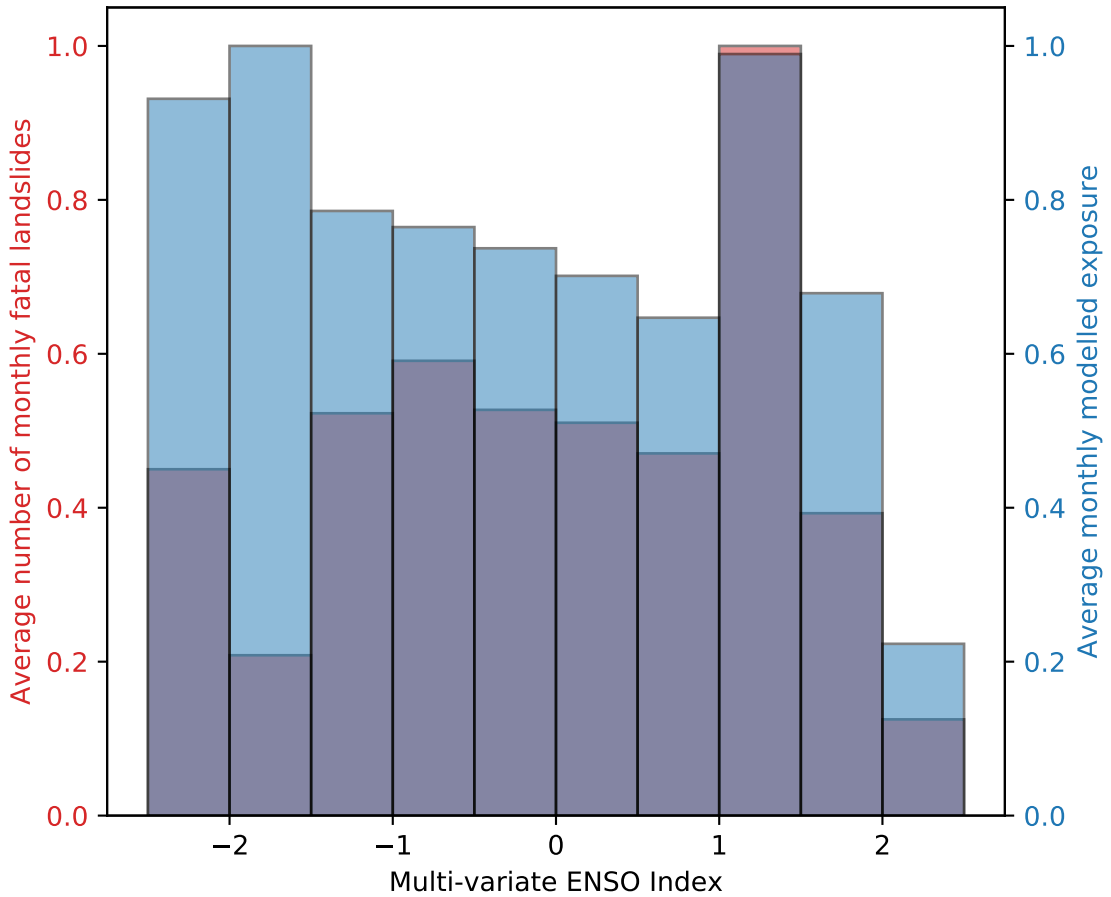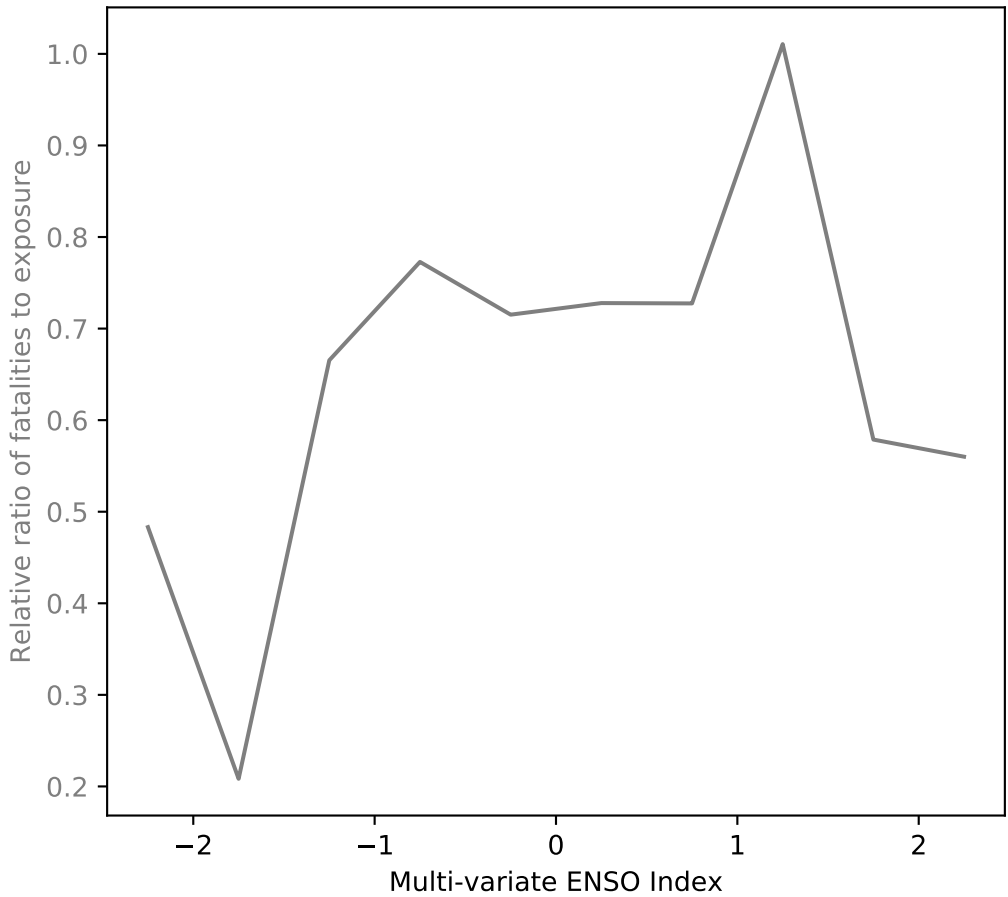

Supplement: Supplementary file 8 — Supplementary Data 5 [file 41467_2021_22398_MOESM8_ESM.zip › Indonesia_landslide_incidence_compare.pdf]

Fatal landslides (GFLD) vs modeled exposure for Kenya, n= 63

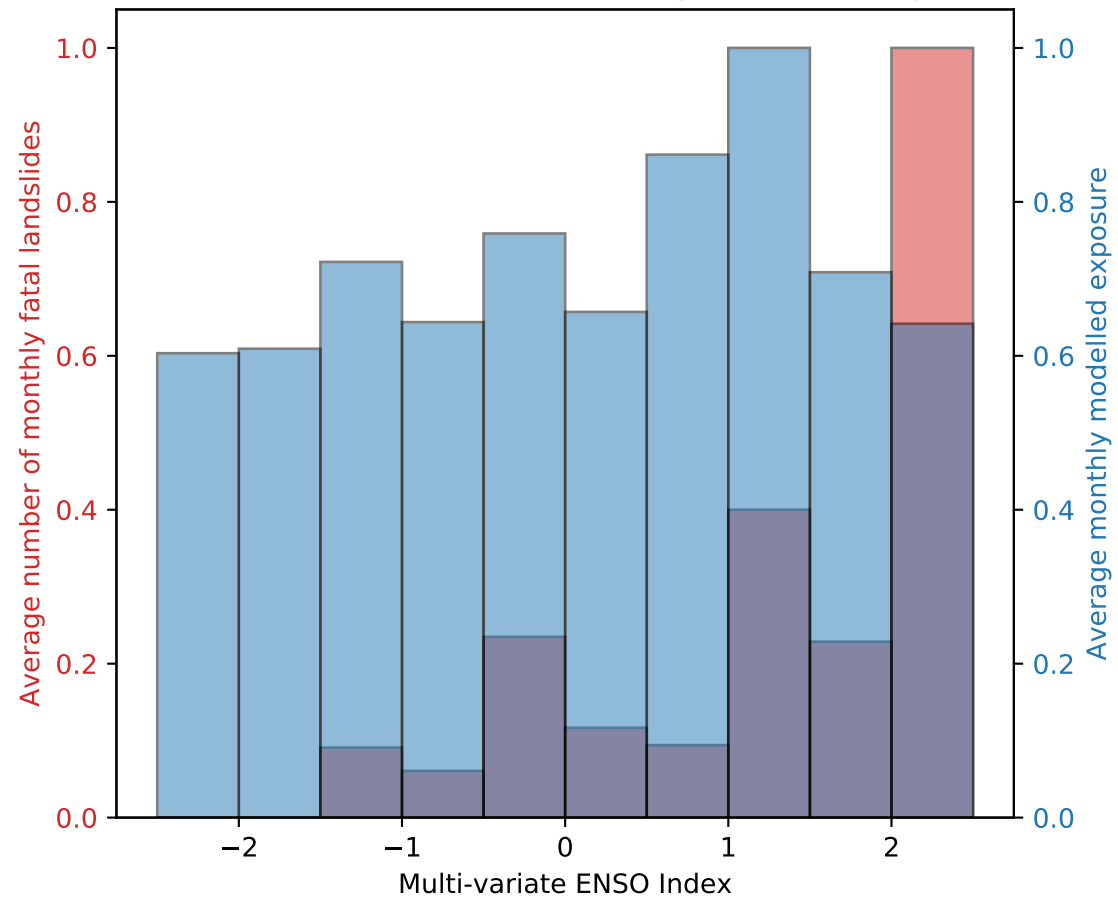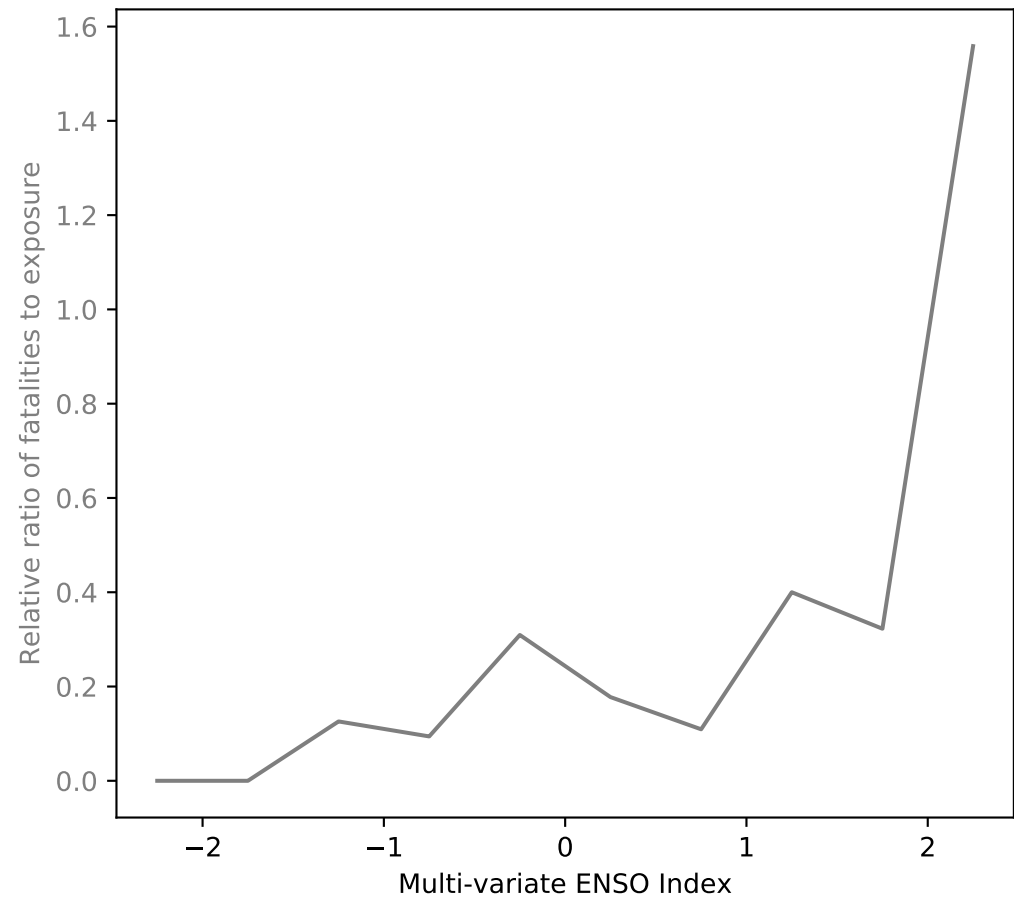

Supplement: Supplementary file 8 — Supplementary Data 5 [file 41467_2021_22398_MOESM8_ESM.zip › In_ENSO_zones/Kenya_landslide_incidence_compare.pdf]

Fatal landslides (GFLD) vs modeled exposure for Malaysia, n= 61

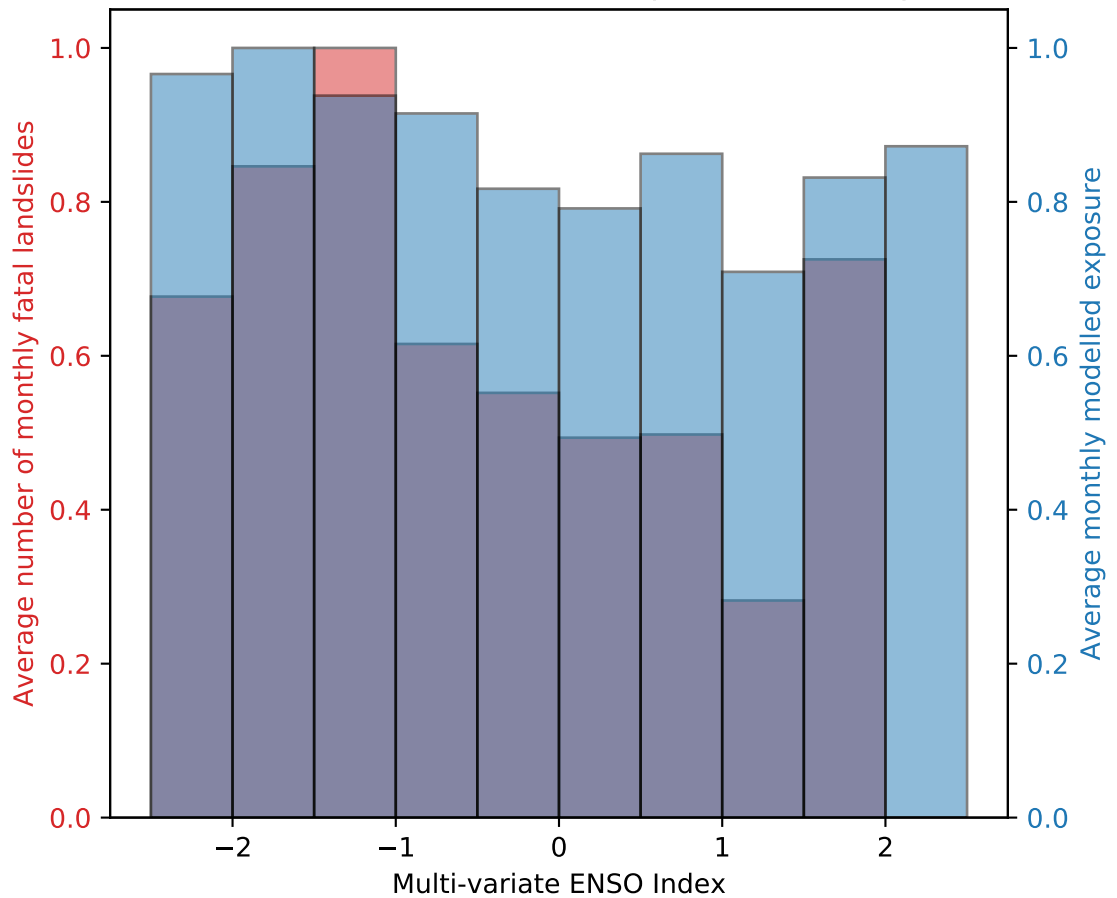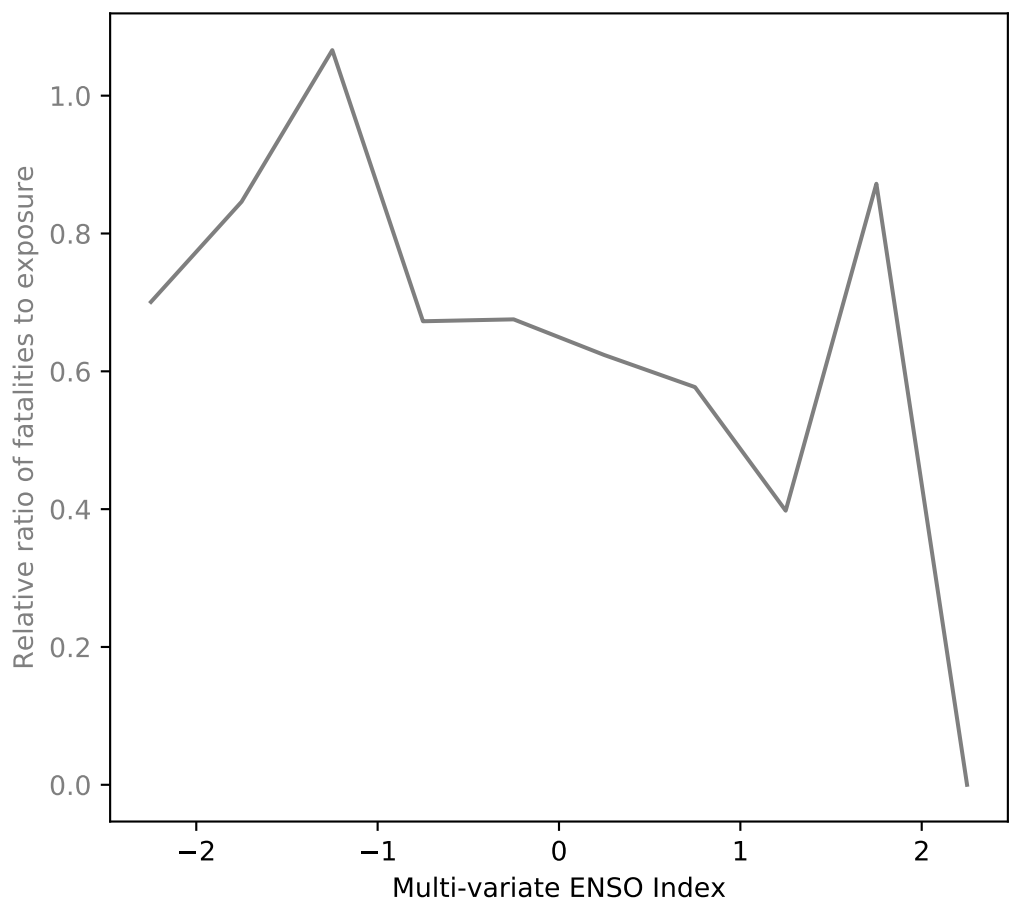

Supplement: Supplementary file 8 — Supplementary Data 5 [file 41467_2021_22398_MOESM8_ESM.zip › In_ENSO_zones/Malaysia_landslide_incidence_compare.pdf]

Fatal landslides (GFLD) vs modeled exposure for Mexico, n= 95

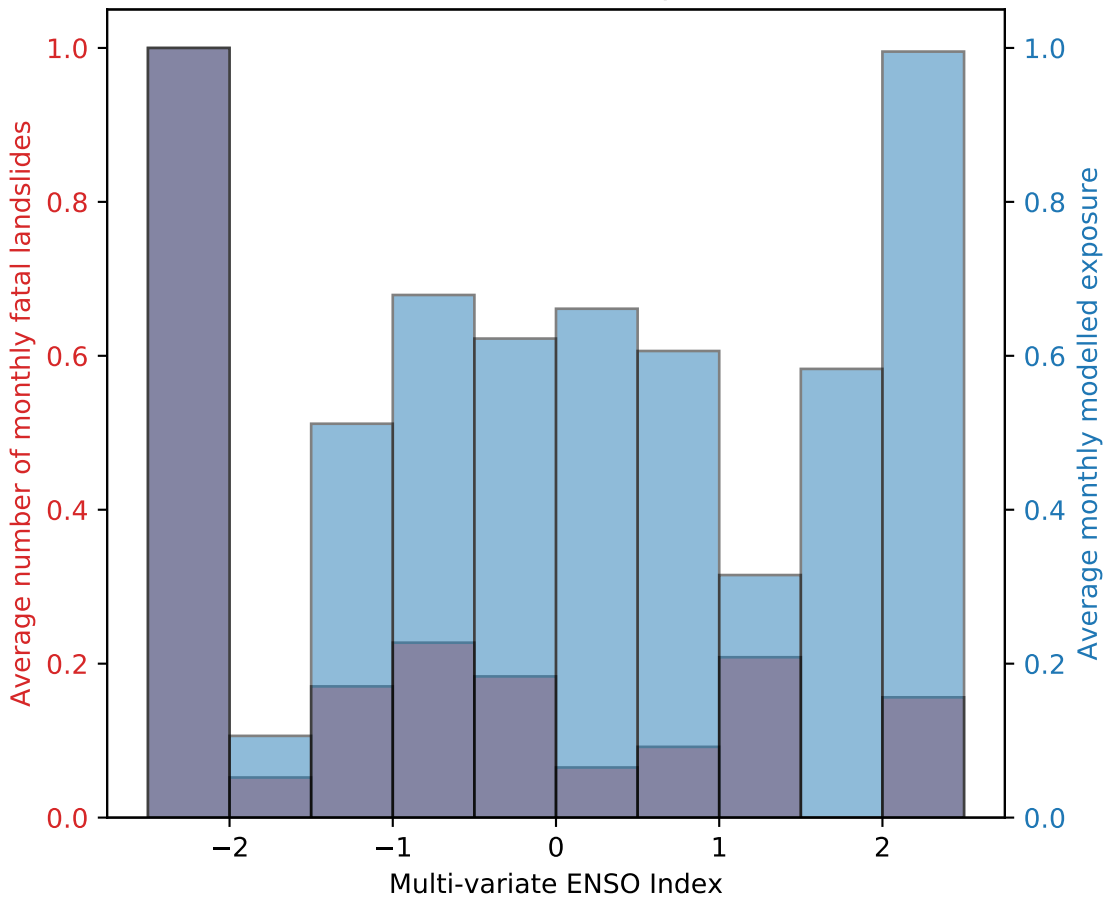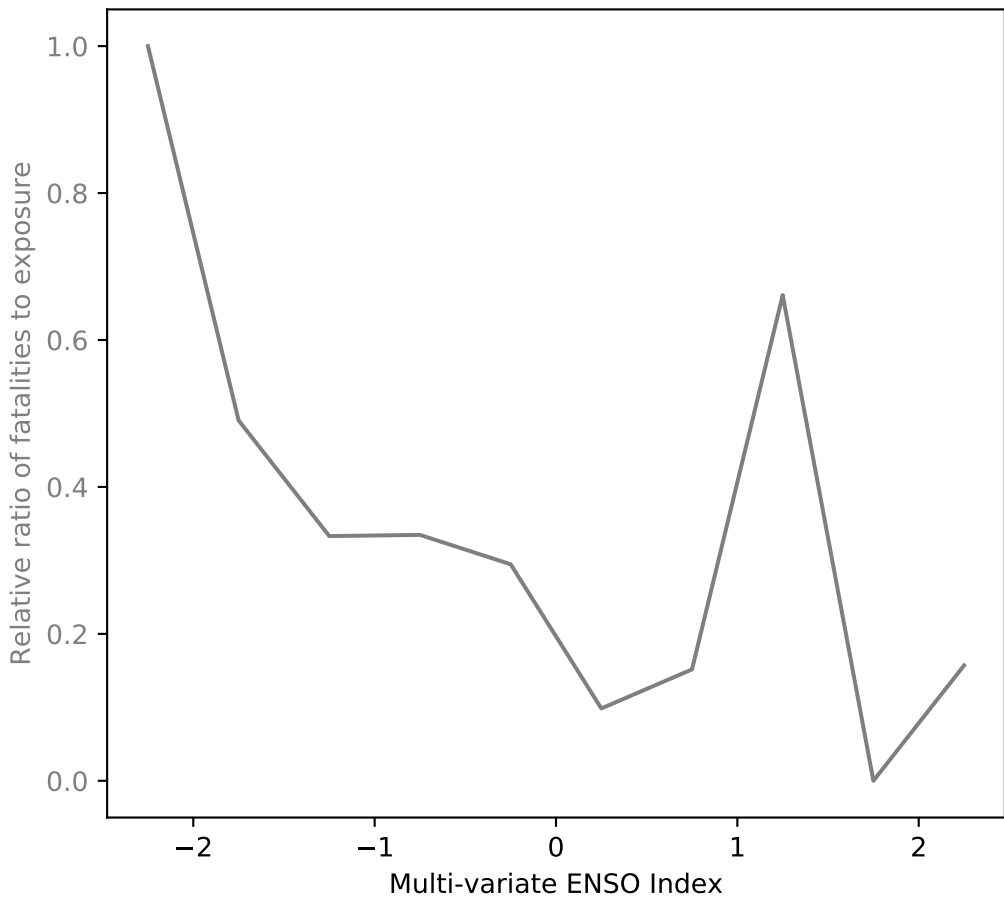

Supplement: Supplementary file 8 — Supplementary Data 5 [file 41467_2021_22398_MOESM8_ESM.zip › In_ENSO_zones/Mexico_landslide_incidence_compare.pdf]

Fatal landslides (GFLD) vs modeled exposure for Myanmar, n= 46

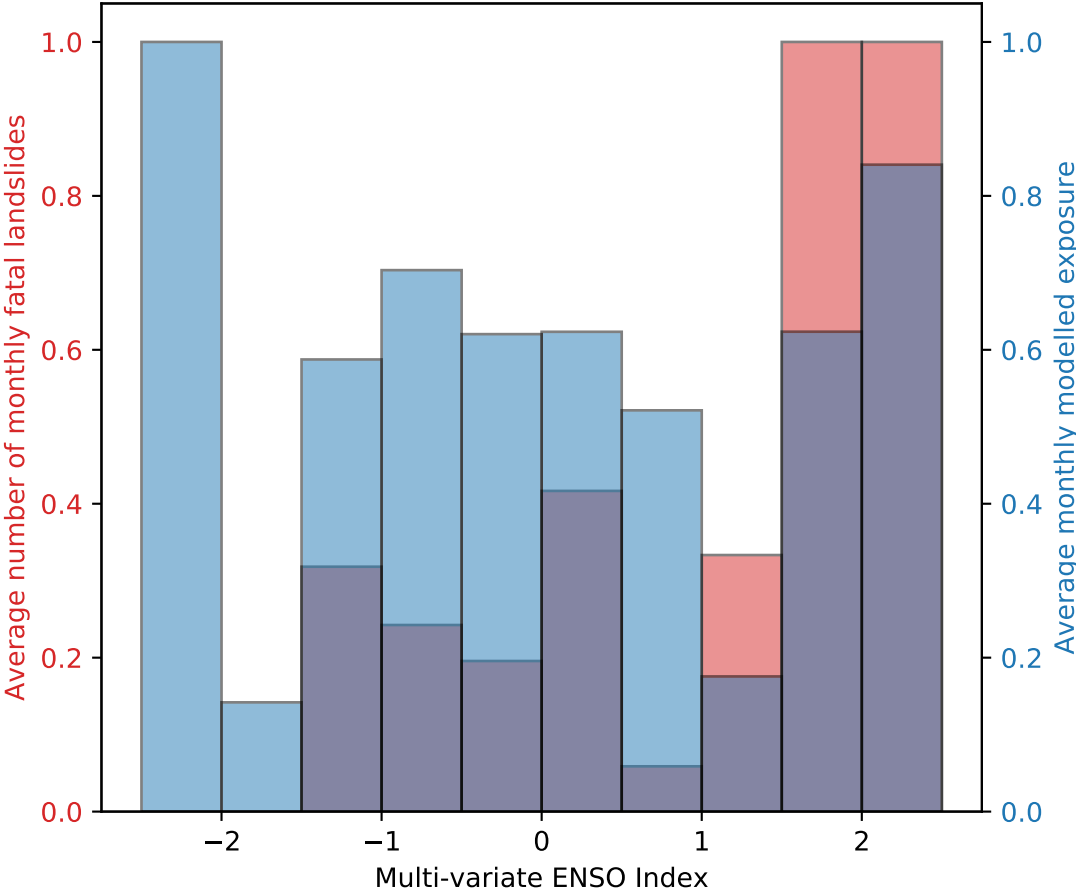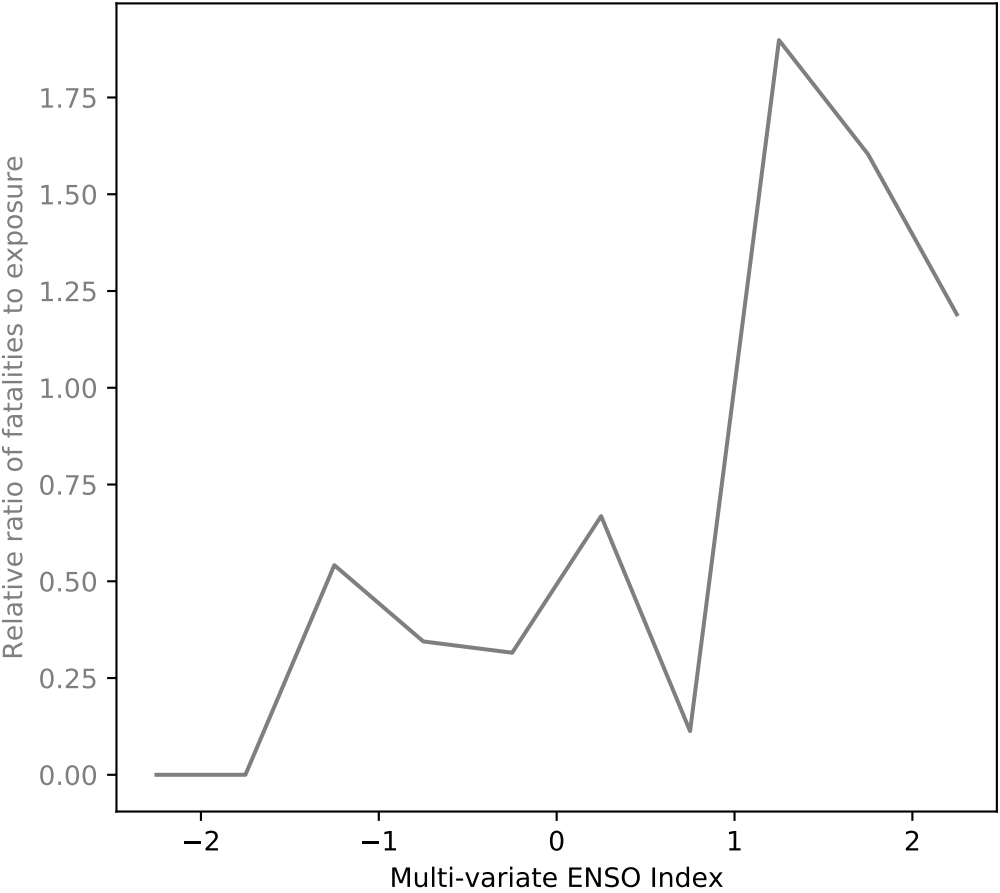

Supplement: Supplementary file 8 — Supplementary Data 5 [file 41467_2021_22398_MOESM8_ESM.zip › In_ENSO_zones/Myanmar_landslide_incidence_compare.pdf]

Fatal landslides (GFLD) vs modeled exposure for Nepal, n= 478

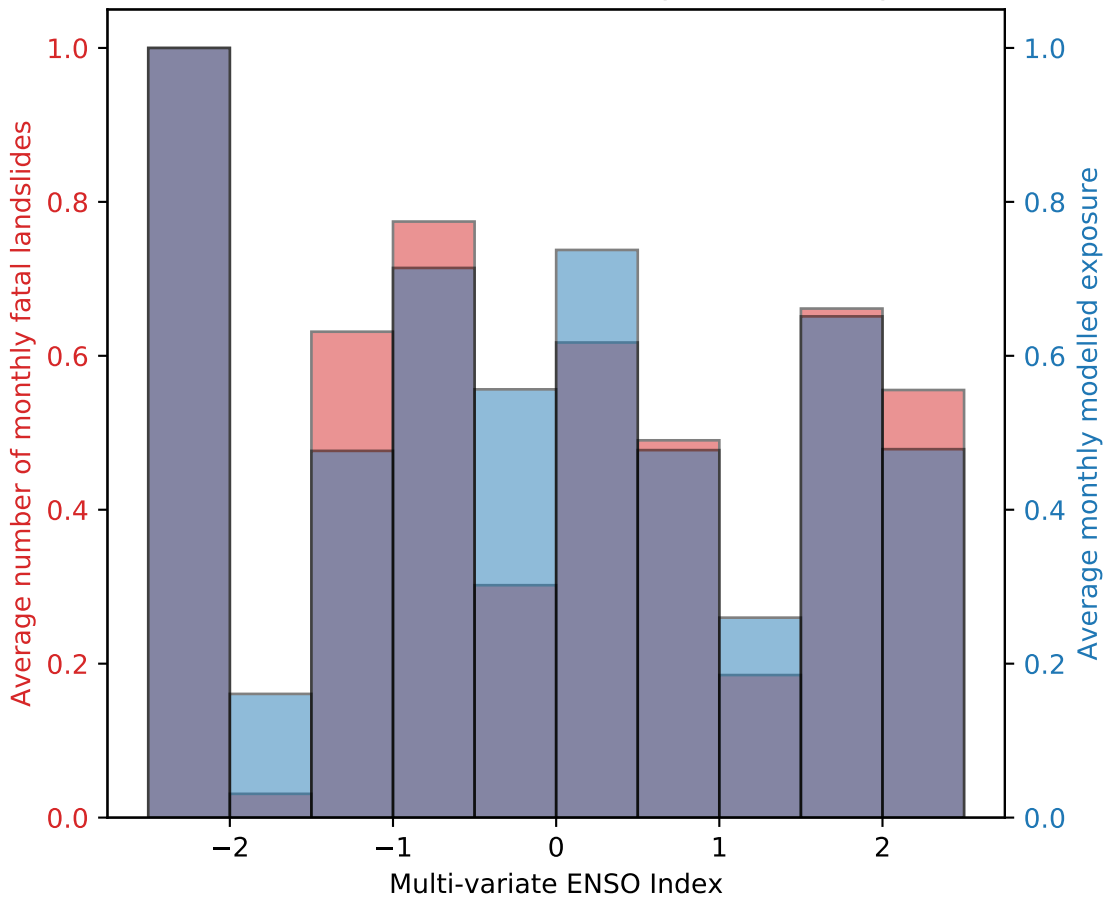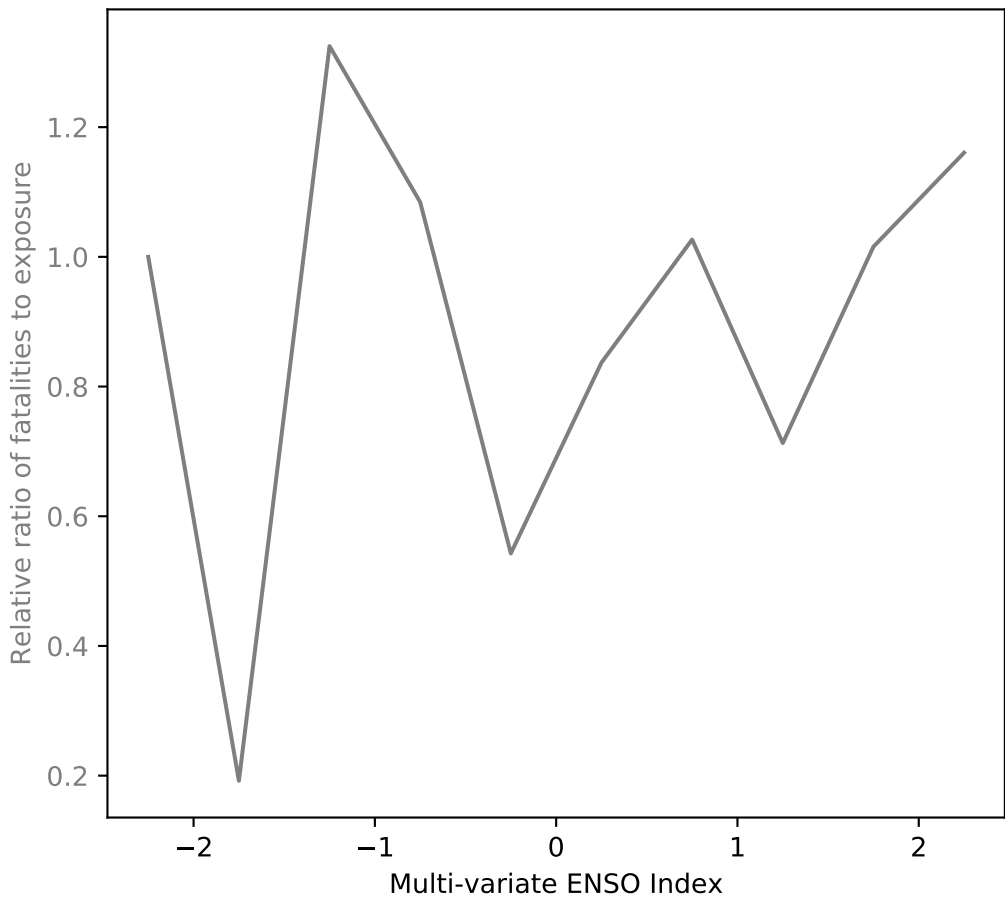

Supplement: Supplementary file 8 — Supplementary Data 5 [file 41467_2021_22398_MOESM8_ESM.zip › In_ENSO_zones/Nepal_landslide_incidence_compare.pdf]

Fatal landslides (GFLD) vs modeled exposure for Pakistan, n= 257

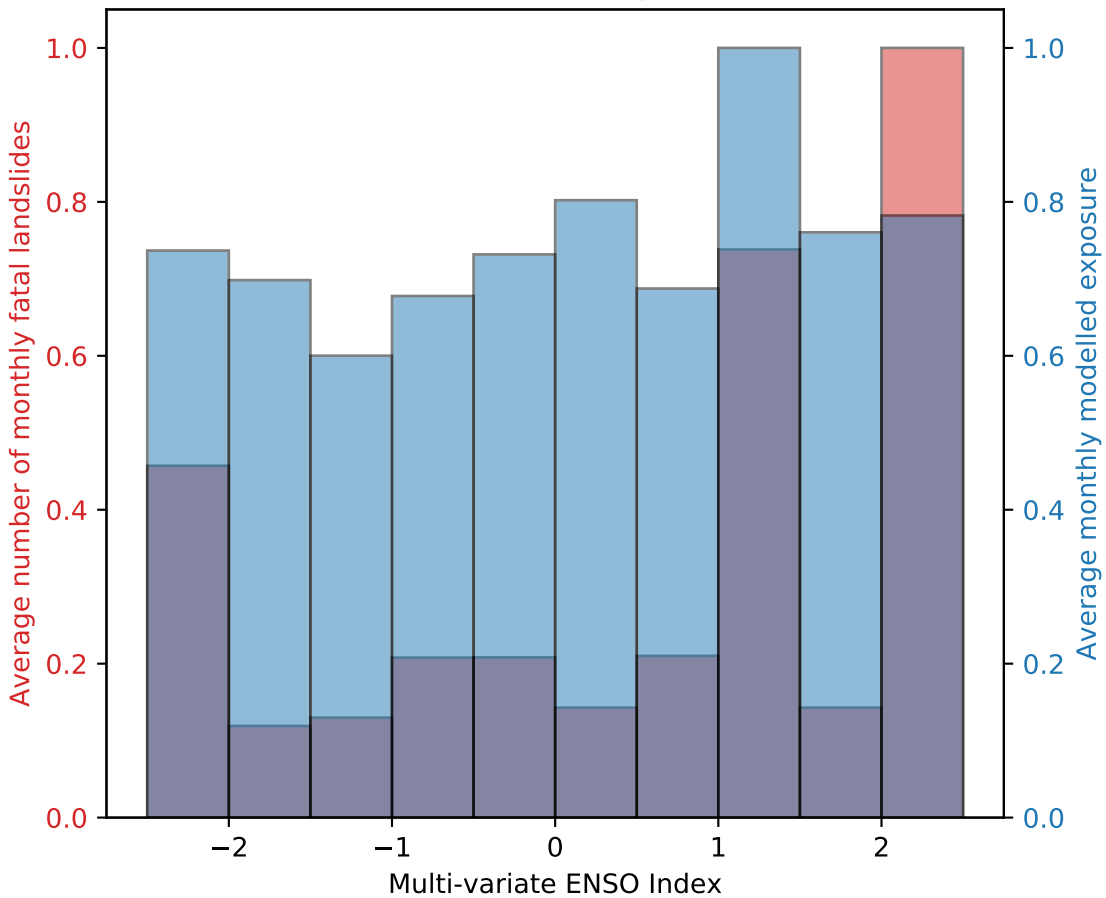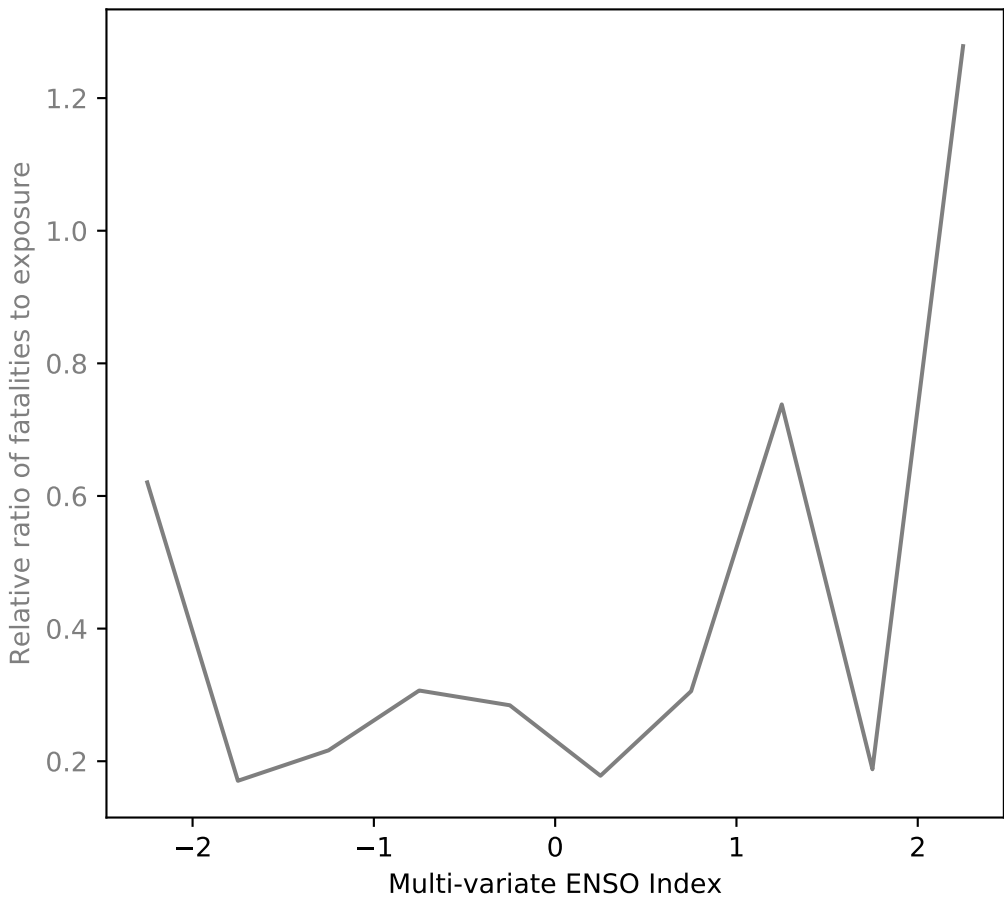

Supplement: Supplementary file 8 — Supplementary Data 5 [file 41467_2021_22398_MOESM8_ESM.zip › In_ENSO_zones/Pakistan_landslide_incidence_compare.pdf]

Fatal landslides (GFLD) vs modeled exposure for Peru, n= 56

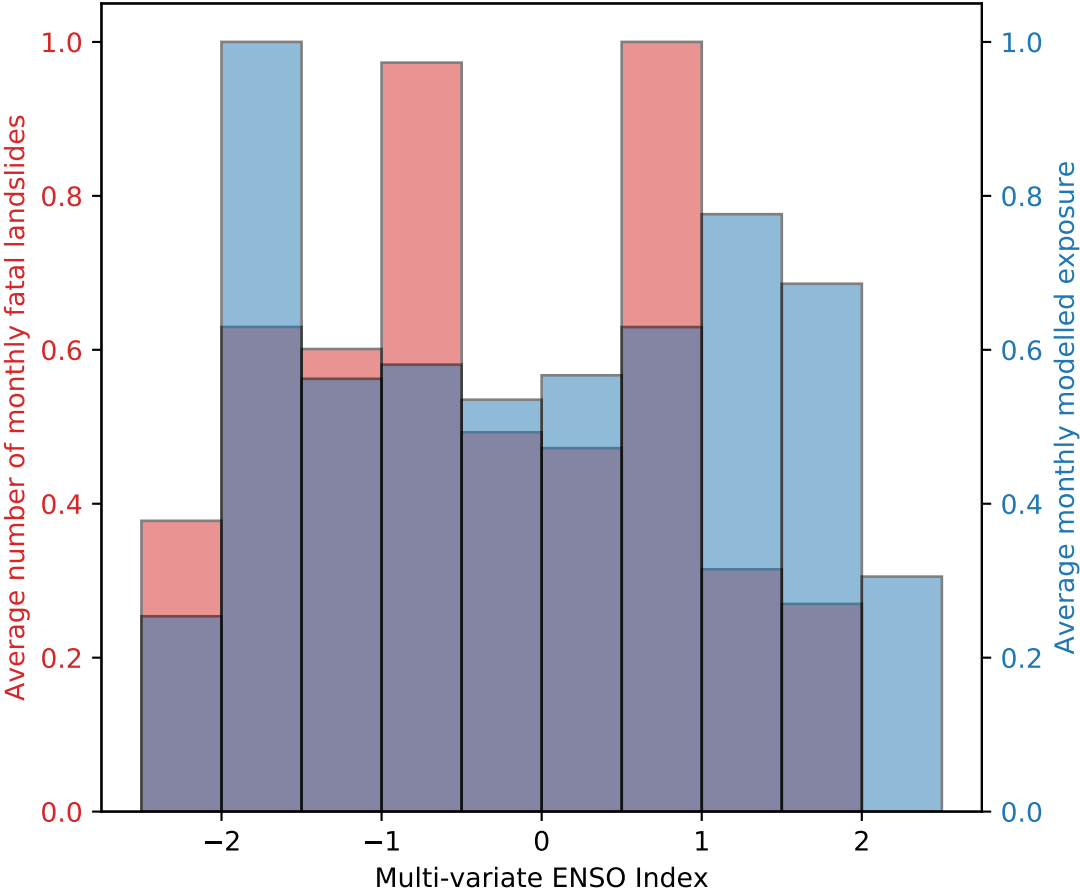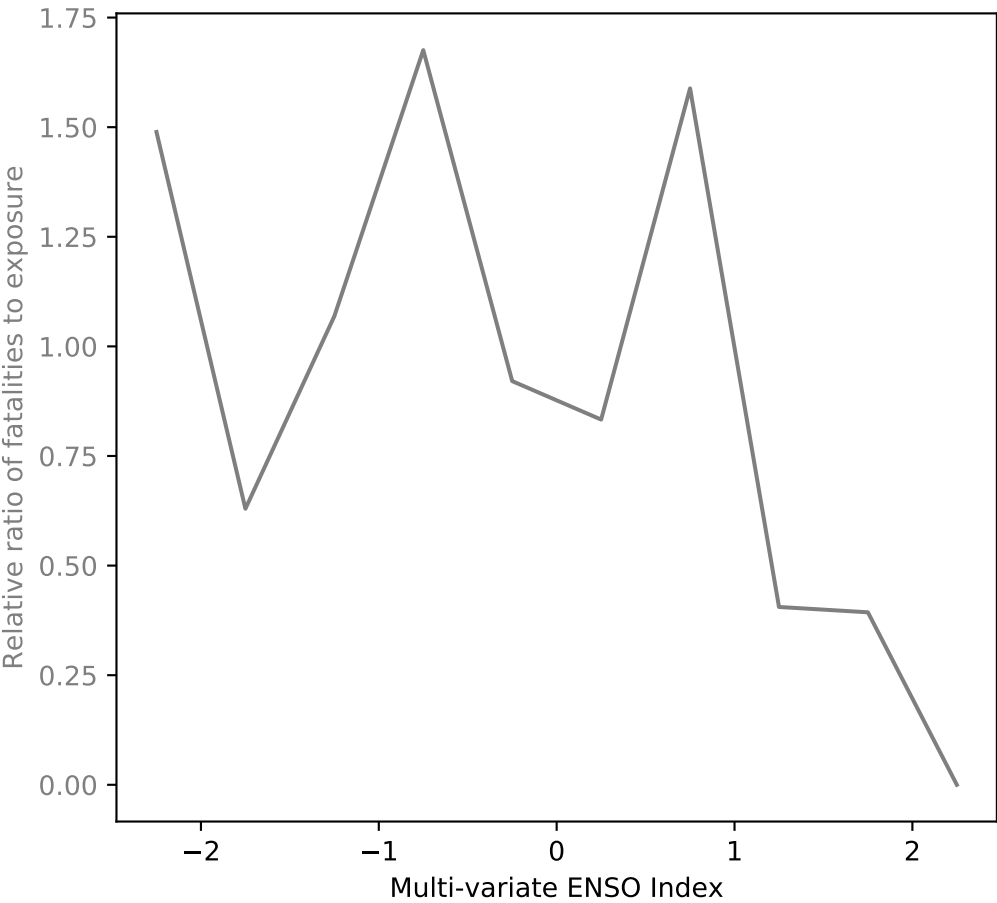

Supplement: Supplementary file 8 — Supplementary Data 5 [file 41467_2021_22398_MOESM8_ESM.zip › In_ENSO_zones/Peru_landslide_incidence_compare.pdf]

Fatal landslides (GFLD) vs modeled exposure for Philippines, n= 479

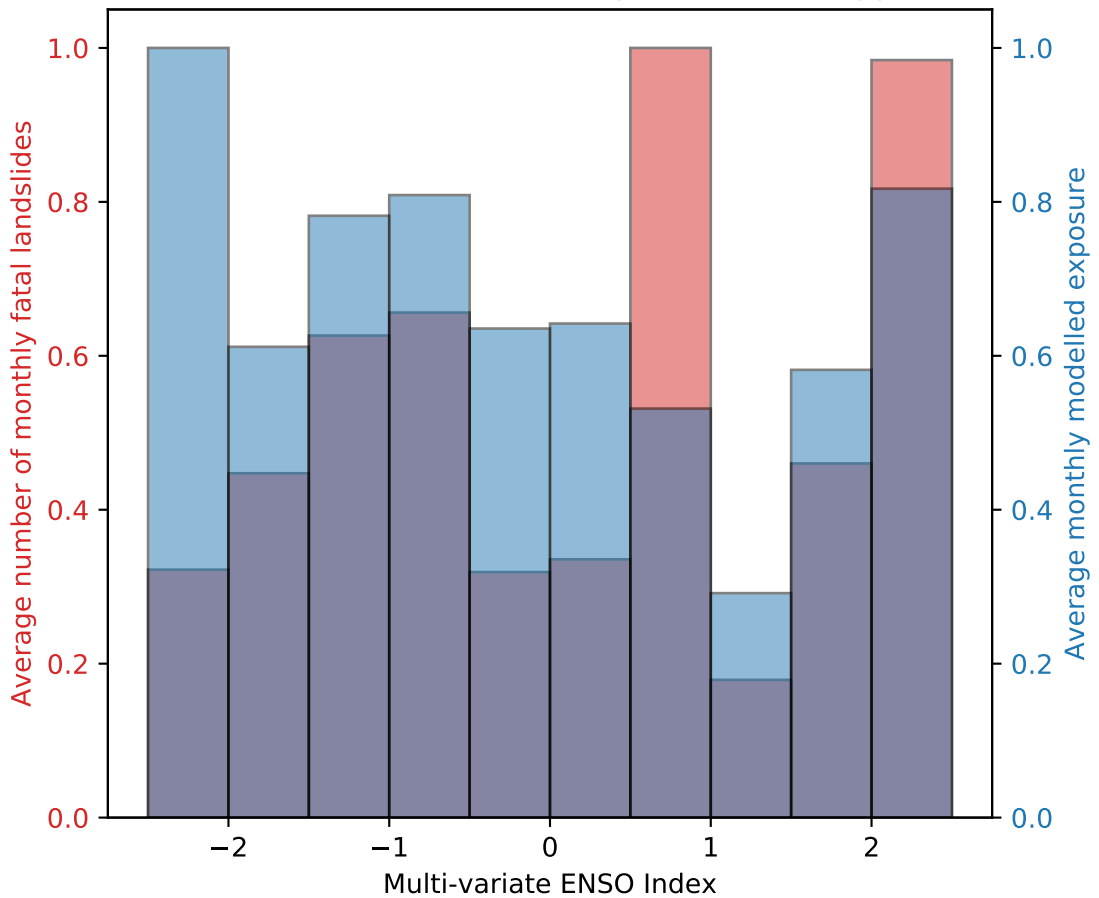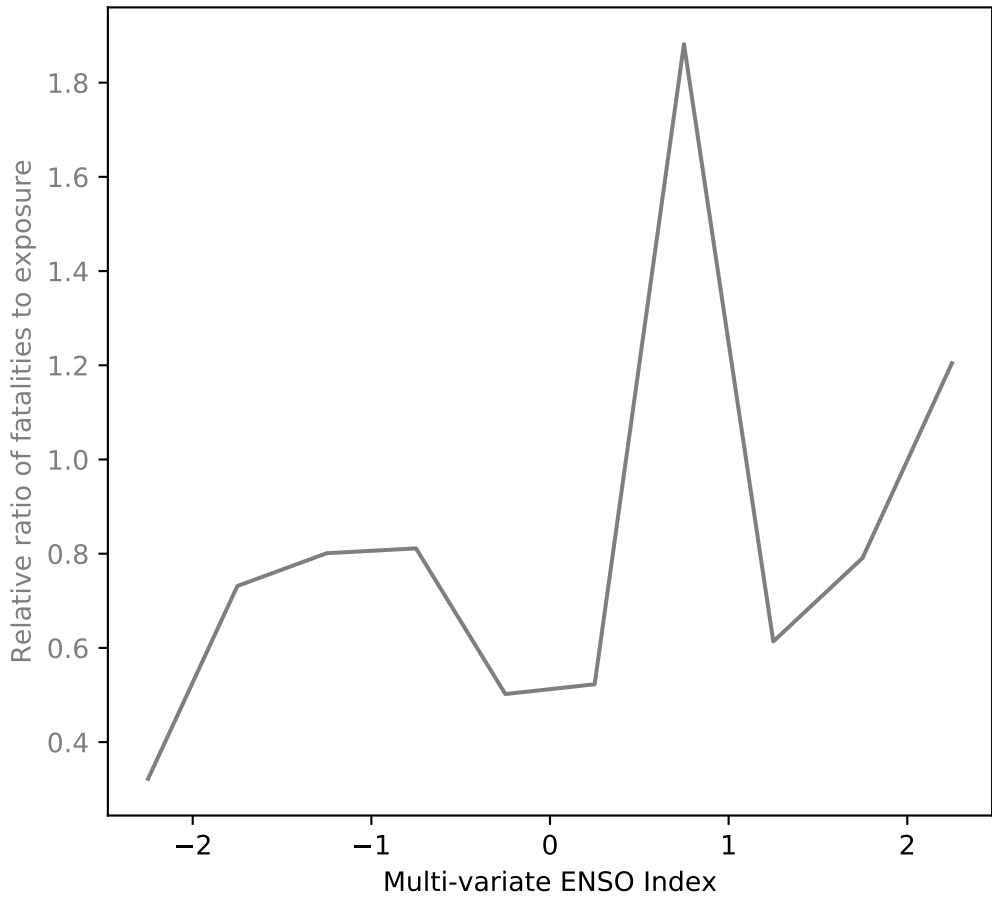

Supplement: Supplementary file 8 — Supplementary Data 5 [file 41467_2021_22398_MOESM8_ESM.zip › In_ENSO_zones/Philippines_landslide_incidence_compare.pdf]

Fatal landslides (GFLD) vs modeled exposure for Thailand, n= 39

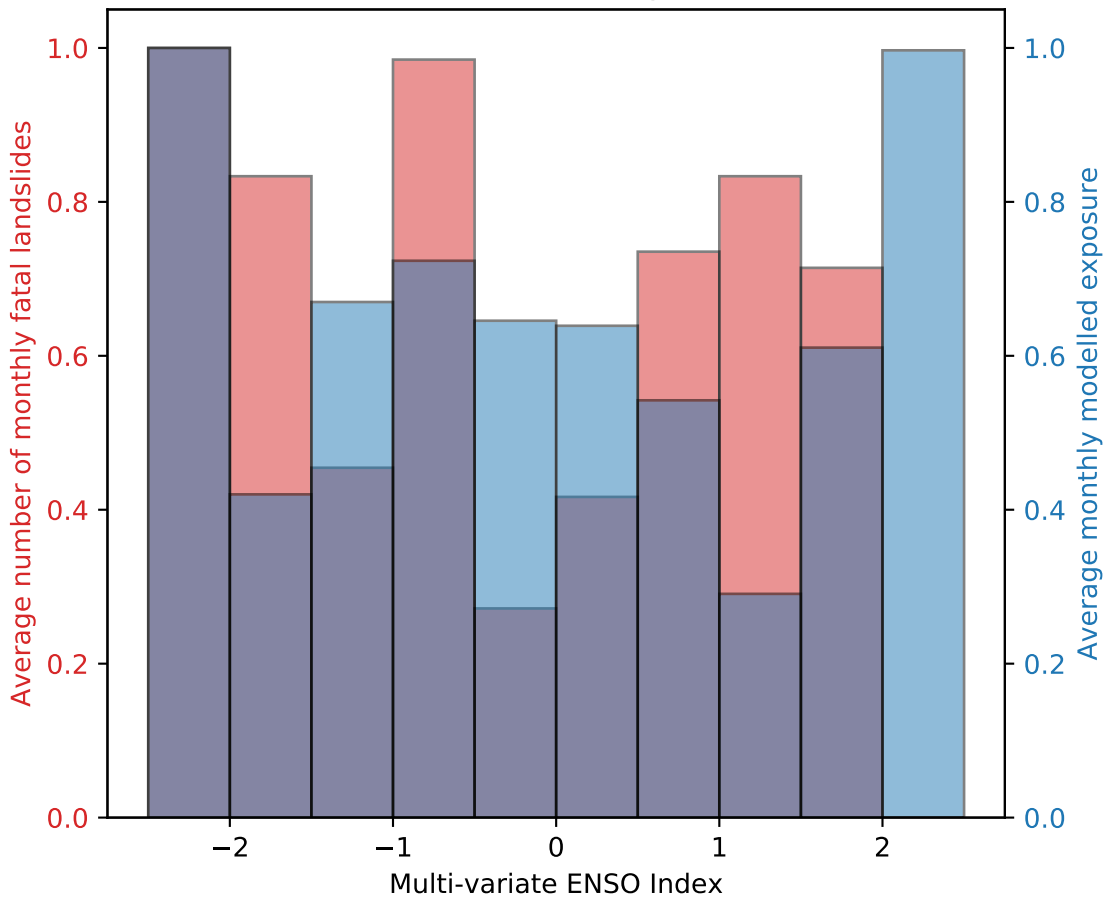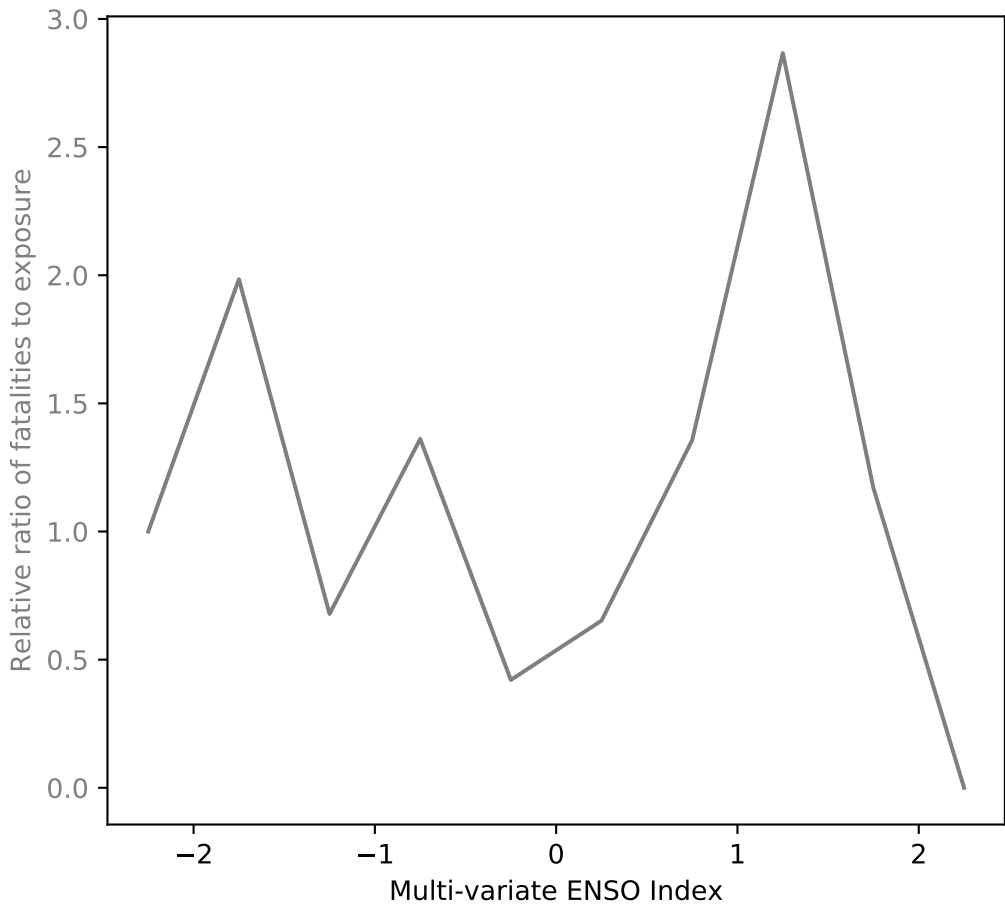

Supplement: Supplementary file 8 — Supplementary Data 5 [file 41467_2021_22398_MOESM8_ESM.zip › In_ENSO_zones/Thailand_landslide_incidence_compare.pdf]

Fatal landslides (GFLD) vs modeled exposure for Uganda, n= 44

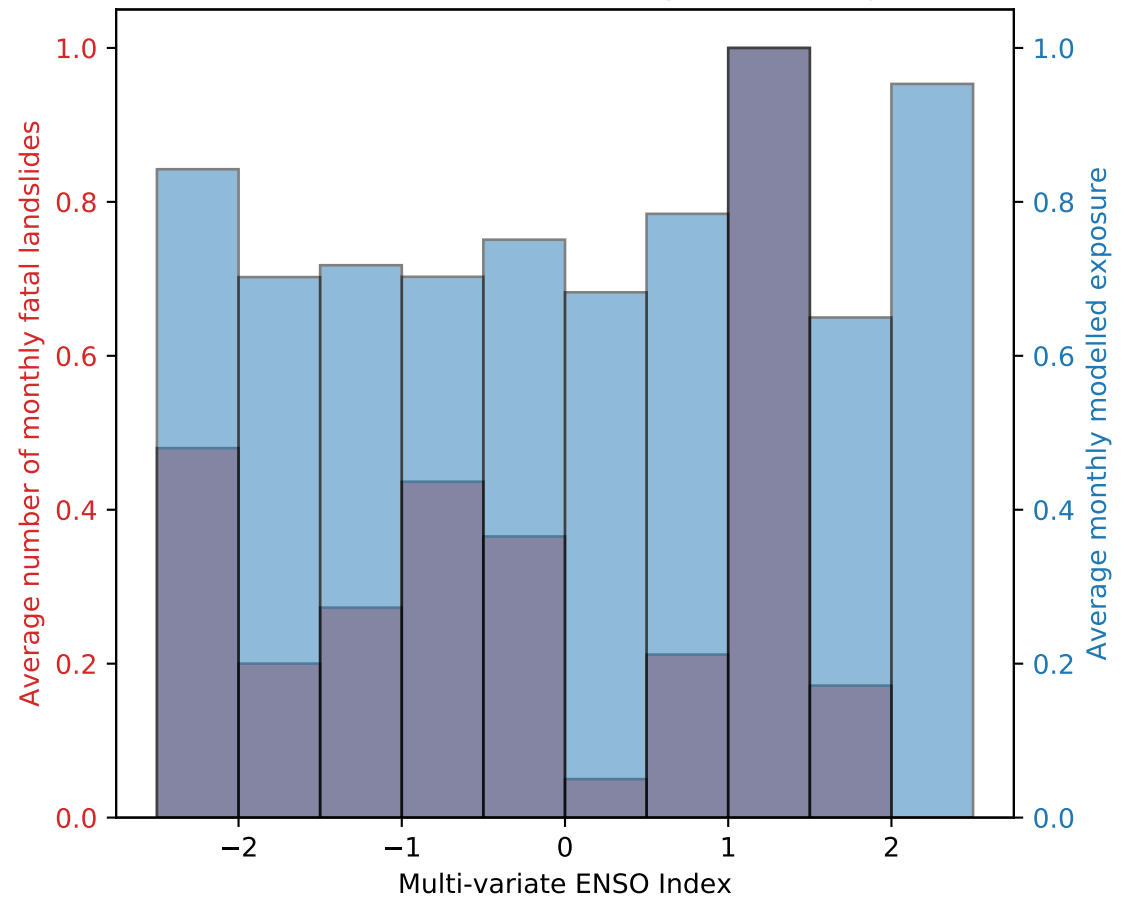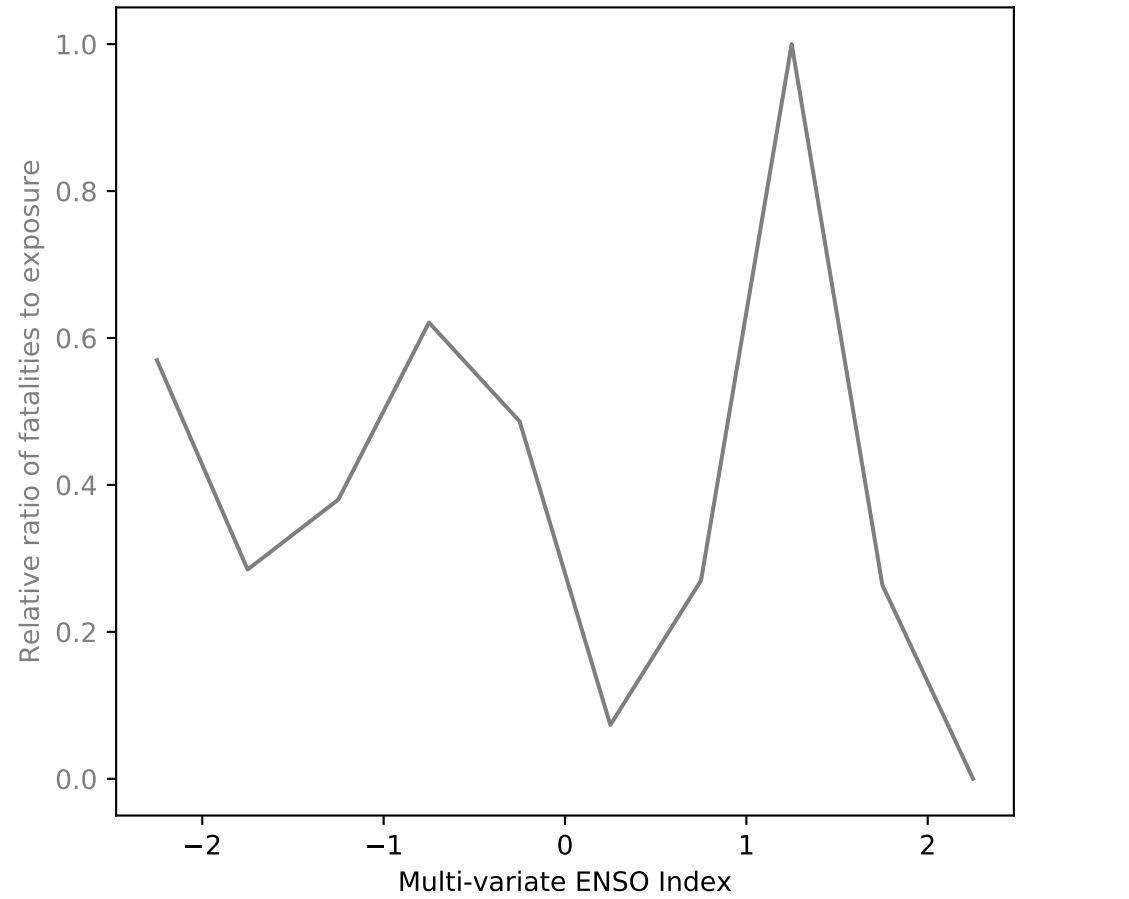

Supplement: Supplementary file 8 — Supplementary Data 5 [file 41467_2021_22398_MOESM8_ESM.zip › In_ENSO_zones/Uganda_landslide_incidence_compare.pdf]

Fatal landslides (GFLD) vs modeled exposure for USA, n= 72

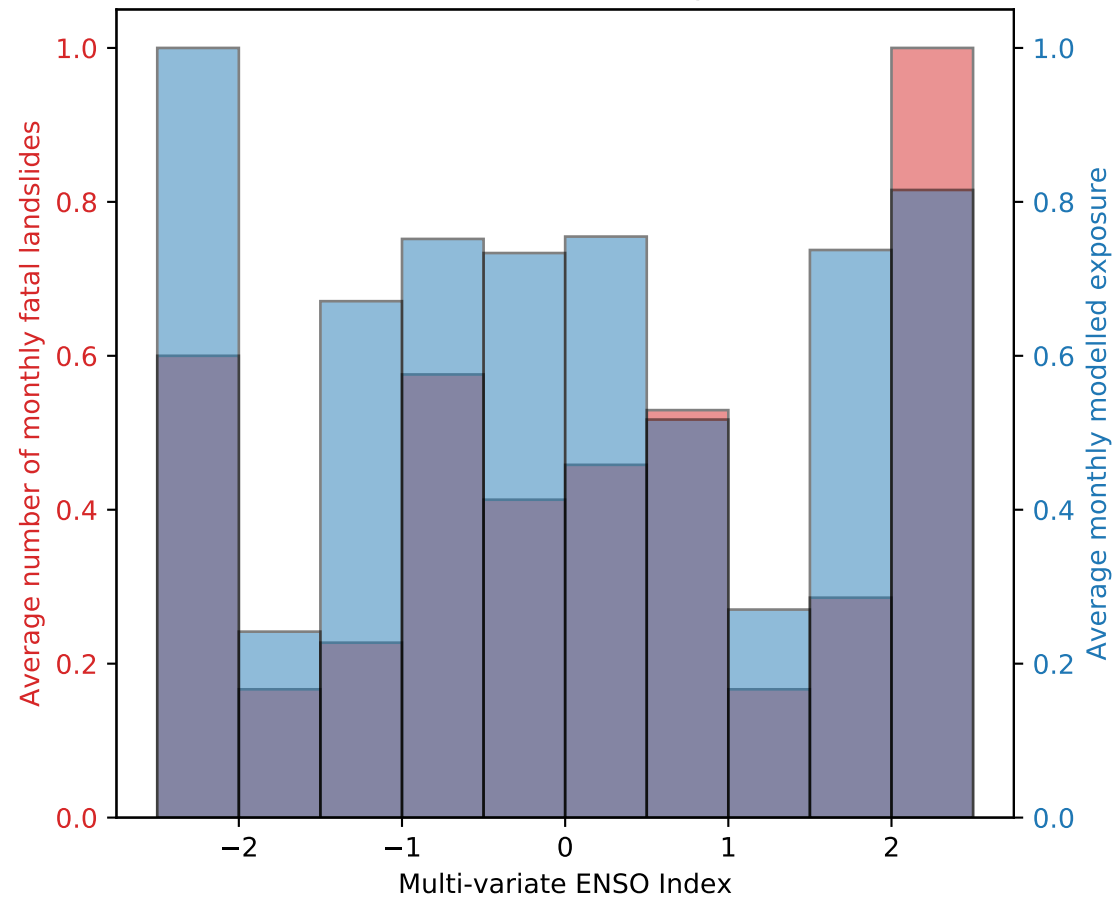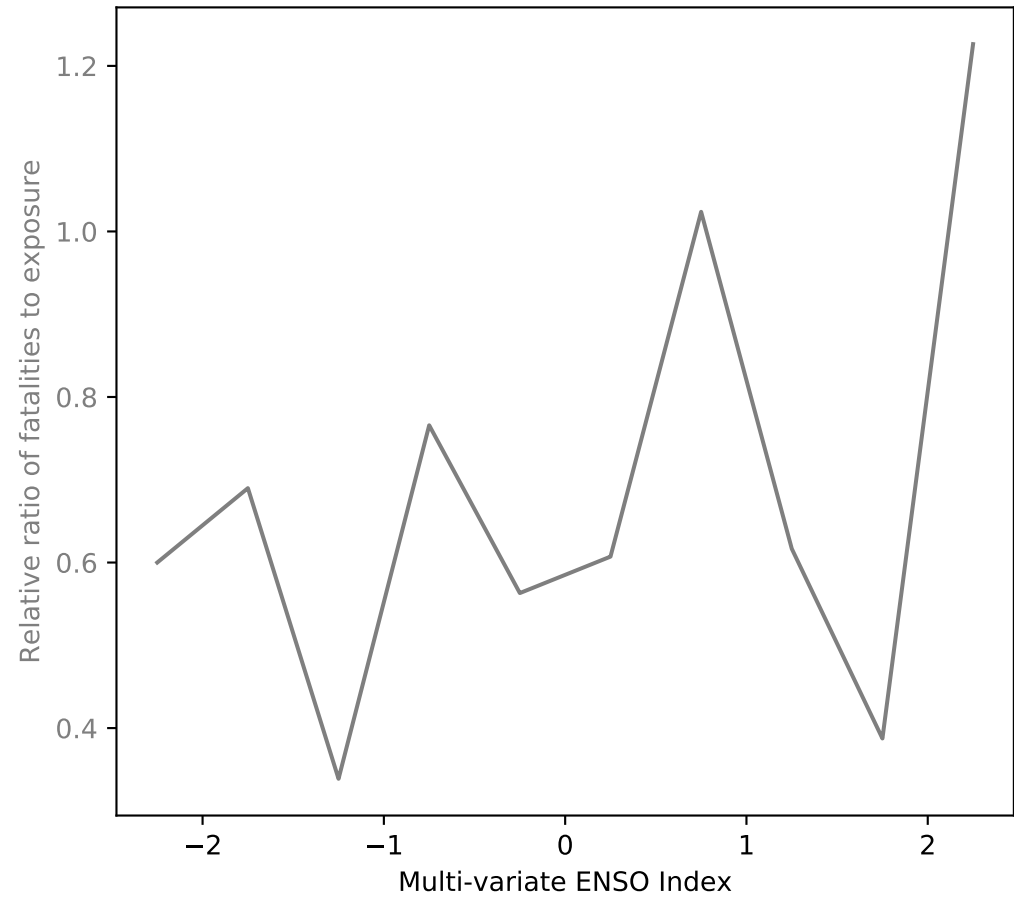

Supplement: Supplementary file 8 — Supplementary Data 5 [file 41467_2021_22398_MOESM8_ESM.zip › In_ENSO_zones/USA_landslide_incidence_compare.pdf]

Fatal landslides (GFLD) vs modeled exposure for Vietnam, n= 144

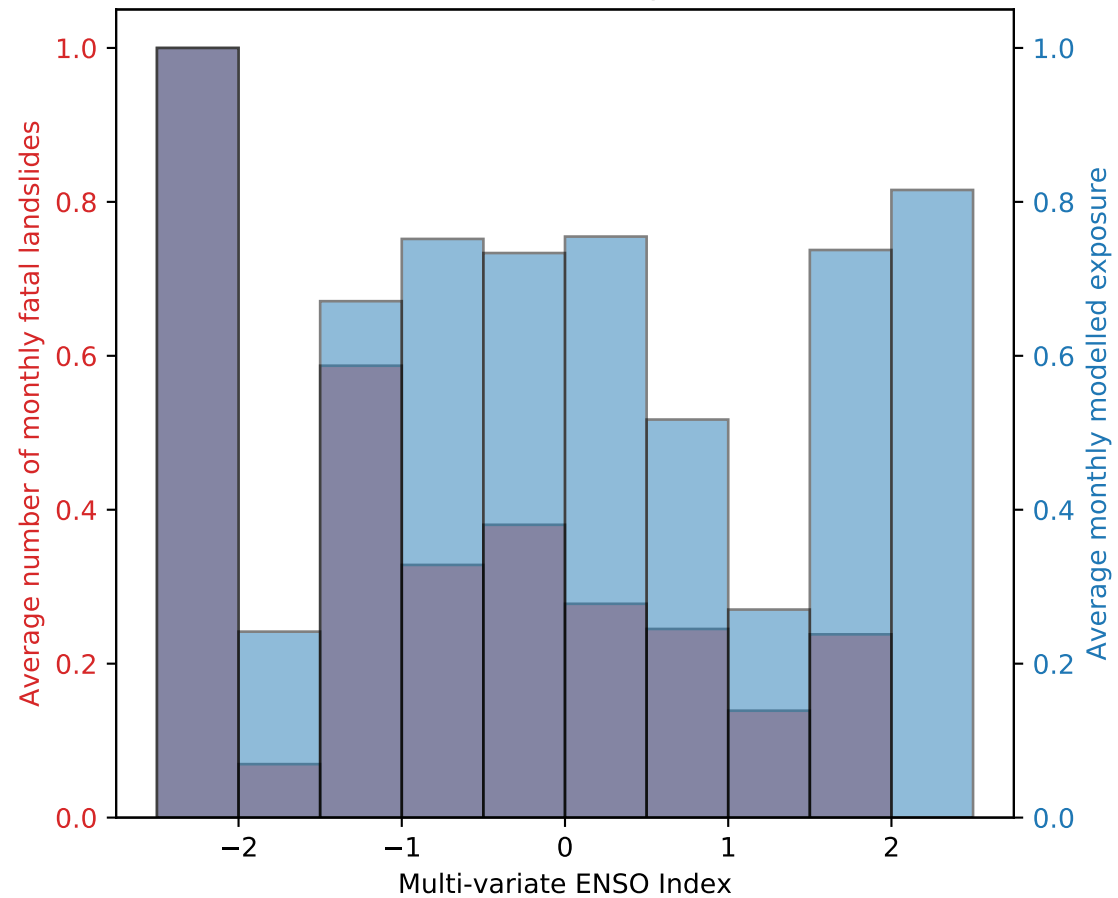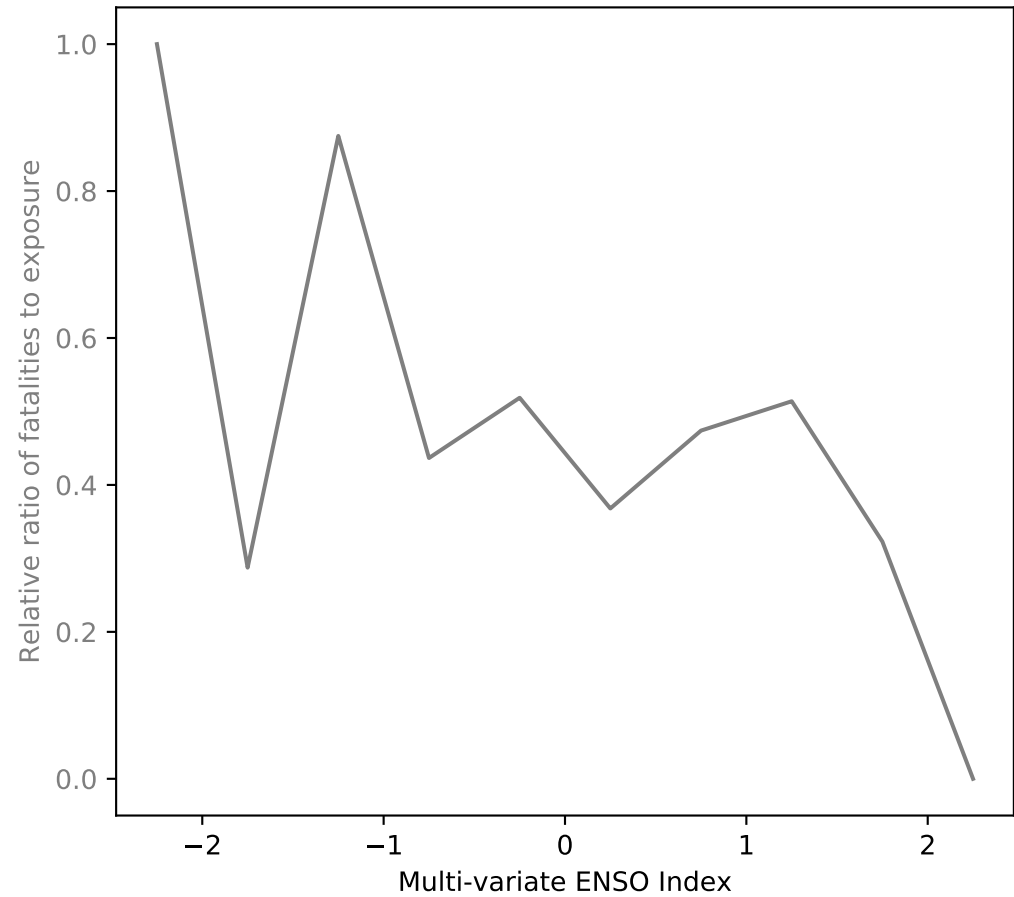

Supplement: Supplementary file 8 — Supplementary Data 5 [file 41467_2021_22398_MOESM8_ESM.zip › In_ENSO_zones/Vietnam_landslide_incidence_compare.pdf]

Fatal landslides (GFLD) vs modeled exposure for Italy, n= 37

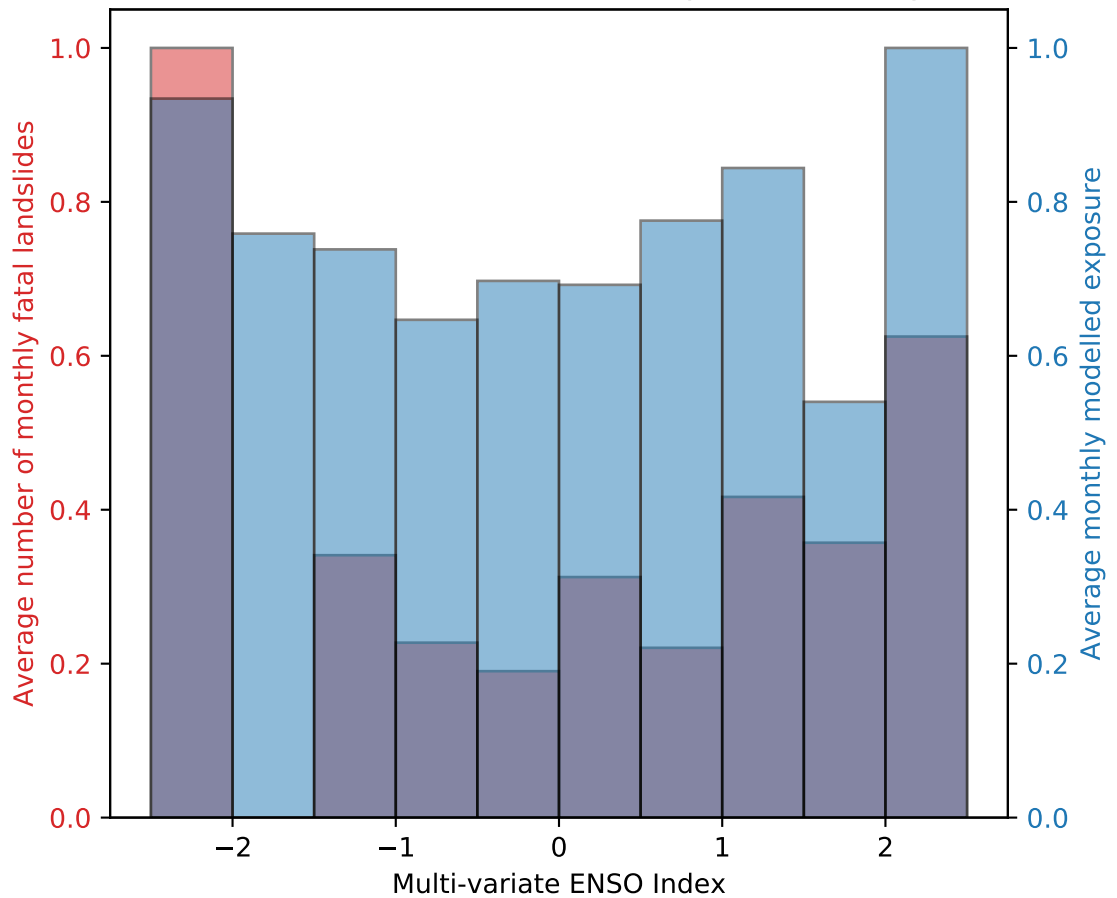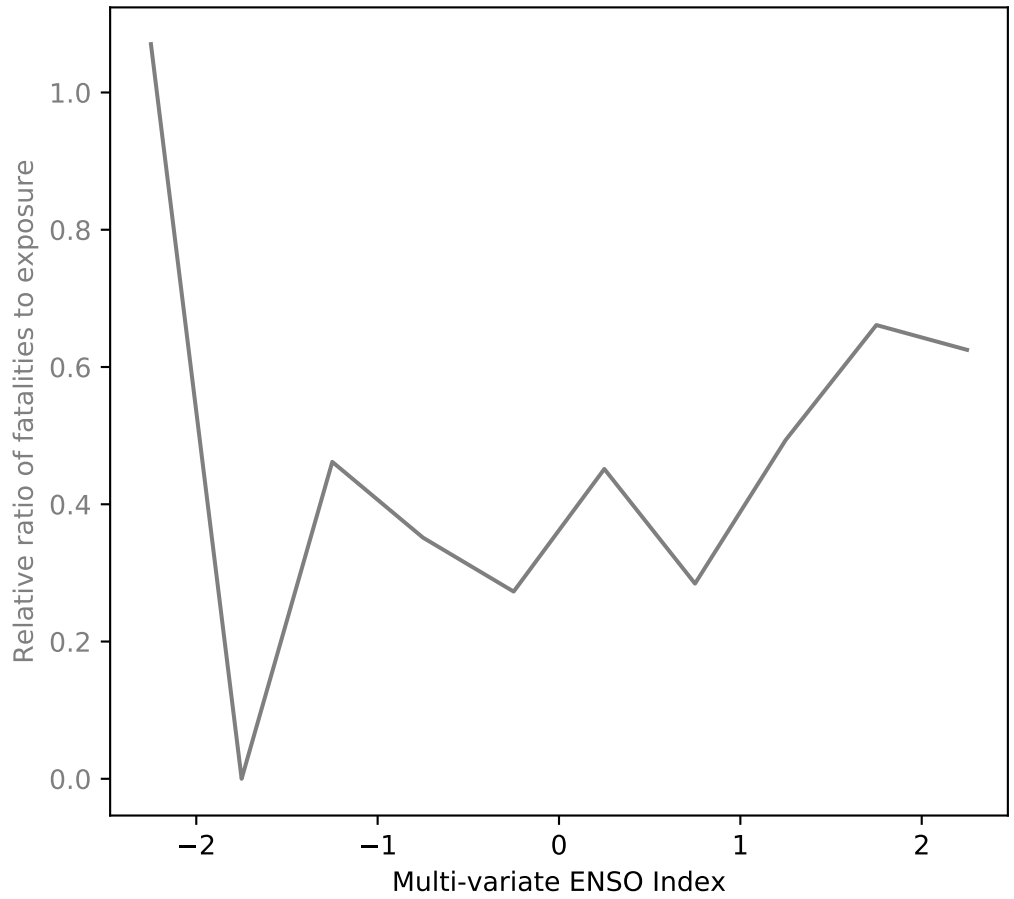

Supplement: Supplementary file 8 — Supplementary Data 5 [file 41467_2021_22398_MOESM8_ESM.zip › Italy_landslide_incidence_compare.pdf]

Fatal landslides (GFLD) vs modeled exposure for Japan, n= 112

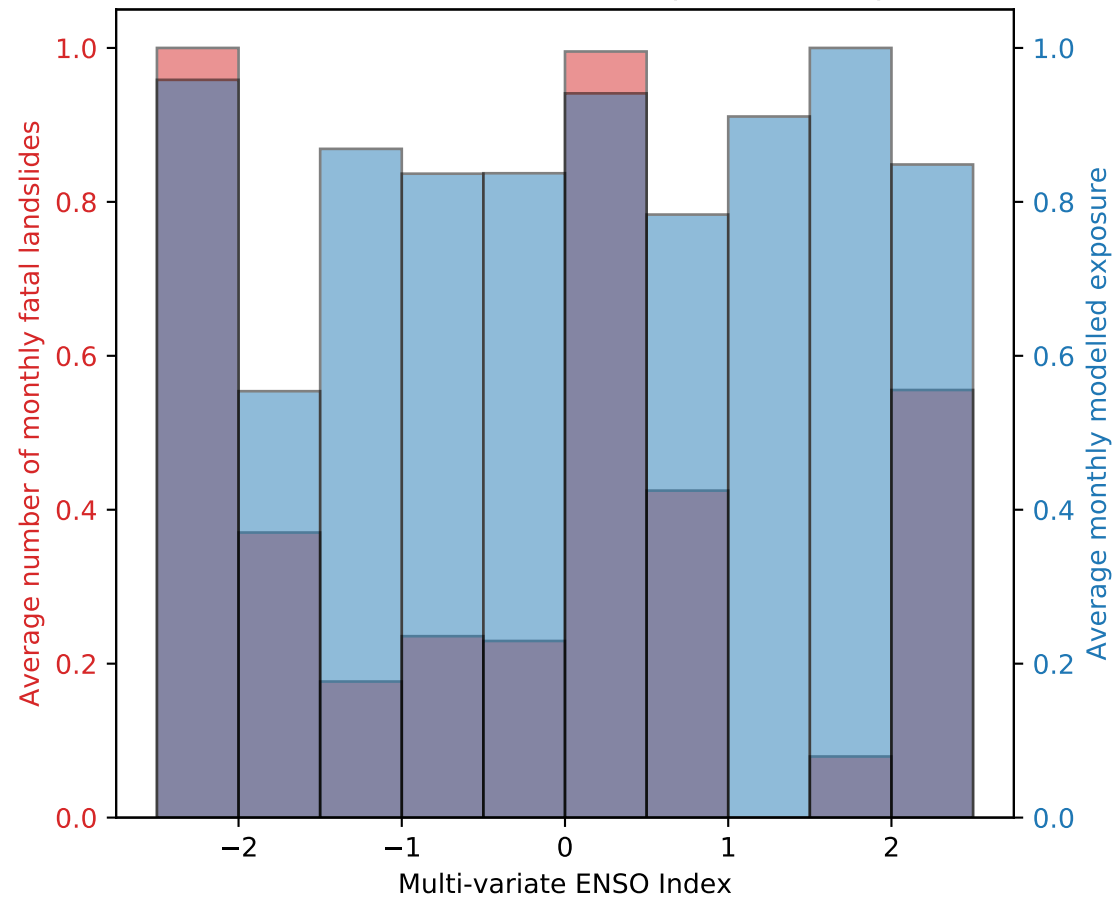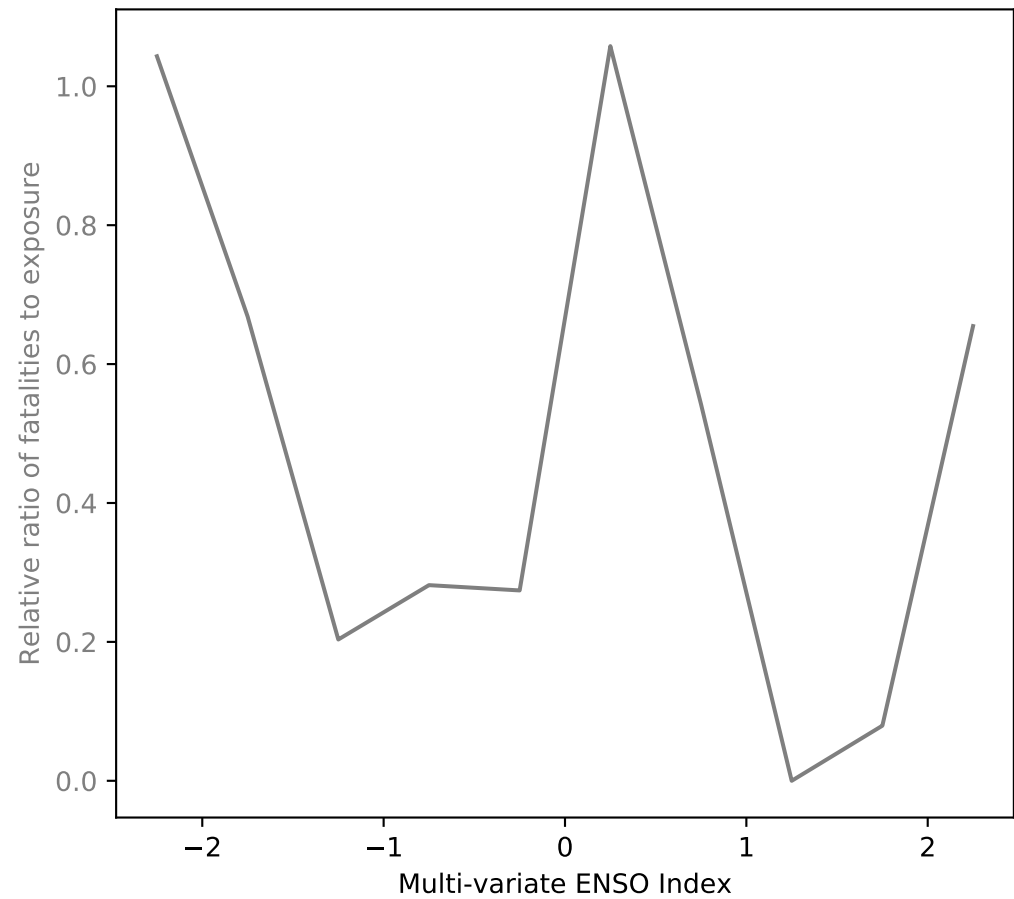

Supplement: Supplementary file 8 — Supplementary Data 5 [file 41467_2021_22398_MOESM8_ESM.zip › Japan_landslide_incidence_compare.pdf]

Fatal landslides (GFLD) vs modeled exposure for South Korea, n= 30

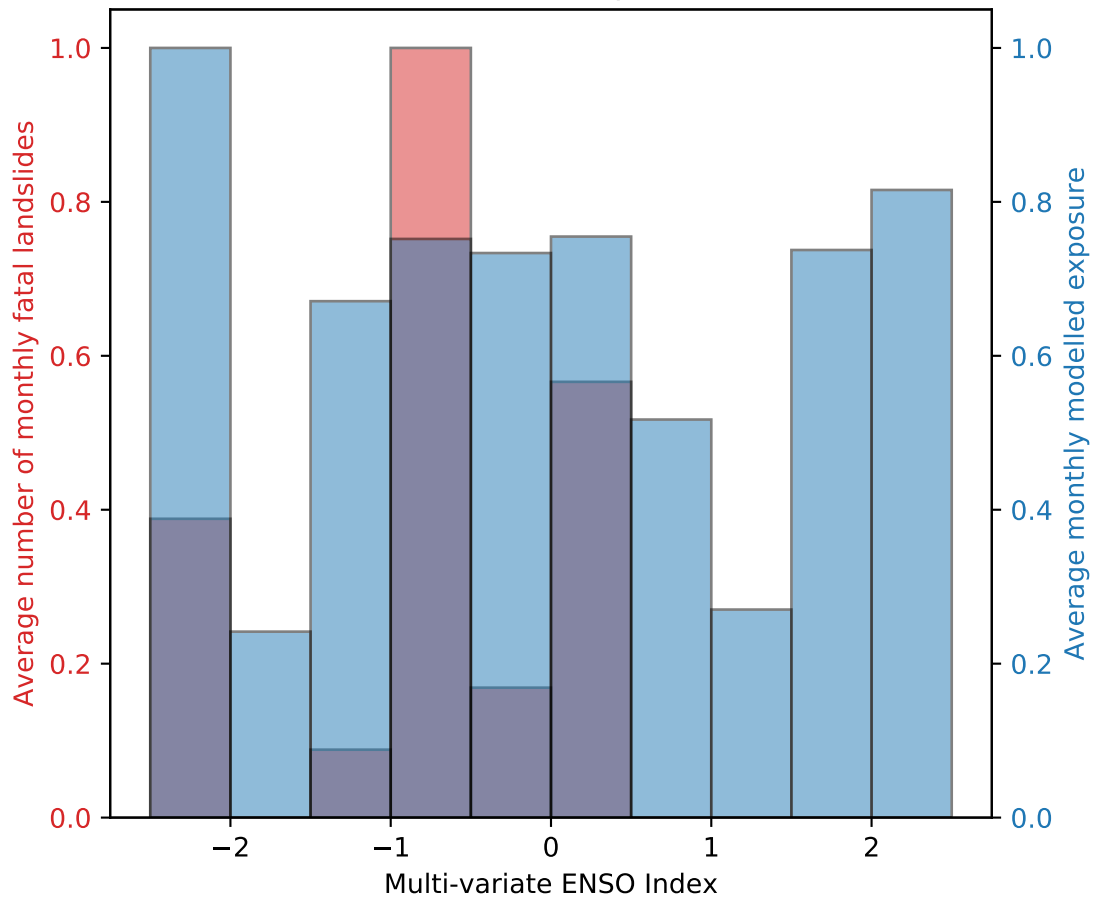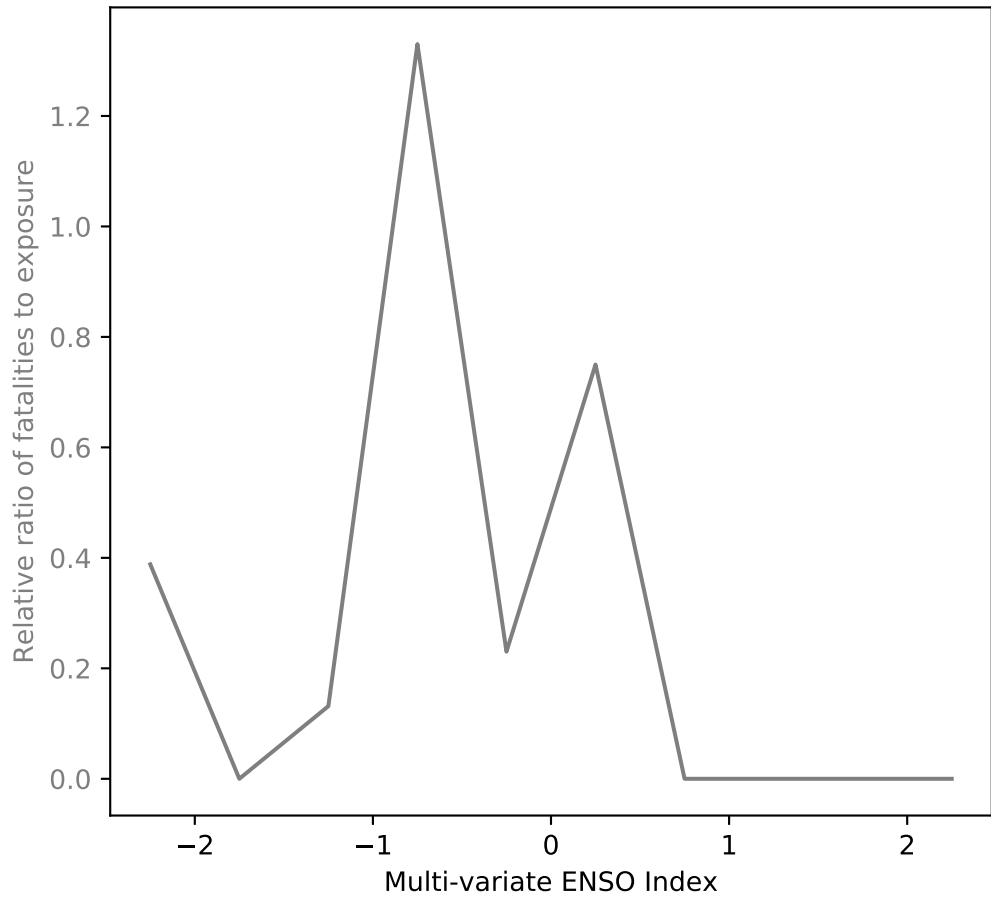

Supplement: Supplementary file 8 — Supplementary Data 5 [file 41467_2021_22398_MOESM8_ESM.zip › South Korea_landslide_incidence_compare.pdf]

Fatal landslides (GFLD) vs modeled exposure for Sri Lanka, n= 81

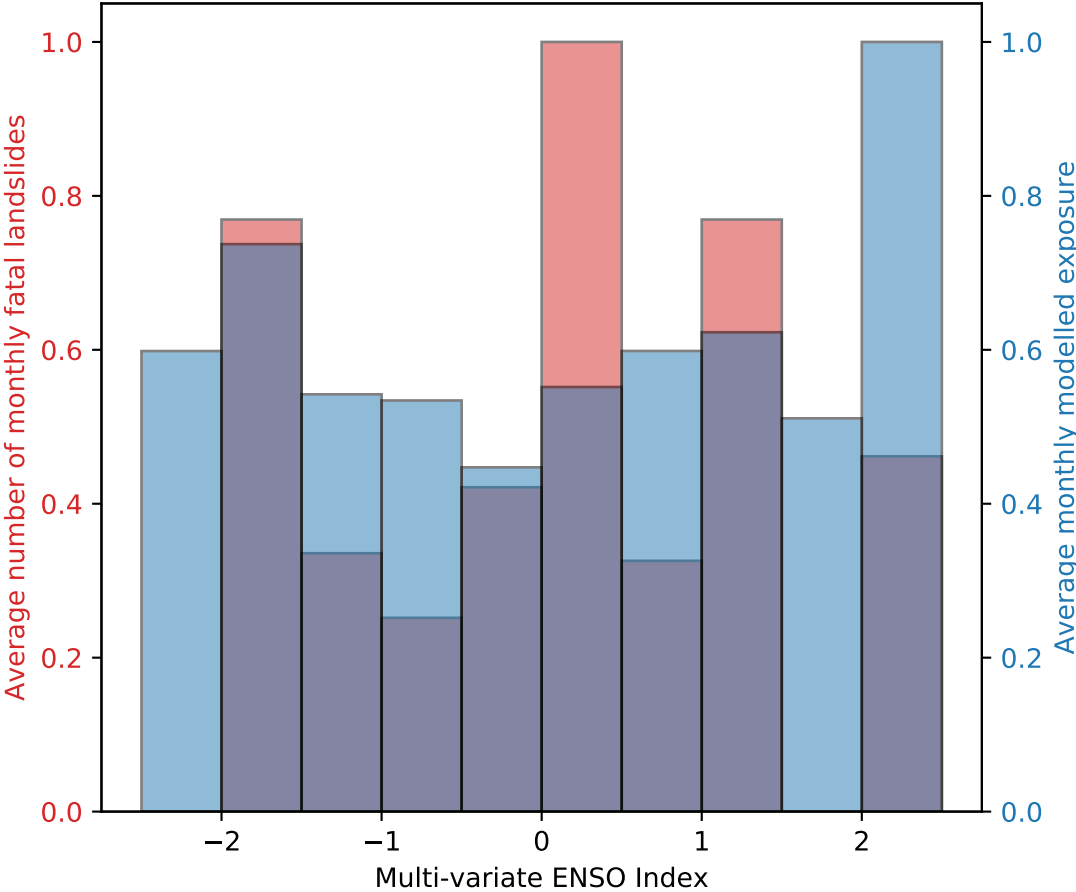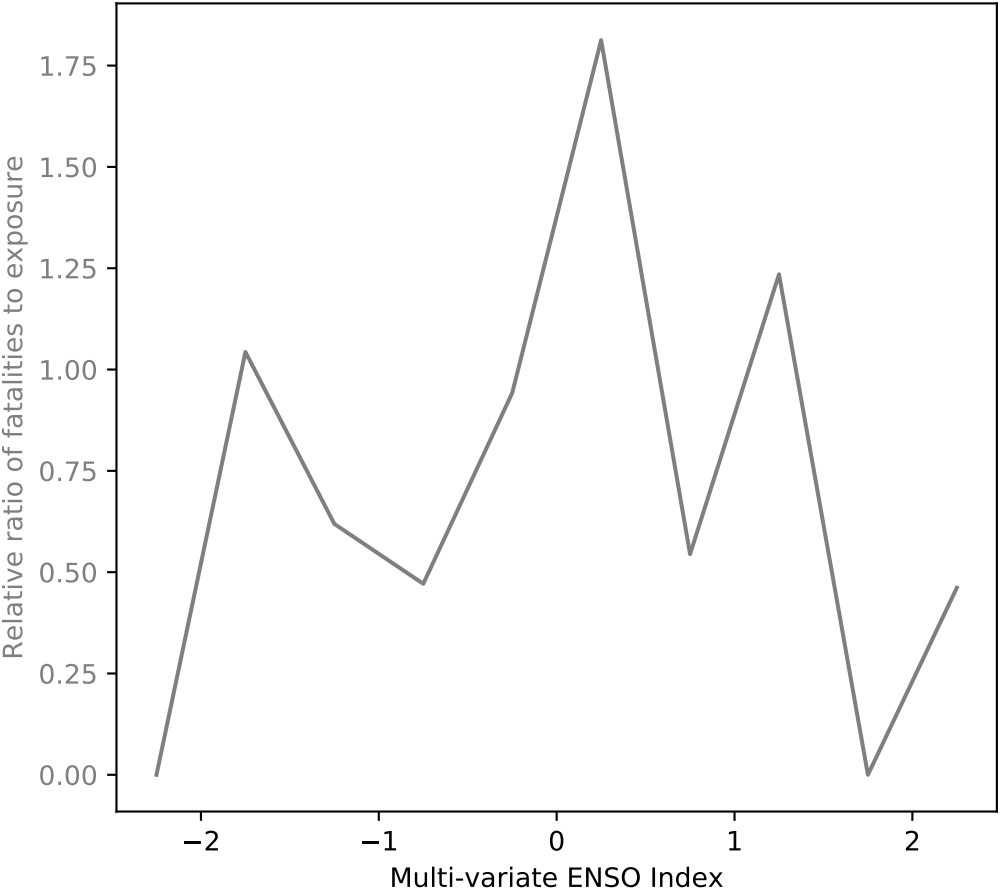

Supplement: Supplementary file 8 — Supplementary Data 5 [file 41467_2021_22398_MOESM8_ESM.zip › Sri Lanka_landslide_incidence_compare.pdf]

Fatal landslides (GFLD) vs modeled exposure for Taiwan, n= 63

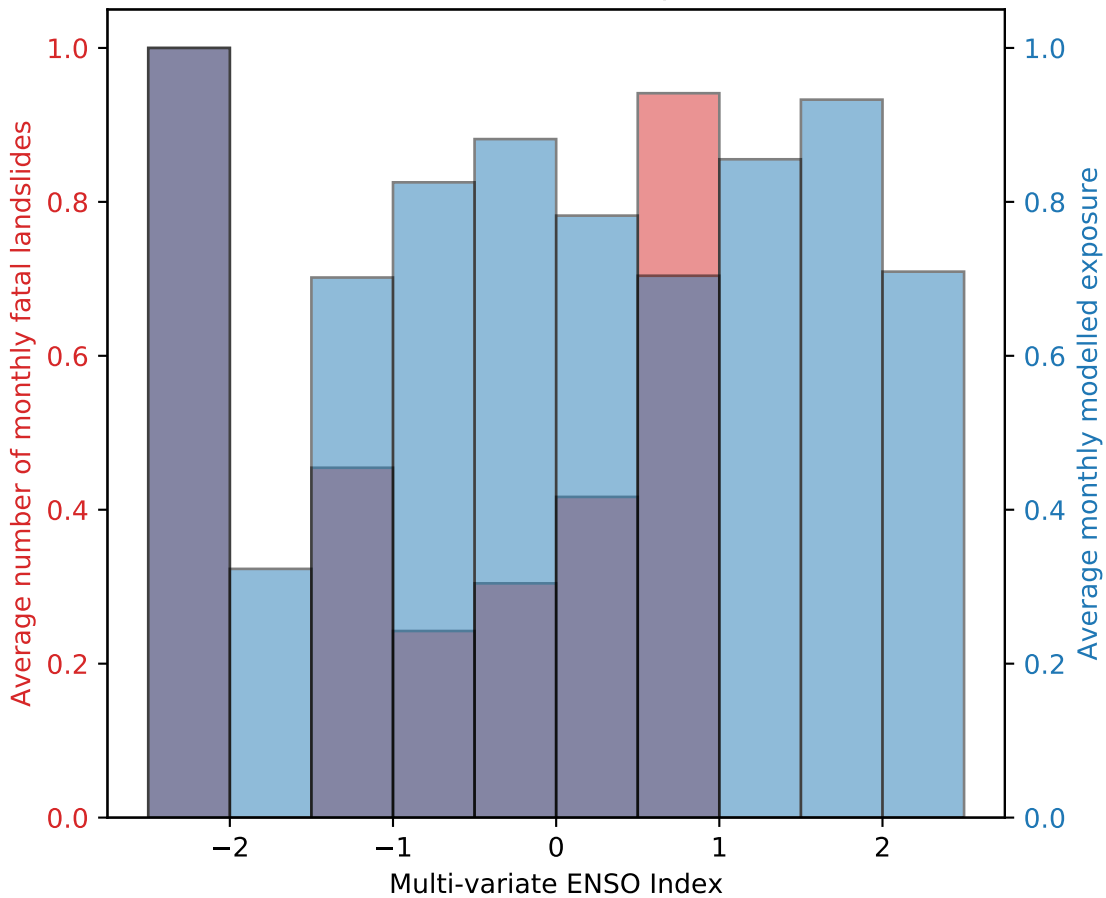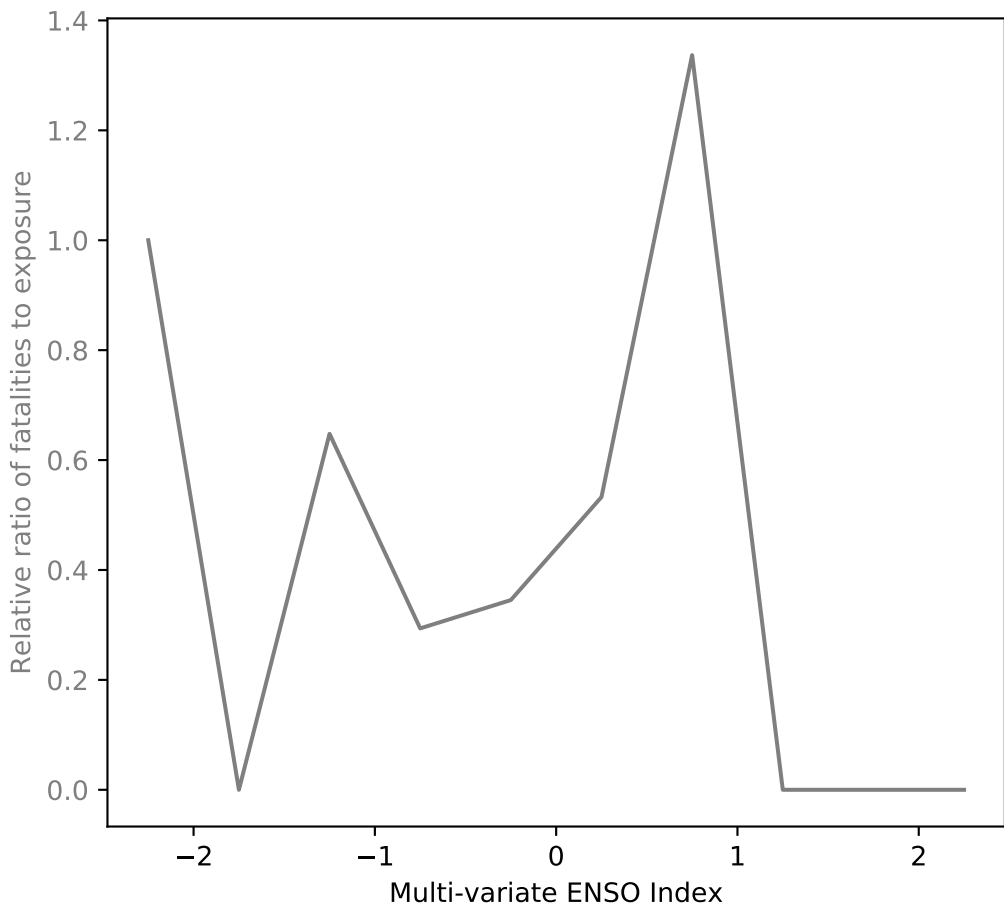

Supplement: Supplementary file 8 — Supplementary Data 5 [file 41467_2021_22398_MOESM8_ESM.zip › Taiwan_landslide_incidence_compare.pdf]

Fatal landslides (GFLD) vs modeled exposure for Tajikistan, n= 24

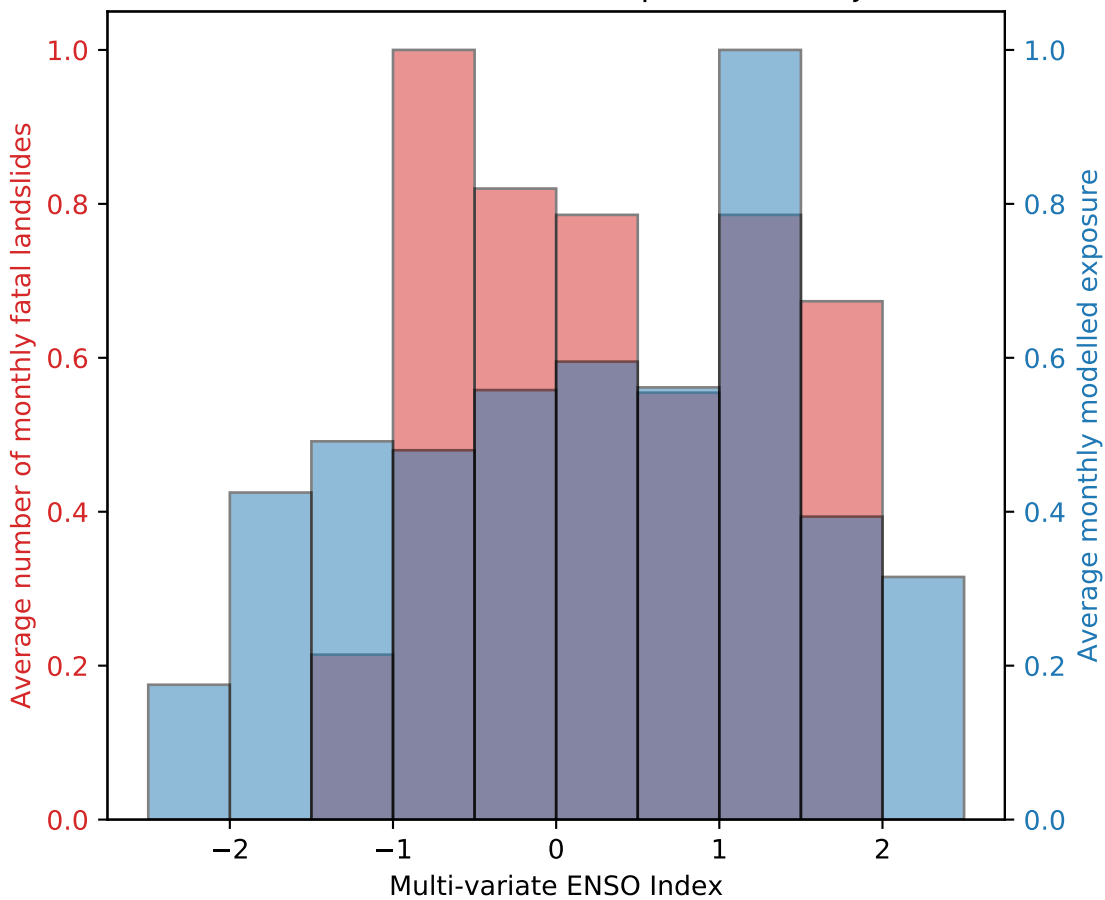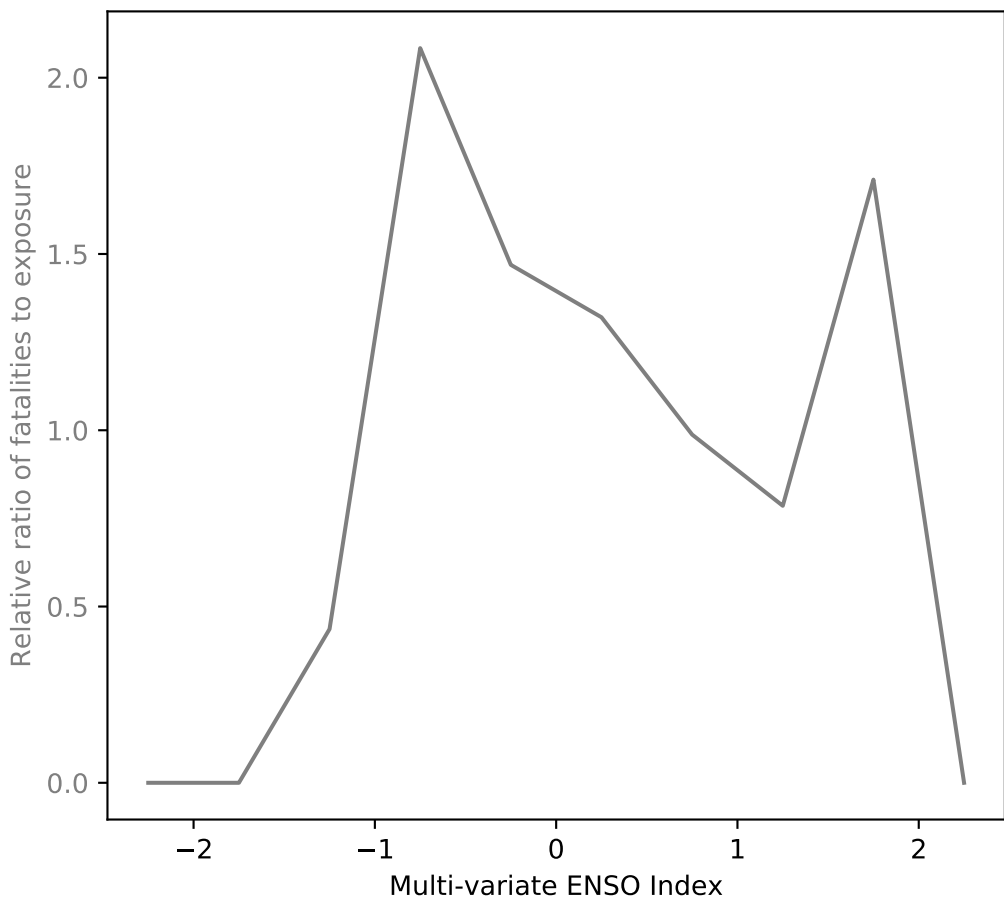

Supplement: Supplementary file 8 — Supplementary Data 5 [file 41467_2021_22398_MOESM8_ESM.zip › Tajikistan_landslide_incidence_compare.pdf]

Fatal landslides (GFLD) vs modeled exposure for Turkey, n= 53

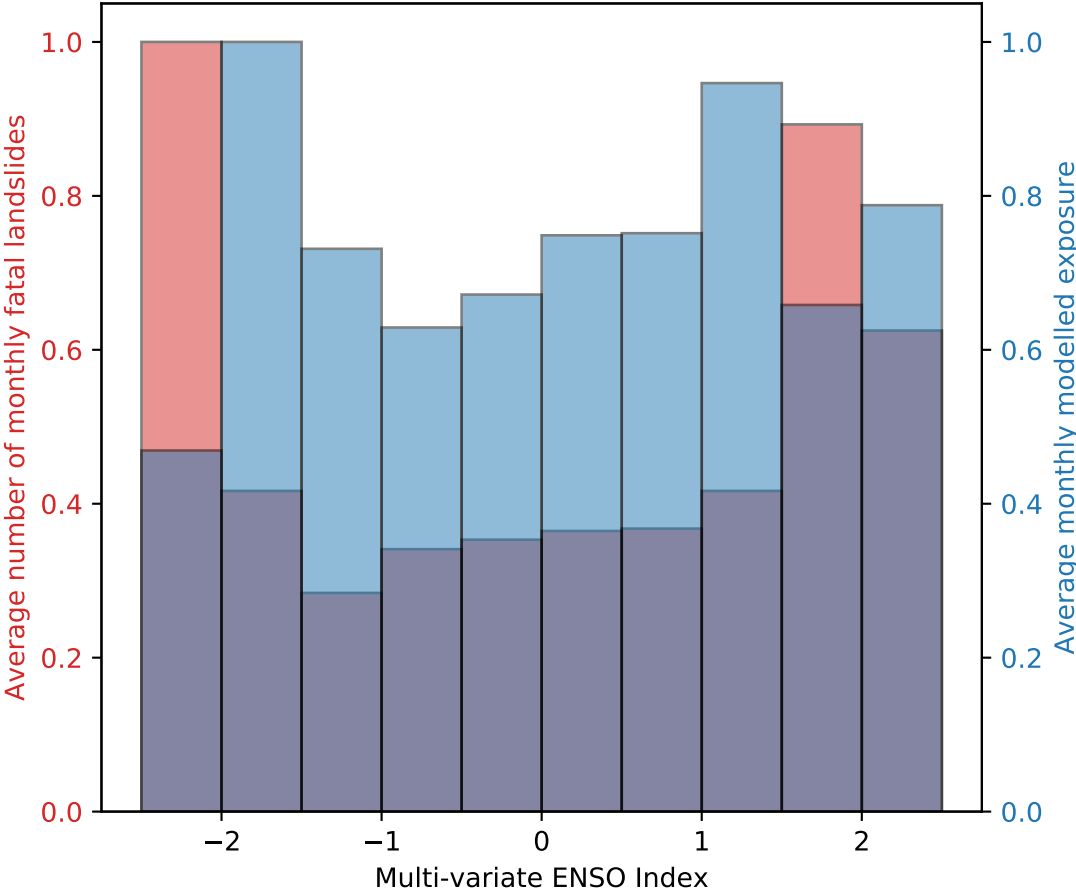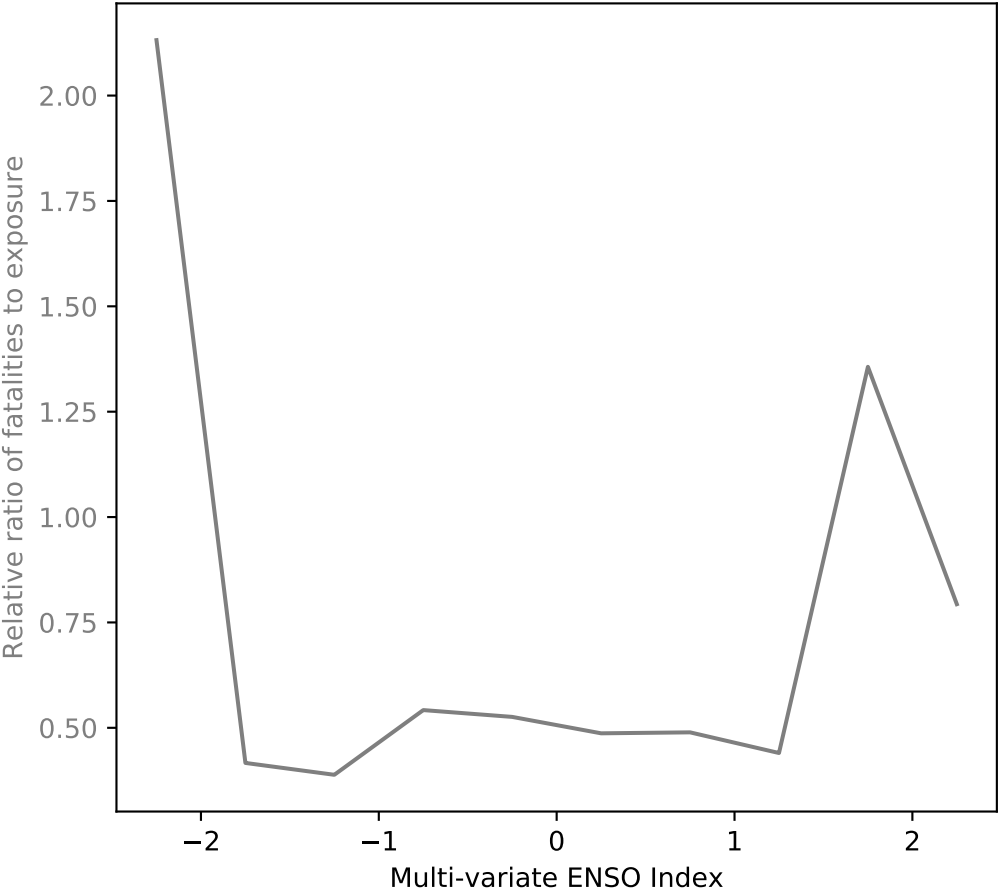

Supplement: Supplementary file 8 — Supplementary Data 5 [file 41467_2021_22398_MOESM8_ESM.zip › Turkey_landslide_incidence_compare.pdf]

Fatal landslides (GFLD) vs modeled exposure for Venezuela, n= 27

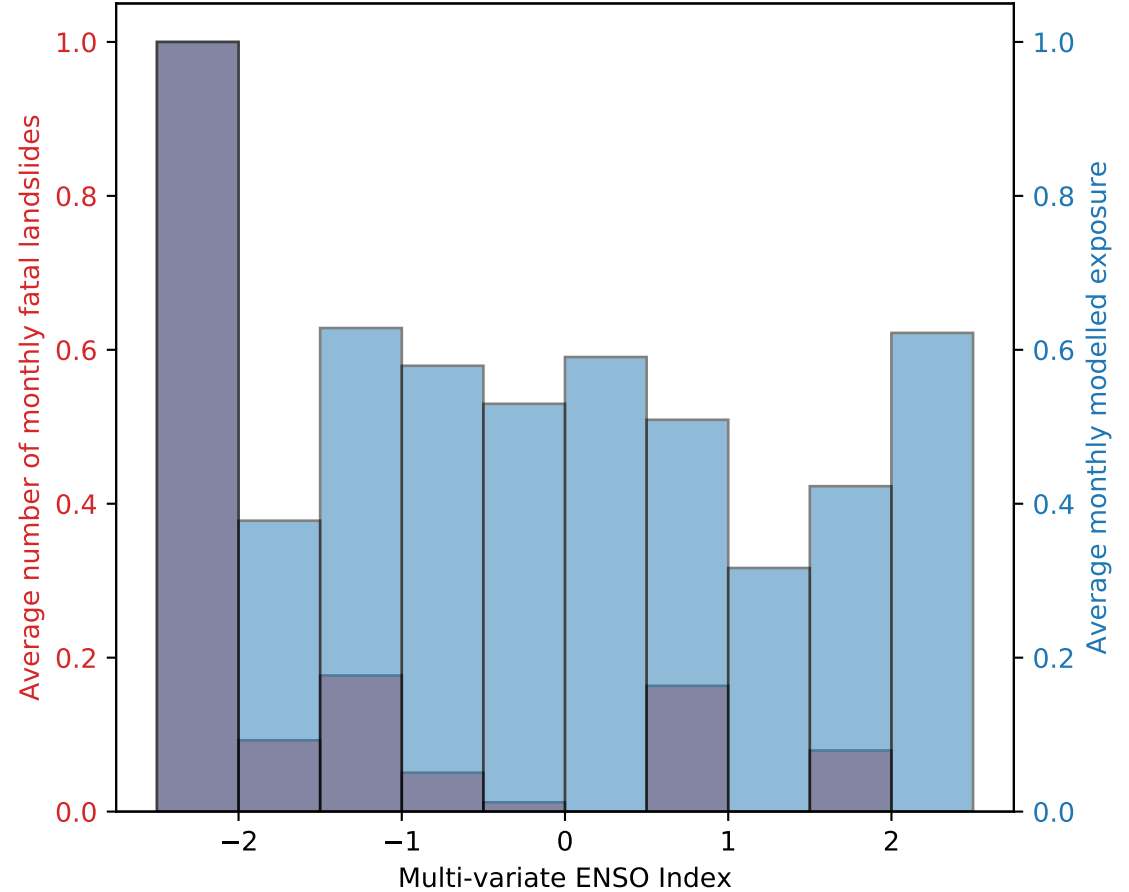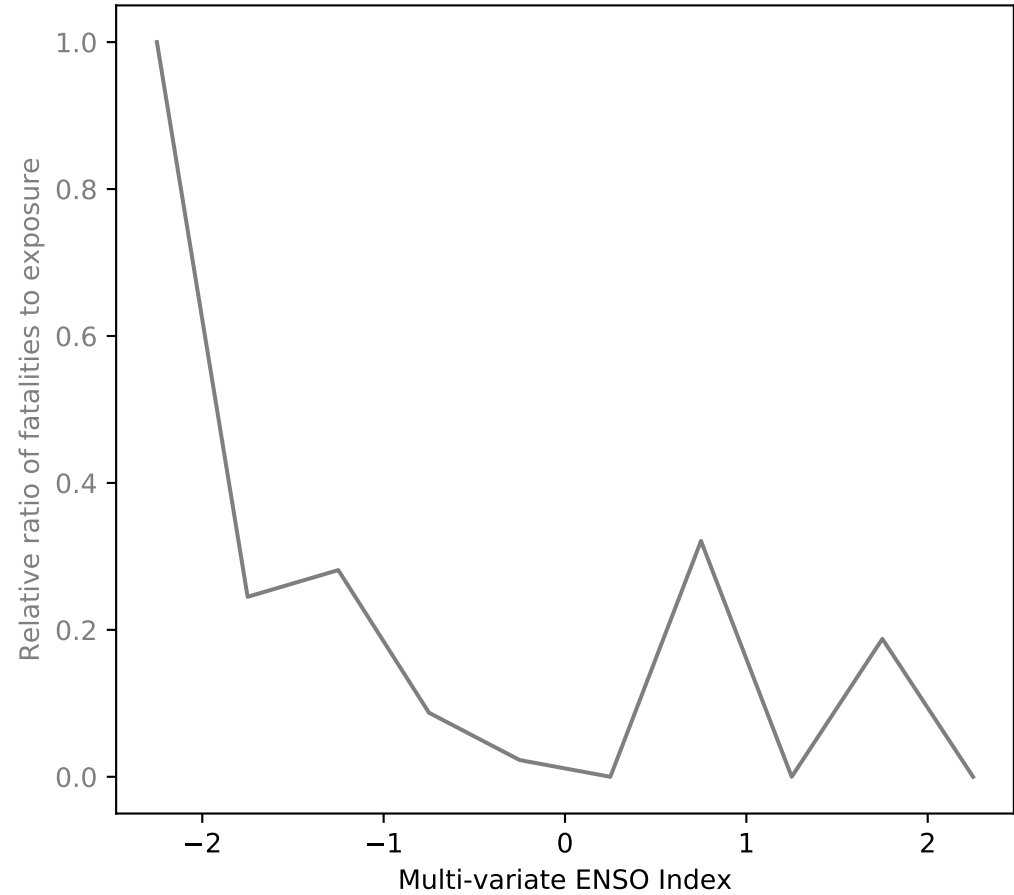

Supplement: Supplementary file 8 — Supplementary Data 5 [file 41467_2021_22398_MOESM8_ESM.zip › Venezuela_landslide_incidence_compare.pdf]
